# Integrated machine learning identifies epithelial cell marker genes for improving outcomes and immunotherapy in prostate cancer

**DOI:** 10.1186/s12967-023-04633-2

**Published:** 2023-11-04

**Authors:** Weian Zhu, Hengda Zeng, Jiongduan Huang, Jianjie Wu, Yu Wang, Ziqiao Wang, Hua Wang, Yun Luo, Wenjie Lai

**Affiliations:** 1 Department of Urology, The Third Affiliated Hospital, Sun Yat-Sen University, Guangzhou, Guangdong 510630 People’s Republic of China; 2 Laboratory of Biomaterials and Translational Medicine, The Third Affiliated Hospital, Sun Yat-Sen University, Guangzhou, Guangdong 510630 People’s Republic of China

**Keywords:** Prostate cancer, Epithelial cell, Machine learning, Immunotherapy, Prognosis

## Abstract

**Background:**

Prostate cancer (PCa), a globally prevalent malignancy, displays intricate heterogeneity within its epithelial cells, closely linked with disease progression and immune modulation. However, the clinical significance of genes and biomarkers associated with these cells remains inadequately explored. To address this gap, this study aimed to comprehensively investigate the roles and clinical value of epithelial cell-related genes in PCa.

**Methods:**

Leveraging single-cell sequencing data from GSE176031, we conducted an extensive analysis to identify epithelial cell marker genes (ECMGs). Employing consensus clustering analysis, we evaluated the correlations between ECMGs, prognosis, and immune responses in PCa. Subsequently, we developed and validated an optimal prognostic signature, termed the epithelial cell marker gene prognostic signature (ECMGPS), through synergistic analysis from 101 models employing 10 machine learning algorithms across five independent cohorts. Additionally, we collected clinical features and previously published signatures from the literature for comparative analysis. Furthermore, we explored the clinical utility of ECMGPS in immunotherapy and drug selection using multi-omics analysis and the IMvigor cohort. Finally, we investigated the biological functions of the hub gene, transmembrane p24 trafficking protein 3 (TMED3), in PCa using public databases and experiments.

**Results:**

We identified a comprehensive set of 543 ECMGs and established a strong correlation between ECMGs and both the prognostic evaluation and immune classification in PCa. Notably, ECMGPS exhibited robust predictive capability, surpassing traditional clinical features and 80 published signatures in terms of both independence and accuracy across five cohorts. Significantly, ECMGPS demonstrated significant promise in identifying potential PCa patients who might benefit from immunotherapy and personalized medicine, thereby moving us nearer to tailored therapeutic approaches for individuals. Moreover, the role of TMED3 in promoting malignant proliferation of PCa cells was validated.

**Conclusions:**

Our findings highlight ECMGPS as a powerful tool for improving PCa patient outcomes and supply a robust conceptual framework for in-depth examination of PCa complexities. Simultaneously, our study has the potential to develop a novel alternative for PCa diagnosis and prognostication.

**Supplementary Information:**

The online version contains supplementary material available at 10.1186/s12967-023-04633-2.

## Introduction

Prostate cancer (PCa) is a highly prevalent malignancy affecting males globally, with an alarming increase in incidence [1]. Current management options encompass various treatments like surgery, chemotherapy, radiotherapy, endocrine therapy, and prognostic testing methods like PSA [2]. Simultaneously, the emergence of nanomaterials and nanoparticles as promising platforms presents a new frontier in the fields of cancer therapy and regenerative medicine [3, 4]. However, due to the stark heterogeneity of PCa and the lack of effective early detection tools, more than 30% of treated patients experience biochemical recurrence (BCR) [5]. Hence, there is an emerging need for novel biomarkers capable of accurately predicting prognosis and guiding treatment decisions in precision medicine, ultimately improving patient outcomes.

In recent years, immunotherapy has made significant strides in treating solid tumors, promising enhanced patient outcomes. Numerous studies are in progress to understand tumor immune mechanisms and devise diverse treatment strategies, such as CAR T-cell therapy, immune checkpoint inhibitors, and adoptive cellular therapy [6]. However, despite the established association between immunity and the occurrence and progression of PCa [7], PCa heterogeneity poses significant challenges in identifying patients best suited for immunotherapy, resulting in limited benefits from such treatments.

PCa is believed to originate from basal or luminal epithelial cells within the prostate gland [2, 8]. The pathogenesis of PCa involves complex interactions between neighboring epithelial and stromal cells, contributing to both inter- and intra-tumoral heterogeneity [9, 10]. Addressing this heterogeneity represents a promising avenue of research, and multi-gene signatures have demonstrated potential in this context [11]. Notably, previous research has revealed heterogeneous cellular states in prostate epithelial cells characterized by elevated androgen signaling statuses that are enriched in PCa [12]. Recent advancements in sequencing technology, particularly single-cell sequencing, in combination with machine learning methods, have provided valuable insights into the complex landscape of various multi-gene panels and their roles in cancer progression [13]. Research teams have developed diverse signatures for predicting the efficacy of treatments and the prognosis of PCa, including fibroblast-derived genes signature for predicting radiotherapeutic survival [14], as well as chemokine-related prognostic genes signature relevant to anti-androgen and immunotherapies [15], among others. Still, while various prognostic models have emerged, their clinical applicability remains limited due to inadequate incorporation of machine learning algorithms, lack of model validation, and underutilization of existing data [16, 17]. Thus, the comprehensive integration of sequencing technology and machine learning methods could prove instrumental in elucidating the role of epithelial cell-related genes in PCa, driving stringent prognostic models applicable in clinical settings.

Our study endeavored to identify epithelial cell marker genes (ECMGs) through single-cell RNA sequencing (scRNA-seq). By thoroughly analyzing the prognosis, immune and clinical characteristics, and utilizing 101 machine learning-based models, we developed and validated an epithelial cell marker gene prognostic signature (ECMGPS) across several cohorts, aiming to enhance outcomes and predict therapy responses.

## Materials and methods

### Data sources and preprocessing

A total of 10 independent public datasets were employed in this research, sourced from multiple repositories including The Cancer Genome Atlas (TCGA, https://portal.gdc.cancer.gov/), Deutsches Krebsforschungszentrum (DKFZ, https://www.cbioportal.org/study/summary?id=prostate_dkfz_2018/), Memorial Sloan Kettering Cancer Center (MSKCC, https://www.mskcc.org/), Gene Expression Omnibus (GEO, https://www.ncbi.nlm.nih/), IMvigor 210 (https://research-pub.gene.com/IMvigor210CoreBiologies/), and Cancer Cell Line Encyclopedia (CCLE, https://portals.broadinstitute.org/ccle/about/): (1)GSE176031, containing 27 PCa samples with scRNA-seq data, was utilized to identify ECMGs. (2) Five datasets of TCGA-PRAD (n = 346), MSKCC (n = 134), GSE70768 (n = 110), DKFZ (n = 105), and GSE70769 (n = 92), containing complete BCR information (Additional file 2: Table S1), were employed to construct and verify our signature. (3) Two datasets of bladder cancer (TCGA-BLCA, n = 406) and renal clear cell carcinoma (TCGA-KIRC, n = 530), containing complete OS information, were used to assess the applicability of our signature in other epithelial urinary tumors. (4) The IMvigor 210 cohort, comprising 298 patients with urothelial carcinoma treated with anti-PD-L1 therapy, was used to evaluate the performance of our signature in predicting immunotherapy. (5) The CCLE dataset, with the gene expression matrix of PCa cell lines (n = 6), was employed to select suitable cell lines for subsequent experiments. Among them, the RNA-seq data was transformed into TPM and log2.

### Identification of ECMGs

ECMGs were identified using the Seurat package in R for object creation and cell filtration to ensure high-quality cells. Filters were applied to exclude genes detected in fewer than three cells, cells with fewer than 50 detectable genes, or cells with over 5% mitochondrial genes. The gene profiles were then normalized, and principal component analysis (PCA) was performed on the 1500 most variable genes identified through JackStraw analysis. Afterward, the data was clustered using the FindClusters function in R, with a resolution parameter of 0.5. Visualization employed the t-distributed stochastic neighbor embedding (t-SNE) algorithm. Marker genes (adjusted *P* value < 0.05 and |log FC|> 1) for each cluster were identified using the FindAllMarkers function in conjunction with the Wilcoxon-Mann–Whitney test, which compared the differences in gene expression between a cluster and all other clusters. Additionally, the SingleR package was utilized to annotate and visualize the cell types.

### Consensus clustering analysis

Agglomerative pam clustering, along with a 1-Pearson correlation distance metric and 80% sample resampling for 1000 repetitions, was employed to classify patients in the TCGA cohort into distinct clusters based on ECMGs expression profiles. The optimal number of clusters was established using the cumulative distribution function (CDF), the relative change in the area under the CDF curve, and the consistency matrix. To assess the differences and biochemical recurrence-free survival (bRFS) rates between the different clusters, the PCA and Kaplan–Meier method were employed. Additionally, the association between the clusters and clinicopathological features such as age, Gleason score, pathological N (pN) stage, PSA levels, and pathological T (pT) stage were examined using the chi-square test. Moreover, the presence of copy number variation (CNV) was compared between the clusters to explore potential genomic differences.

### Gene set variation analysis (GSVA)

The gene expression profile of ECMGs in the TCGA cohort was estimated through the implementation of the GSVA package in R, using the single-sample gene set enrichment analysis (ssGSEA) algorithm. The gene sets utilized in this estimation were obtained from the Gene Ontology and the Kyoto Encyclopedia of Genes and Genomes. Subsequently, the enrichment score for each pathway within the gene sets was computed. Differential enrichment scores of pathways between the clusters were determined, and the top 15 pathways with statistical significance (adjusted *P* value < 0.05) for each cluster were used to generate heatmaps.

### Tumor immune microenvironment analysis

First, the tumor purity, stromal score, immune score, and ESTIMATE score of the two clusters were estimated and subsequently compared [18]. Second, the ssGSEA algorithm and the Mann–Whitney test were employed to calculate the scores of immune infiltrations for 29 immune cell types or pathways, which were obtained from a previous study [19] (Additional file 2: Table S2). To ensure the robustness and stability of the results of ssGSEA, seven other algorithms including TIMER [20], CIBERSORT, CIBERSORT-ABS [21], QUANTISEQ [22], MCPCOUNTER [23], XCell [24], and EPIC [25] were also used. Furthermore, the scores of the steps in the cancer-immunity cycle, based on ECMGs, were analyzed using Tracking Tumor Immunophenotype (https://biocc.hrbmu.edu.cn/TIP/), following the approach employed in a previous study [26]. Third, the differences between the two clusters were compared for 145 marker genes of immune modulators, including immunostimulators (n = 42), immunoinhibitors (n = 23), MHC (n = 21), receptors (n = 18), and chemokines (n = 41), obtained from a previous study [27].

### Machine learning-based signature construction and validation

A comprehensive approach was employed, which involved the integration of 101 different combinations of 10 distinct machine learning algorithms. The objective was to construct a prognostic signature with remarkable accuracy and stability. The 10 original machine learning algorithms utilized in this study were CoxBoost, elastic network (Enet), survival support vector machine (survival-SVM), Lasso, partial least squares regression for Cox (plsRcox), Ridge, random survival forest (RSF), stepwise Cox, supervised principal components (SuperPC), and generalized boosted regression modeling (GBM). Notably, some of these algorithms, including CoxBoost, Lasso, RSF, and stepwise Cox, possessed feature selection capabilities.

The specific machine learning process employed in this study was summarized as follows: (1) Initial Identification of Prognostic ECMGs: We identified ECMGs with prognostic potential in the TCGA cohort through univariate Cox regression analysis. (2) Leave-One-Out Cross-Validation Framework: Subsequently, we implemented a leave-one-out cross-validation framework within the TCGA cohort, executing 101 models on the candidate prognostic ECMGs. The aim was to develop a prediction signature that exhibits robustness and reliability. (3) Rigorous Testing in Independent Validation Cohorts: To evaluate the performance of the constructed signatures, we subjected them to rigorous testing in four independent validation cohorts. (4) Selection of Optimal Model: For each model, we calculated Harrell's concordance index (C-index) across all cohorts. The model with the highest mean C-index was identified as the optimal model.

Using the optimal model, patients were stratified into either high-risk or low-risk groups based on the median risk scores derived from both the TCGA cohort and the four independent validation cohorts. Subsequently, t-SNE and PCA methods were used to assess the distribution of the optimal model in the training cohort and validation cohorts, respectively, across these two groups. The prognostic value and predictive accuracy of the optimal model were evaluated using receiver operating characteristic (ROC) curves and Kaplan–Meier curves.

### Evaluation of the clinical significance of ECMGPS

The distinctions between ECMGPS risk scores and clinicopathological characteristics were evaluated using the chi-square test. Subsequently, stratified survival analysis was conducted for the subgroups. To identify independent prognostic factors, both univariate and multivariate Cox regression analyses were performed. Additionally, to investigate the applicability of ECMGPS in other urothelial tumors, we obtained mRNA expression and survival data from the TCGA database for bladder cancer and renal clear cell carcinoma. These datasets were further analyzed using Kaplan–Meier curves.

### Comparison of published signatures in PCa

By conducting a comprehensive literature search on PubMed (https://pubmed.ncbi.nlm.nih.gov/) on published model articles predicting PCa outcomes up until June 1, 2023, we gathered published signatures for performance comparison with ECMGPS (excluding miRNA signatures due to limited miRNA information in the validation cohorts). These collected signatures were fitted using various algorithms, such as Lasso and RSF, and encompassed diverse biological significance. Subsequently, risk scores were calculated for the five cohorts using the genes or RNA and coefficients provided in the respective articles. The performance in predicting BCR of PCa was then compared using the C-index.

### Immunotherapy response and drug sensitivity

Initially, somatic mutation data from TCGA, which were processed using the VarScan platform, were analyzed to compare the mutation profiles between the two groups. Subsequently, the IMvigor 210 cohort, which received anti-PD-L1 treatment, was used to evaluate the differences in response to immunotherapy and survival outcomes between the low-risk and high-risk groups classified by ECMGPS. Additionally, the tumor immune dysfunction and exclusion (TIDE) algorithm was utilized to predict the responsiveness of ECMGPS to immune checkpoint inhibitors. Finally, the efficacy of 10 commonly used anticancer drugs between the two subgroups was determined based on the IC50 values obtained from the Genomics of Drug Sensitivity in Cancer database (https://www.cancerrxgene.org/) [28].

### Cell culture and immunohistochemistry (IHC)

PCa cells of LNCaP and VCaP were procured from the American Type Culture Collection (Manassas, VA, USA). The cells were cultured in RPMI medium (HyClone, USA) or DMEM medium (HyClone, USA), both of which were supplemented with 10% fetal bovine serum (Bovogen, Australia). The cells were incubated in a humidified atmosphere at 37 °C with 5% CO_2_. Regular passage of cells was performed, and routine checks were conducted to ensure the absence of mycoplasma contamination.

To assess the protein expression levels of transmembrane p24 trafficking protein 3 (TMED3) between PCa and normal samples, IHC data and images obtained from the Human Protein Atlas (HPA, https://www.proteinatlas.org/) were utilized for analysis and comparison.

### RNA interference

The lentivirus containing the TMED3-knockdown sequence and the control lentivirus were designed and synthesized by GenePharm Company (Shanghai, China). Following the instructions provided in the virus manual, lentivirus transfection was performed on LNCaP and VCaP cells separately. The target sequences of TMED3-RNAi were TMED3-RNAi1: 5ʹ-CACCATCTACAGAGAAACGAA-3ʹ, and TMED3-RNAi2: 5ʹ-TACGATGTTGACTGCTATGTA-3ʹ.

### Western blotting

The western blotting procedure was carried out following the established protocol as previously described [29]. In brief, cells were collected, lysed using lysis buffer, and then centrifuged to gather the supernatant. Subsequently, proteins were separated via 10% SDS-PAGE and transferred onto a 0.45 μm PVDF membrane (Merck Millipore, USA). After blocking with 4% BSA at room temperature for 1 h, the membrane was subjected to incubation with the primary antibody overnight at 4 °C, followed by incubation with HRP-conjugated secondary antibody at room temperature for 1 h the next day. Finally, signals corresponding to the target proteins were detected using the ChemiDoc MP system (Bio-Rad, USA) and ECL (Advansta, USA). The primary antibodies used were anti-TMED3 (Abcam, ab223175, USA) and anti-α-Tubulin (Abcam, ab176560, USA).

### Cell counting kit-8 (CCK-8) assay

The CCK-8 assay (Dojindo, Japan) was conducted following the previously described method [29]. Briefly, LNCaP and VCaP cells were seeded in 96-well plates (2 × 10^3^ cells/per well). After incubation at 37 °C with 5% CO_2_, the absorbance at 450 nm was measured using a microplate reader (Bio-Rad, USA).

### Colony formation assay

LNCaP or VCaP cells and the corresponding negative control group were inoculated into 6-well plates (1 × 10^3^ cells/per well). After two weeks of culture, cells were washed three times with PBS, fixed with 4% paraformaldehyde for 15 min, and then stained with 0.5% crystal violet for 10 min. Visible colonies were counted and statistically analyzed under a microscope.

### EdU assay

To detect the proliferative ability of PCa cells, an EdU kit (RiboBio, China) was used according to the kit instructions. Subsequently, the visible cells were counted using a fluorescence microscope (Olympus Optical).

### Flow cytometry

Apoptosis was analyzed by flow cytometry using the previously described method [30]. PCa cells and the corresponding negative control cell suspension were treated with 5 µl FITC Annexin V and 5 µl PI (Becton, Dickinson and Company) and incubated at room temperature for 15 min. Apoptosis was then detected by flow cytometry (Calibur, BD Bioscience). FlowJo software (Tree Star) was used for result analysis.

### Statistical analysis

Data analysis and graphical visualization were conducted using R (version 4.2.1) or SPSS (version 25.0). All experiments were repeated at least three times, and the results were presented as the mean ± standard deviation. The Pearson test was used to assess the correlation between two continuous variables. Categorical variables were compared using the chi-squared test, while the Wilcoxon rank-sum test or t test was employed for comparing continuous variables. *P* value less than 0.05 was considered statistically significant (*P < 0.05, **P < 0.01, ***P < 0.001, ns: not significant).

## Results

### Identification of ECMGs by scRNA-seq

**Fig. 1:**
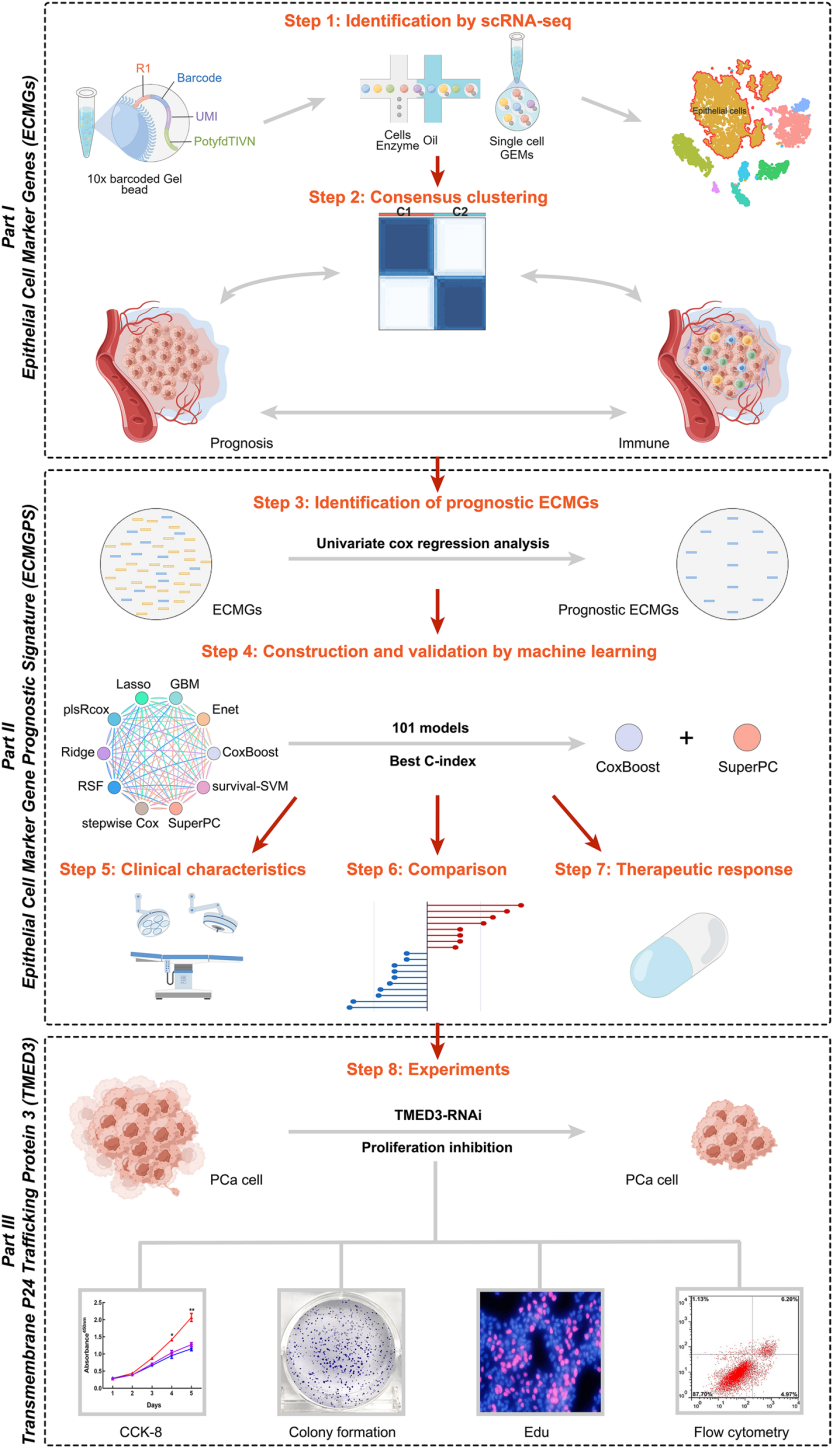
The workflow of this study. Integrating muti-omics analysis and machine learning, an epithelial cell marker gene prognostic signature (ECMGPS) was developed and validated from epithelial cell marker genes (ECMGs) to predict prognosis and treatment response

**Fig. 2:**
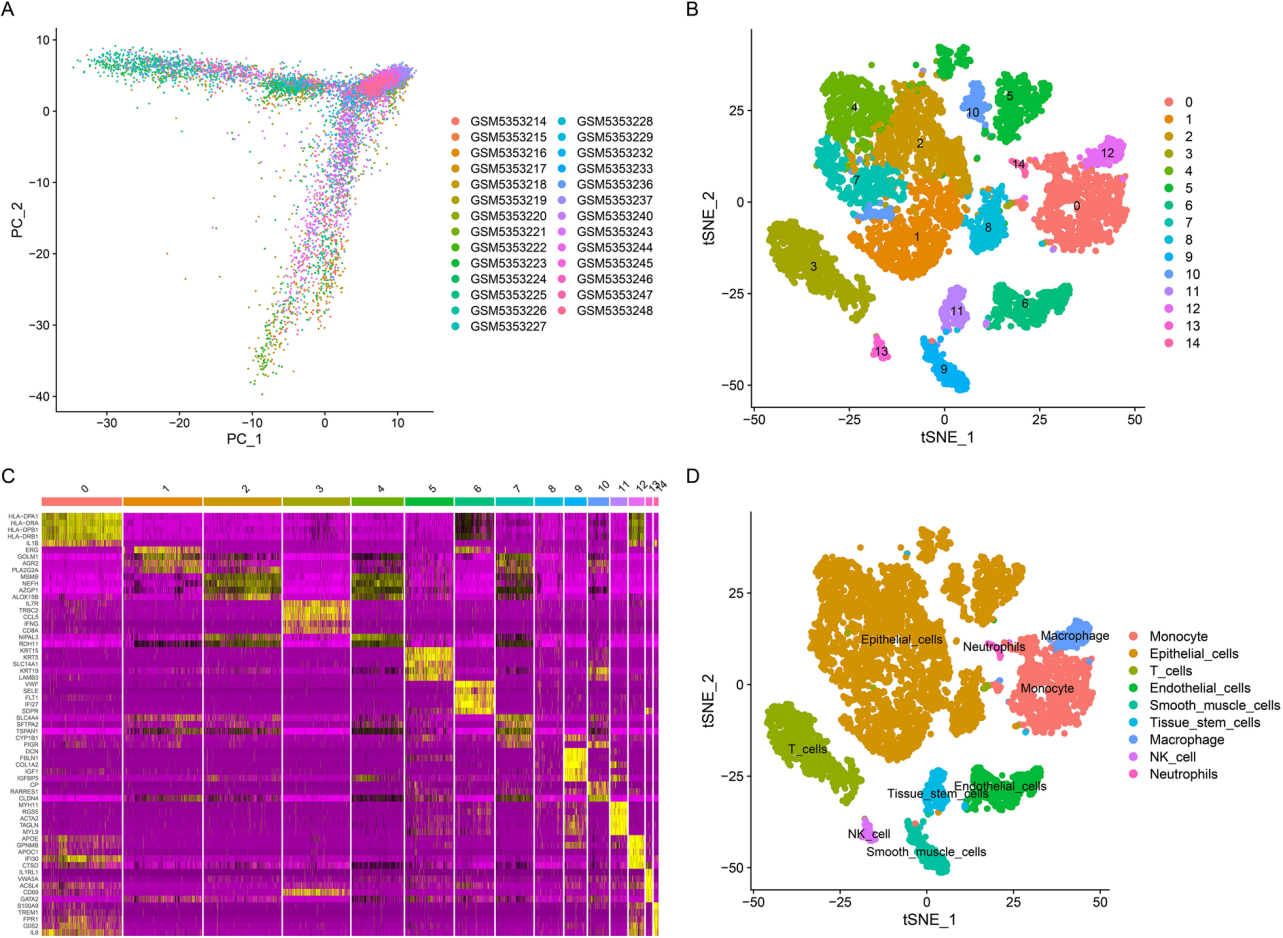
Identification of ECMGs through single-cell RNA sequencing analysis. **A** Principal component analysis (PCA) plot showing 27 samples. **B** t-distributed stochastic neighbor embedding (t-SNE) plot showing 15 clusters of cell types. **C** Heatmap showing the top 5 marker genes within each of the 15 clusters. **D** t-SNE plot showing 9 cell types

The research flowchart is presented in Fig. 1. Through analysis of scRNA-seq data from GSE176031, we reduced dimensionality by running PCA on 1500 variable genes across 27 PCa samples (Fig. 2A). Fifteen cell clusters were identified, with clusters 1, 2, 4, 5, 7, 8, and 10 classified as epithelial cells (Additional file 2: Table S3). Subsequently, 543 marker genes were identified as differentially expressed in the epithelial cell cluster and designated ECMGs for further research (Fig. 2B–D).

### Establishment of ECMGs consensus clusters and their relationship with prognosis

**Fig. 3:**
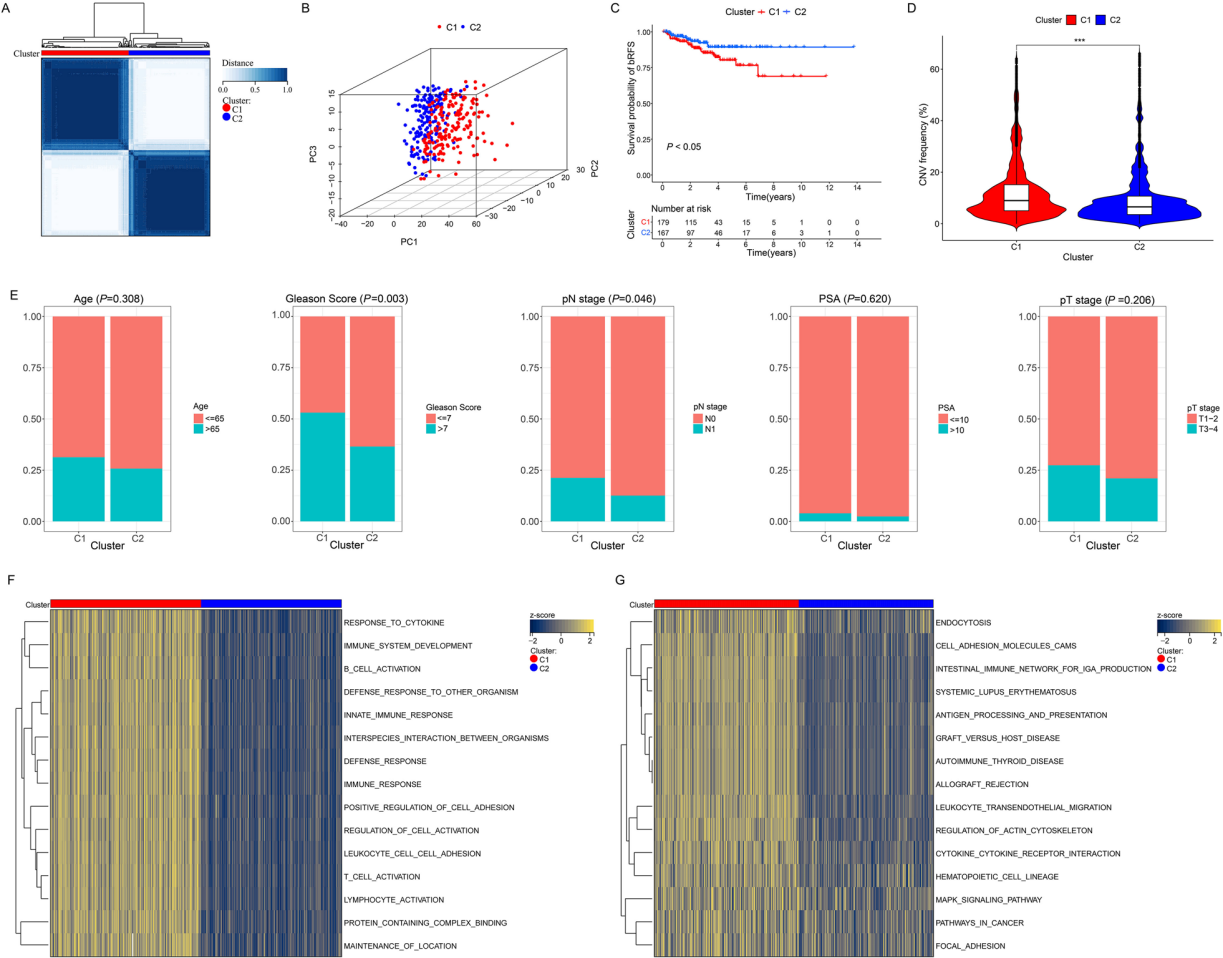
Prognostic association of ECMGs classifications. **A** Consensus clustering matrix of 543 ECMGs categorized into two clusters (C1 = 179, C2 = 167). **B** 3D-PCA plot showing the distribution between C1 and C2. **C** Kaplan–Meier curves for biochemical recurrence-free survival (bRFS) in C1 and C2. **D** Violin plot comparing the copy number variation (CNV) frequency between C1 and C2. **E** Composition percentage of the two clusters in clinicopathological features, including age, Gleason score, pN stage, PSA, and pT stage. **F** Gene set variation analysis (GSVA) of the Gene Ontology in C1 and C2. **G** GSVA analysis of the Kyoto Encyclopedia of Genes and Genomes in C1 and C2. ***P < 0.001

Utilizing the expression profiles of ECMGs in the TCGA dataset, PCA successfully distinguished between normal and PCa samples (Additional file 1: Figure S1A), suggesting distinct regulatory roles of epithelial cells in PCa and normal. Subsequently, we performed clustering analysis on the ECMGs expression levels of patients in the TCGA cohort. By evaluating the area under the curve of the CDF, the downward trend of CDF delta, and average consistency within patient clusters (Additional file 1: Figure S1B-D), we successfully identified two optimal clusters (k = 2, C1 = 179, C2 = 167) (Fig. 3A). Subsequently, a distinct clustering pattern was revealed through further PCA (Fig. 3B), indicating a significant differential distribution of ECMGs in the two identified clusters.

The survival analysis revealed that C1 had a poorer prognosis than C2 (Fig. 3C). Considering that CNV serve as crucial markers of malignant tumor progression [31], we investigated the frequency of CNV in both the C1 and C2 patient groups. Remarkably, C1 patients displayed a significantly higher frequency of CNV than C2 patients (Fig. 3D), further implying that C1 exhibits a more aggressive behavior and worse prognosis in PCa. In addition, C1 patients experienced higher Gleason score and advanced pN stage (Fig. 3E), further supporting C1 as a highly malignant subgroup. Our further investigation using the GSVA algorithm unveiled that several signaling pathways, mainly related to tumorigenesis and development, were significantly enriched in C1. Notably, C1 exhibited a higher abundance of immunomodulatory signaling pathways and functions than C2 (Fig. 3F, G). Therefore, these results suggest that immune characteristics might exert a substantial influence on the malignancy and poor prognosis of ECMGs in PCa.

### Immunophenotypic analysis based on ECMGs clusters

**Fig. 4:**
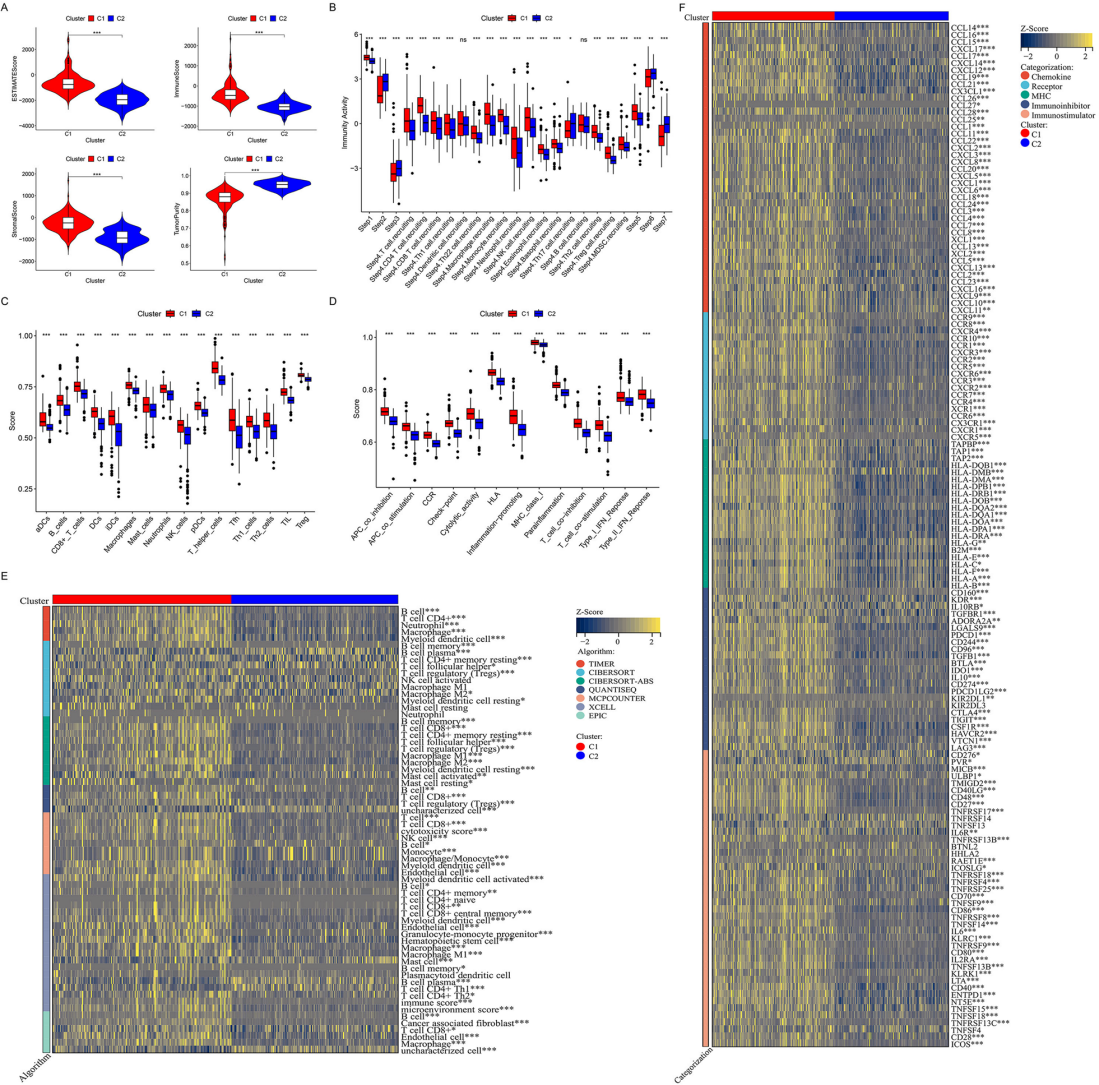
Immune association of ECMGs classifications. **A** Violin plots comparing the ESTIMATE score, stromal score, immune score, and tumor purity between C1 and C2. **B** Box plot comparing the activity scores of the steps in the cancer-immunity cycle between C1 and C2. **C** Box plot comparing scores for 16 immune cell types between C1 and C2. **D** Box plot comparing scores for 13 immune-related functions between C1 and C2. **E** Verification of ssGSEA results by seven other algorithms, namely TIMER, CIBERSORT, CIBERSORT-ABS, QUANTISEQ, MCPCOUNTER, XCell, and EPIC. **F** Heatmap showing the correlation between the two clusters and immune modulators. *P < 0.05, **P < 0.01, ***P < 0.001. *ns* not significant

C1 and C2 exhibited noteworthy disparities in immune infiltration and the various stages of the cancer-immunity cycle. Specifically, C1 displayed significantly higher stromal score, immune score, and ESTIMATE score, with the exception of tumor purity, than C2 (Fig. 4A). Additionally, C1 demonstrated markedly enhanced overall infiltration abundance of immune-related cells and pathways, as well as increased immune activity, encompassing 16 out of 23 steps in the cancer-immunity cycle, in comparison to C2 (Fig. 4B–D). To ensure the impartiality of our analytical algorithm in producing these two clusters, we employed seven additional algorithms, namely CIBERSORT-ABS, CIBERSORT, EPIC, MCPCOUNTER, QUANTISEQ, TIMER, and XCell to validate the stability and reliability of the ssGSEA results (Fig. 4E). Notably, C1 also exhibited higher expression of most immune modulators than C2 (Fig. 4F). These findings indicate that heightened immune patterns contribute to the malignancy and poor prognosis of ECMGs in PCa. Consequently, we classified C1 as immunologically hot tumors and C2 as immunologically cold tumors.

### Construction of a prognostic signature based on machine learning

**Fig. 5:**
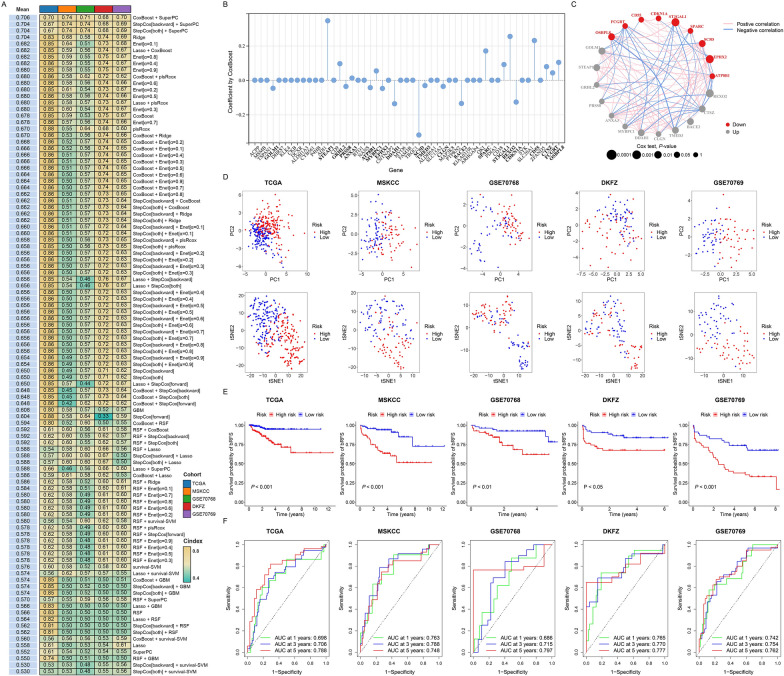
Construction and validation of ECMGPS based on machine learning. **A** C-index of 101 prediction models using 10 machine learning algorithms across five cohorts. **B** Coefficients of 21 model genes obtained from 51 prognostic ECMGs using the CoxBoost algorithm. **C** Correlation network among the 21 model genes in PCa. Line thickness represents the strength of association, and color indicates the direction of the association. Dot size reflects the effect of each gene on prognosis, while color denotes gene expression. **D** PCA and t-SNE plots showing the distribution of low- and high-risk groups. **E** Kaplan–Meier curves showing bRFS in the low- and high-risk groups. **F** Receiver operating characteristic (ROC) curves showing 1-, 3-, and 5-year bRFS in the low- and high-risk groups

To develop a prognostic signature using the expression profiles of 543 ECMGs, we conducted an initial univariate Cox regression analysis, which identified 51 prognostic ECMGs associated with bRFS (Additional file 2: Table S4). Subsequently, we fitted 101 prediction models using 10 different machine learning algorithms, which included CoxBoost, Enet, GBM, Lasso, plsRcox, Ridge, RSF, stepwise Cox, SuperPC, and survival-SVM. To evaluate the robustness of these models and determine the most effective prognostic signature with the highest mean C-index, we employed a tenfold cross-validation approach. The evaluation was conducted on the TCGA training cohort and four external validation cohorts (MSKCC, GSE70768, DKFZ, and GSE70769) (Fig. 5A).

Notably, the combination of the CoxBoost and SuperPC algorithms exhibited the highest mean C-index (0.706) and was selected as the optimal model. During the machine learning process, the CoxBoost algorithm identified 21 genes of utmost value, and these genes were further optimized using the SuperPC algorithm, enhancing the model performance and resulting in the development of a highly reliable prognostic model known as ECMGPS (Fig. 5B). Out of the 21 genes, 9 were downregulated, while the remaining 12 were upregulated (Fig. 5C).

### Evaluation of the predictive performance of ECMGPS

To comprehensively assess the robustness of ECMGPS, we calculated risk scores for each patient and categorized them into either low-risk or high-risk groups. Both PCA and t-SNE analyses demonstrated two distinct distribution trends among samples in the low-risk and high-risk groups, which consistently held across the four independent validation cohorts (Fig. 5D). Additionally, Kaplan–Meier analysis demonstrated that the low-risk group had significantly better bRFS than the high-risk group in the TCGA cohort (*P* < 0.001), and similar results were observed in the MSKCC cohort (*P* < 0.001), GSE70768 cohort (*P* < 0.01), DKFZ cohort (*P* < 0.05) and GSE70769 cohort (*P* < 0.001) (Fig. 5E). Consistently, the areas under the ROC curve (AUCs) for 1-, 3-, and 5-year bRFS were 0.698, 0.706, and 0.788 in the TCGA cohort; 0.763, 0.788, and 0.748 in the MSKCC cohort; 0.686, 0.715, and 0.797 in the GSE70768 cohort; 0.765, 0.770, and 0.777 in the DKFZ cohort; and 0.742, 0.754, and 0.762 in the GSE70769 cohort, respectively (Fig. 5F). These results demonstrate that ECMGPS accurately predicts the prognosis of PCa patients. Collectively, these findings indicate that ECMGPS exhibits stable and robust performance across multiple independent cohorts.

### Evaluation of the clinical features of ECMGPS

**Fig. 6:**
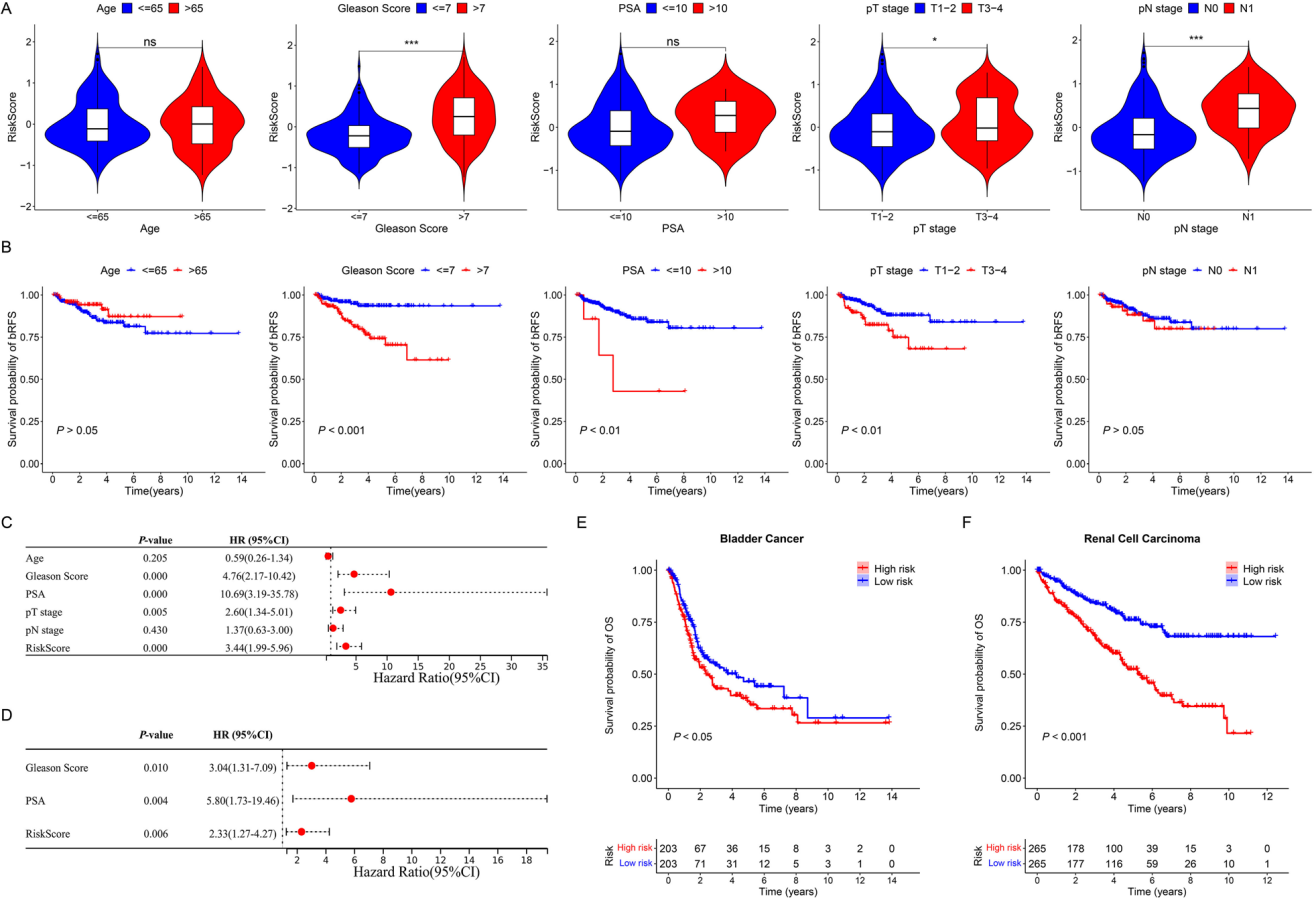
Evaluation of the clinical independence and application value of ECMGPS in the TCGA cohort. **A** Violin plots comparing the risk scores between different subgroups stratified by clinicopathological features. **B** Kaplan–Meier curves for bRFS stratified by clinicopathological features. **C** Univariate Cox regression analysis of ECMGPS in relation to bRFS. **D** Multivariate Cox regression analysis of ECMGPS in relation to bRFS. **E** Kaplan–Meier curves for overall survival (OS) in bladder cancer. **F** Kaplan–Meier curves for OS in renal cell carcinoma. *P < 0.05, ***P < 0.001. *ns* not significant

To investigate the association between the prognostic significance of the ECMGPS and clinicopathological characteristics, we conducted a comparative analysis of risk scores across different stratified characteristics. Intriguingly, patients with high-risk scores in the TCGA cohort exhibited higher Gleason score (*P* < 0.001), a finding that was consistently confirmed in four independent validation cohorts: MSKCC cohort (*P* < 0.05), DKFZ cohort (*P* < 0.001), GSE70768 cohort (*P* < 0.05), and GSE70769 cohort (*P* < 0.01) (Fig. 6A and Additional file 1: Figure S2A, S3A, S4A, S5A). Notably, Gleason score was the only clinical feature that displayed statistically significant differences across all cohorts. Additionally, stratified survival analysis revealed that patients with advanced Gleason score experienced worse bRFS in all cohorts (TCGA cohort: *P* < 0.001, MSKCC cohort: *P* < 0.001, DKFZ cohort: *P* < 0.001, GSE70768 cohort: *P* < 0.001, and GSE70769 cohort: *P* < 0.001) (Fig. 6B and Additional file 1: Figure S2B, S3B, S4B, S5B). These findings shed light on the potential underlying factors contributing to the unfavorable prognosis observed in the high-risk group of ECMGPS.

To assess the independent predictive value of ECMGPS, we first conducted univariate Cox regression analysis (Fig. 6C and Additional file 1: Figure S2C, S3C, S4C, S5C). Subsequently, we further included significant variables in the multivariate Cox regression analysis (Fig. 6D and Additional file 1: Figure S2D, S3D, S4D, S5D). Remarkably, we observed that only ECMGPS-based risk score consistently exhibited statistical significance for predicting bRFS across all cohorts, indicating its potential as an independent risk factor for BCR in PCa. Moreover, considering the outstanding predictive ability of ECMGPS in PCa, we extended our investigation to evaluate its performance in several other common epithelial-derived urinary tumors using the TCGA database. Surprisingly, the Kaplan–Meier survival curves for patients in the high-risk group exhibited significantly lower OS in both bladder cancer (*P* < 0.05) and renal cell carcinoma (*P* < 0.01) (Fig. 6E, F). These findings suggest that ECMGPS, originally developed as a biomarker for PCa, holds great potential for broader applications in diverse tumor types.

### Comparison of ECMGPS with 80 previously published signatures in PCa

**Fig. 7:**
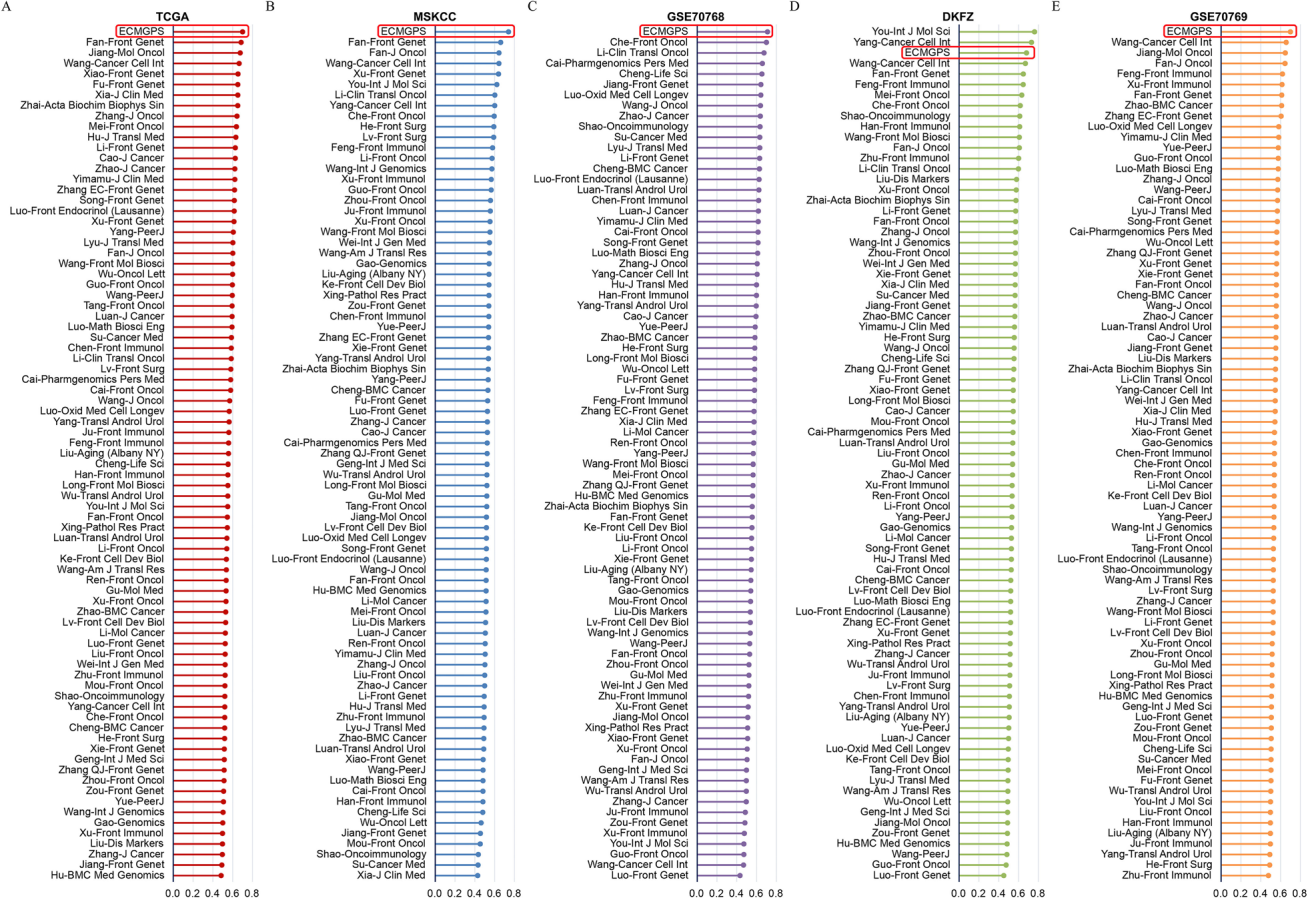
Comparison between ECMGPS and 80 previously published signatures. **A** C-index comparison of ECMGPS and 80 previously published signatures in the TCGA cohort. **B** C-index comparison of ECMGPS and 80 previously published signatures in the MSKCC cohort. **C** C-index comparison of ECMGPS and 80 previously published signatures in the GSE70768 cohort. **D** C-index comparison of ECMGPS and 80 previously published signatures in the DKFZ cohort. **E** C-index comparison of ECMGPS and 80 previously published signatures in the GSE70769 cohort

With the rapid advancement of big data technologies, such as high-throughput sequencing and machine learning, an increasing number of prognostic signatures have been developed for accurate medical care of cancer patients. To comprehensively compare the performance of ECMGPS with other signatures in predicting BCR of PCa, we meticulously collected and registered a total of 80 published signatures (Additional file 2: Table S5). These signatures encompassed various biological processes, including inflammation, cell death, fatty acid metabolism, glucose metabolism, and others. Remarkably, ECMGPS exhibited the highest C-index among the remaining cohorts (TCGA, MSKCC, GSE70768, and GSE70769), except for ranking third in the DKFZ cohort (Fig. 7A–E). These findings underscore the exceptional predictive performance of ECMGPS and its potential for extrapolation.

### Predictive value of ECMGPS in therapy

**Fig. 8:**
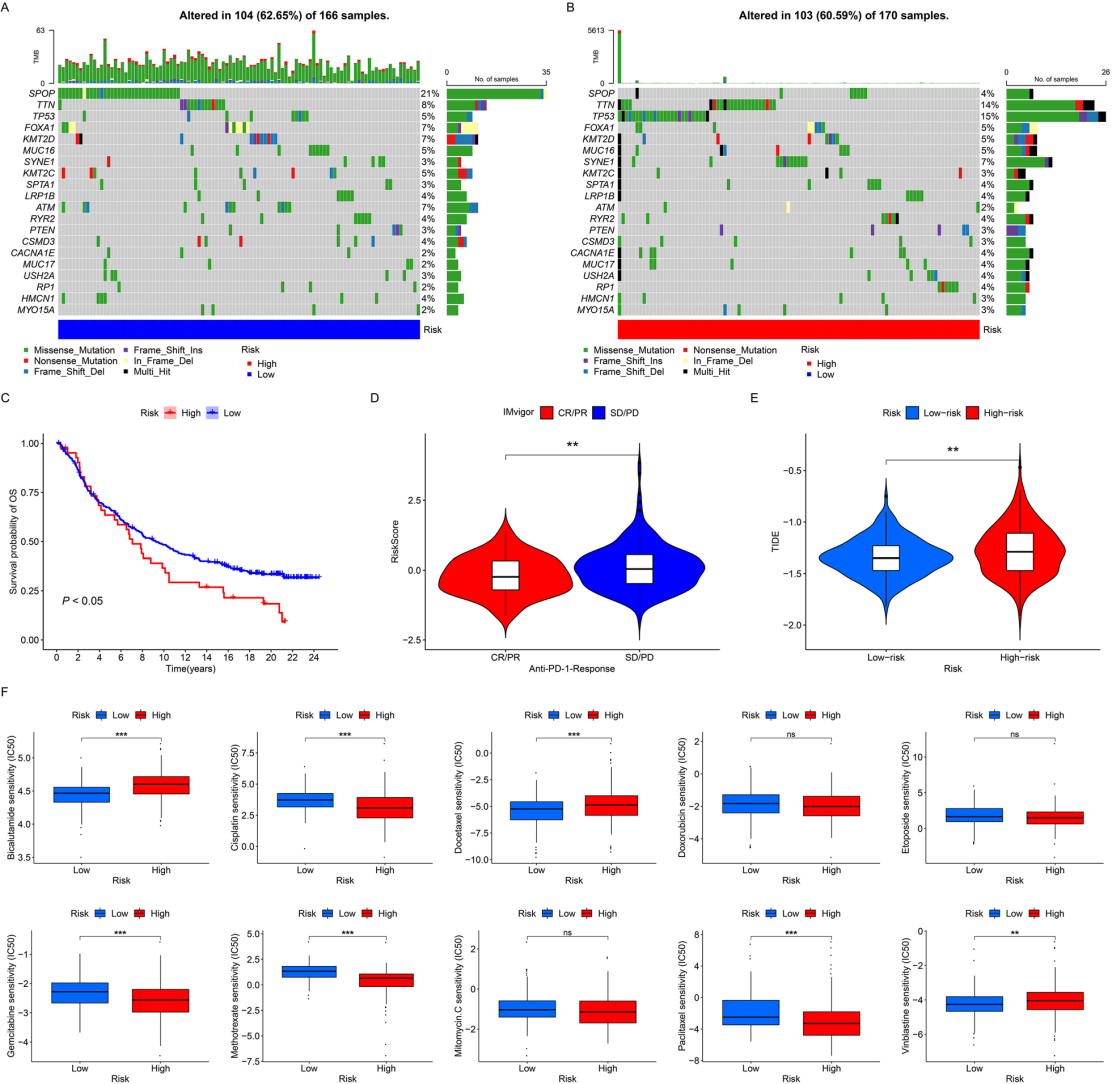
Predictive value of ECMGPS in therapy. **A** Waterfall map displaying somatic mutations in the low-risk group. **B** Waterfall map displaying somatic mutations in the high-risk group. **C** Kaplan–Meier curves for OS between patients with different risk scores of ECMGPS in the IMvigor cohort. **D** Violin plot comparing the risk scores of ECMGPS between patients with different immunotherapy responses in the IMvigor cohort. **E** Violin plot comparing tumor immune dysfunction and exclusion (TIDE) scores between the low- and high-risk groups. **F** Box plots comparing the sensitivity (IC50) of 10 anti-PCa drugs between the low- and high-risk groups. **P < 0.01, ***P < 0.001. *ns* not significant

Based on our previous evidence demonstrating that the high immune patterns promoted aggressive and poor prognosis in ECMGs in PCa, we sought to explore the potential of the ECMGPS in predicting the response to immunotherapy and medication, thereby maximizing its clinical applicability. Initially, we observed a higher mutation frequency in the low-risk group in contrast to the high-risk group, with SPOP mutations detected in 21% of the samples (Fig. 8A, B). Moreover, by constructing the ECMGPS directly using the IMvigor cohort, a dataset focused on immunotherapy, we identified prolonged survival in low-risk patients with urothelial carcinoma (*P* < 0.05) (Fig. 8C). Consistently, patients with lower risk scores demonstrated a higher likelihood of responding to anti-PD-L1 immunotherapy (Fig. 8D). Subsequently, the TIDE algorithm validated that higher risk scores were associated with an increased probability of immune evasion (Fig. 8E). These collective findings strongly suggest that the low-risk group identified by ECMGPS is more likely to derive benefits from immunotherapy.

Lastly, to explore the potential application of ECMGPS in precise and personalized drug selection for PCa, we compared the sensitivity differences to ten commonly used chemotherapeutic and targeted drugs based on IC50 values between the low-risk and high-risk groups. Surprisingly, we observed significant differences in sensitivity to seven commonly used anti-PCa drugs. Specifically, patients in the high-risk group exhibited higher sensitivity to bicalutamide, docetaxel, and vinblastine, while patients in the low-risk group showed greater sensitivity to cisplatin, gemcitabine, methotrexate, and paclitaxel (Fig. 8F). Overall, these findings suggest that ECMGPS can aid clinicians in selecting personalized drugs and devising tailored treatment strategies for individual patients.

### TMED3 promotes malignant proliferation of PCa cells

**Fig. 9:**
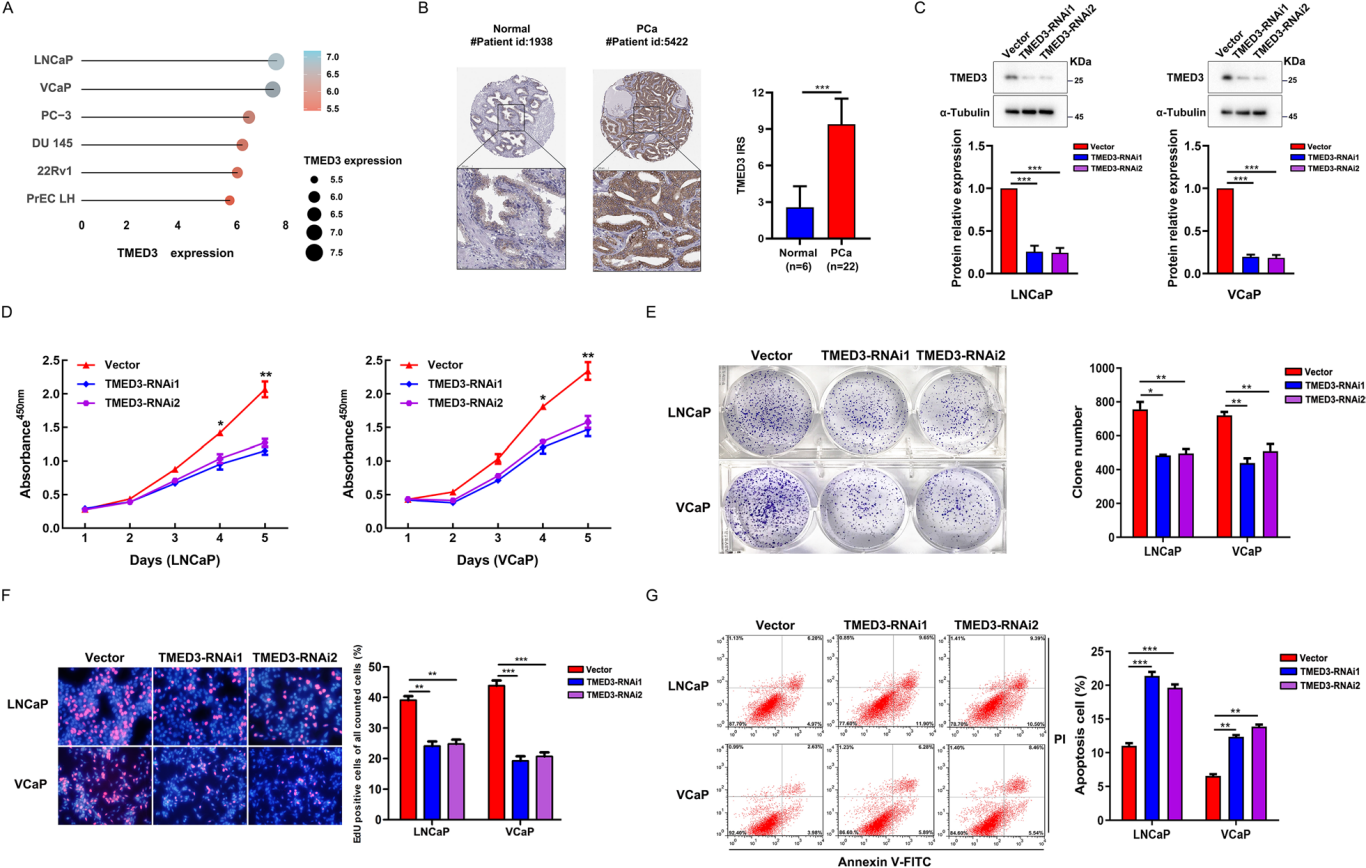
TMED3 promotes malignant proliferation of PCa cells. **A** Distribution of mRNA expression across different cell lines. **B** Distribution of protein expression in normal prostate and PCa tissues obtained from the HPA database. **C** Efficiency of TMED3 knockdown in LNCaP and VCaP cells. **D** CCK-8 assay measuring cell viability in LNCaP and VCaP cells. **E** Colony formation assay assessing the colony-forming ability of LNCaP and VCaP cells. **F** EdU assay evaluating cell proliferation in LNCaP and VCaP cells. **G** Apoptosis analysis of LNCaP and VCaP cells using flow cytometry. *P < 0.05, **P < 0.01, ***P < 0.001

PCa is a malignancy characterized by the abnormal proliferation of epithelial cells in the prostate gland of males [2]. Therefore, we postulated that the upregulated genes comprising our model hold significant research value in comprehending the malignant progression of PCa cells. Consistent with our expectations, an extensive literature review revealed that the majority of upregulated genes had been unequivocally associated with critical roles in the malignant progression and prognosis of PCa. Unfortunately, although studies have indicated that TMED3 is a biomarker that promotes malignant progression in various malignancies and exhibits heightened expression in PCa, its precise functions in PCa remain elusive. To address this knowledge gap, we utilized the CCLE dataset to acquire a gene expression matrix of PCa cell lines (Fig. 9A). This analysis confirmed substantially elevated expression levels of TMED3 in tumor tissues relative to normal tissues, particularly in the VCaP and LNCaP cell lines. Additionally, IHC results from the HPA database revealed a significant upregulation of TMED3 protein levels in PCa (Fig. 9B).

To delve further into the impact of TMED3 on PCa cell proliferation, we established TMED3 knockdown models using VCaP and LNCaP cells (Fig. 9C). The results from CCK-8, colony formation, and EdU assays collectively demonstrated a significant reduction in cell viability, colony-forming ability, and proliferative capacity following TMED3 knockdown in PCa cells (Fig. 9D–F). Moreover, decreased TMED3 expression led to a notable increase in the apoptotic rate, exhibiting a more pronounced effect compared to the control group (Fig. 9G). Taken together, these findings provide compelling evidence that TMED3 actively promotes the malignant progression of PCa cells.

## Discussion

As primary components of natural defense mechanisms and immune system sentinels, epithelial cells are critical in human tissue [32, 33]. They translate information from microenvironments to the immune system, facilitating effective stress responses [34]. Immunotherapy has emerged as a promising approach for the treatment of drug-resistant tumors, revolutionizing the management of various solid malignancies [35]. Given the unique immunostimulatory characteristics of PCa, there is growing interest in exploring immunotherapy as a potential treatment option [36–38]. However, despite the success of drugs such as sipuleucel-T and pembrolizumab, several other immunotherapeutic agents have shown unsatisfactory results in clinical trials due to the lack of personalized evaluation methods and optimal timing for treatment [39, 40]. To address this limitation, our study is the first to investigate the relationship between epithelial cell-related gene profiles, prognosis, recurrence, and therapeutic response in PCa.

In this study, we performed systematic scRNA-seq analysis to identify 543 ECMGs. Subsequently, our consensus cluster analysis of ECMGs in PCa patients from the TCGA cohort indicated that subgroups with a strong immunophenotype had worse prognoses and higher malignancy. Contrary to the conventional understanding, this observation can be reasonably attributed to the immunosuppressive role of Treg cells or the quiescent or exhausted state of memory T cells [41, 42]. The exhaustion of memory T cells or the presence of Treg cells contributes to immune evasion by tumor cells and resistance to immune checkpoint blockade therapy [43, 44]. In addition, the high expression levels of LAG-3, VISTA, TIM-3, and TIGIT in PCa patients have been confirmed to be associated with poor prognosis and closely linked to immunotherapy resistance [45], consistent with our study results.

In the era of precision medicine, relying solely on the TNM staging system, PSA levels, and other indicators is insufficient for evaluating the prognosis of PCa patients and determining the optimal timing for anti-PCa drug administration [46]. Existing PCa prognosis models often rely on individual preferences for selecting modeling algorithms or lack validation using multiple datasets, leading to poor performance or overfitting of the model [47]. To address these limitations, we collected a comprehensive set of 10 widely used machine learning algorithms and constructed 101 models. After careful evaluation, we identified the CoxBoost and SuperPC combination as the best-performing model for PCa prognosis. The resulting ECMGPS significantly reduced the dimensionality of variables, simplifying the model and enhancing its generalizability. ECMGPS demonstrated excellent prediction performance across multiple datasets and outperformed 80 published signatures in predicting BCR in PCa patients. Importantly, ECMGPS emerged as the only independent and prognostic indicator across all cohorts, surpassing clinical indicators such as PSA and the TNM staging system. Therefore, our signature offers value as an adjunct to evaluate PCa prognosis and stratify patients as high or low-risk of aggressive disease. Additionally, ECMGPS showed accurate stratification of patients with bladder cancer and renal clear cell cancer, both of which originate from malignant proliferation of epithelial cells in the urinary system, suggesting its wide application prospects.

Building upon the close association between ECMGs, prognosis, and immunity, we additionally observed a higher mutation frequency in the low-risk group. Conversely, TP53 and TTN mutations were more prevalent in the high-risk group. Previous studies have suggested that mutations in TP53 and TTN contribute to the proliferation and metastasis of PCa [48, 49], which substantiates the poorer prognosis observed in the high-risk group. These findings provide valuable insights into the potential use of ECMGPS in immunotherapy. As expected, both TIDE analysis and examination of the IMvigor cohort, along with drug sensitivity assessments, consistently indicated that ECMGPS could serve as a reference for the early identification of PCa patients who exhibit sensitivity to immunotherapy and drugs. In this way, our work could pave the way for biomarker-directed, personalized therapeutic regimens that combine the optimal drugs and treatment intensities for each patient.

In addition, our study revealed a significant biological correlation among the model genes. Moreover, through an extensive review of the literature on these genes in PCa, we found compelling evidence confirming the close association of most genes, particularly the upregulated genes, with malignant proliferation, poor prognosis, and the tumor immune microenvironment of PCa [50–56]. Our previous basic research on CDKN1A also supports this finding [30]. These results strongly support the substantial impact of ECMGPS on prognosis, the tumor immune microenvironment, and its role in the clinical management and precise treatment of PCa. It is noteworthy that our study delved into the potential physiological functions of TMED3, a member of the TMED family implicated in protein vesicle transport and innate immune signal transmission [57], in the onset and progression of PCa. This underscores the viability of TMED3 as a therapeutic target for PCa. We intend to pursue further research to elucidate its molecular mechanism in upcoming studies.

Although the ECMGPS is clinically significant in predicting the prognosis of PCa, there are several limitations to acknowledge in this study. Firstly, all the included datasets were derived from retrospective studies in public databases, and future verification of the ECMGS should be conducted in prospective multi-center studies. Additionally, some clinical and molecular features in public databases are insufficient, which may conceal potential associations between ECMGS and certain variables. Finally, to validate the predictive role of ECMGPS in immunotherapy response, additional immunotherapy cohorts comprising PCa patients are of utmost importance and urgently needed.

## Conclusions

Our integrated prognostic signature represents a significant advancement over previous models, as it meticulously combines machine learning algorithms with multiple independent validation cohorts. The remarkable performance and applicability of our signature across diverse datasets underscore its strength and dependability as a clinical tool. Once clinically validated, ECMGPS has the potential to enhance decision-making by identifying patients who are likely to face aggressive disease and poorer outcomes with standard treatments. Furthermore, our signature shows promise in predicting immunotherapy response, enabling a more personalized and timely application of immune-based treatments. Overall, ECMGPS provides a versatile platform that has the potential to greatly enhance risk assessment, prognosis prediction, and immunotherapy selection for the improved clinical management of PCa patients.

### Supplementary Information

**Additional file 1: Figure S1.** Consensus clustering of ECMGs. **Figure S2.** Evaluation of clinical independence and application value of ECMGPS in the MSKCC cohort. **Figure S3.** Evaluation of clinical independence and application value of ECMGPS in the GSE70768 cohort. **Figure S4.** Evaluation of clinical independence and application value of ECMGPS in the DKFZ cohort. **Figure S5.** Evaluation of clinical independence and application value of ECMGPS in the GSE70769 cohort.

**Additional file 2: Table S1.** The main clinicopathological features of PCa patients in the study. **Table S2.** The gene sets with annotation of 29 immune cells and pathways. **Table S3.** The 15 clusters with annotation in scRNA-seq. **Table S4.** The results of univariate Cox regression analysis for prognostic ECMGs. **Table S5.** A total of 80 published signatures were retrieved from the literatures.

## Data Availability

The datasets generated and analyzed during the current study are available in TCGA (https://portal.gdc.cancer.gov/), GEO (https://www.ncbi.nlm.nih/), MSKCC (https://www.mskcc.org/), DKFZ (https://www.cbioportal.org/study/summary?id=prostate_dkfz_2018/), IMvigor 210 (https://research-pub.gene.com/IMvigor210CoreBiologies/), and CCLE (https://portals.broadinstitute.org/ccle/about/). Essential scripts for our study are available on the Github website (https://github.com/ZooWA/ECMGPS). All data and materials generated and/or analyzed during the current study are available from online repositories or the corresponding author on reasonable request.

## Abbreviations

PCa: Prostate cancer
ECMGs: Epithelial cell marker genes
ECMGPS: Epithelial cell marker gene prognostic signature
BCR: Biochemical recurrence
scRNA-seq: Single-cell RNA sequencing
TCGA: The Cancer Genome Atlas
GEO: Gene Expression Omnibus
MSKCC: Memorial Sloan Kettering Cancer Center
DKFZ: Deutsches Krebsforschungszentrum
CCLE: Cancer Cell Line Encyclopedia
PCA: Principal component analysis
t-SNE: T-distributed stochastic neighbor embedding
CDF: Cumulative distribution function
bRFS: Biochemical recurrence-free survival
CNV: Copy number variation
GSVA: Gene set variation analysis
ssGSEA: Single-sample gene set enrichment analysis
Enet: Elastic network
survival-SVM: Survival support vector machine
plsRcox: Partial least squares regression for Cox
RSF: Random survival forest
SuperPC: Supervised principal components
GBM: Generalized boosted regression modeling
C-index: Concordance index
ROC: Receiver operating characteristic
TIDE: Tumor immune dysfunction and exclusion
IHC: Immunohistochemistry
TMED3: Transmembrane p24 trafficking protein 3
HPA: Human Protein Atlas
CCK-8: Cell counting kit-8
AUCs: Areas under the ROC curve
OS: Overall survival
