# Supplementary material for: Integrated machine learning identifies epithelial cell marker genes for improving outcomes and immunotherapy in prostate cancer
Source: J Transl Med. 2023 Nov 4;21:782. doi: 10.1186/s12967-023-04633-2 (PMC10625713; doi:10.1186/s12967-023-04633-2)
Supplement: Supplementary file 1 — Additional file 1: Figure S1. Consensus clustering of ECMGs. Figure S2. Evaluation of clinical independence and application value of ECMGPS in the MSKCC cohort. Figure S3. Evaluation of clinical independence and application value of ECMGPS in the GSE70768 cohort. Figure S4. Evaluation of clinical independence and application value of ECMGPS in the DKFZ cohort. Figure S5. Evaluation of clinical independence and application value of ECMGPS in the GSE70769 cohort. [file 12967_2023_4633_MOESM1_ESM.docx]

***Additional Figures***

**
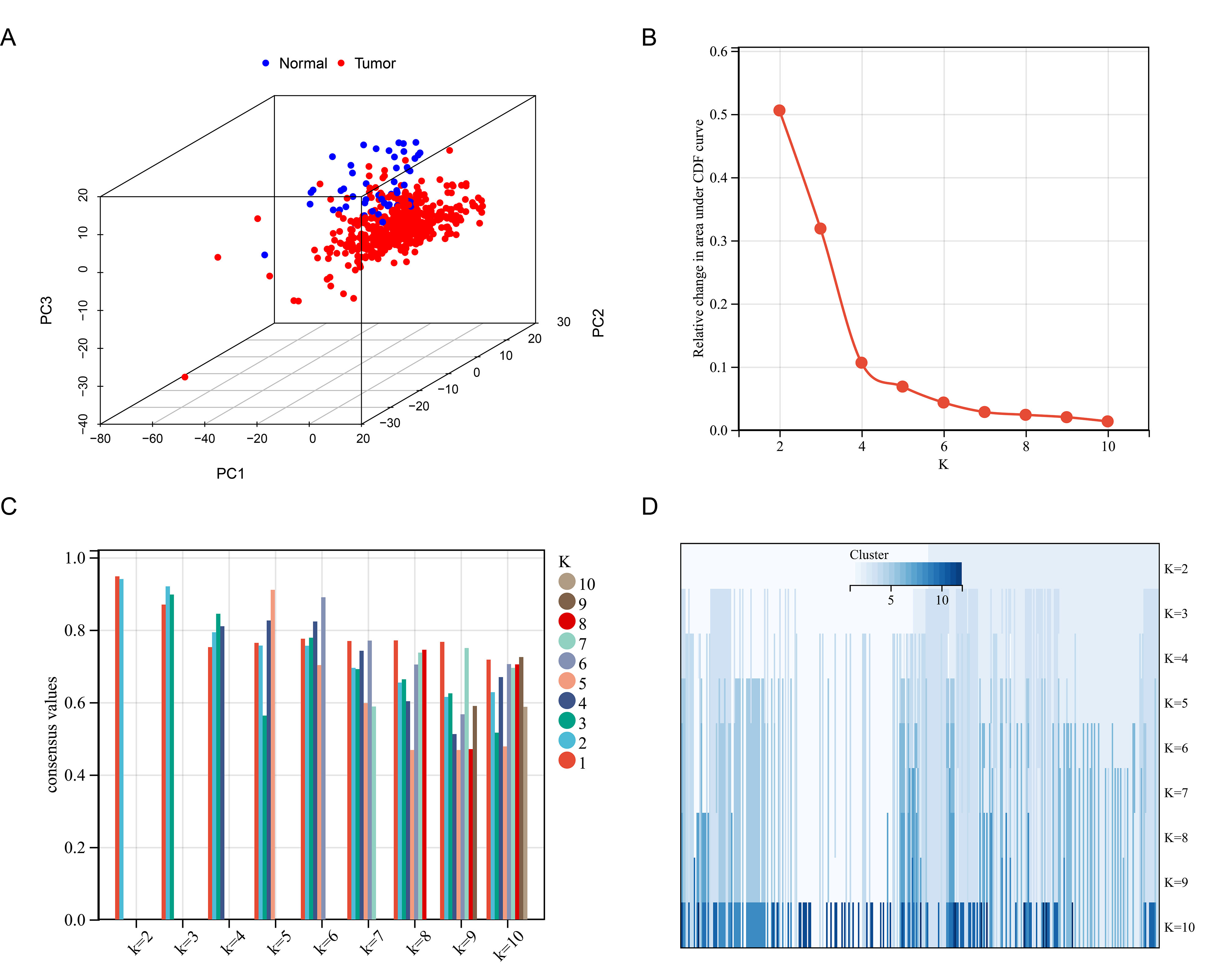
**

**Figure S1. Consensus clustering of ECMGs. (A)** 3D-PCA plot showing the distribution between PCa and normal samples in the TCGA database. **(B)** CDF curves with different values of k. **(C)** Relative change in the area under the CDF curves with different values of k. **(D)** Sample distribution with different values of k.


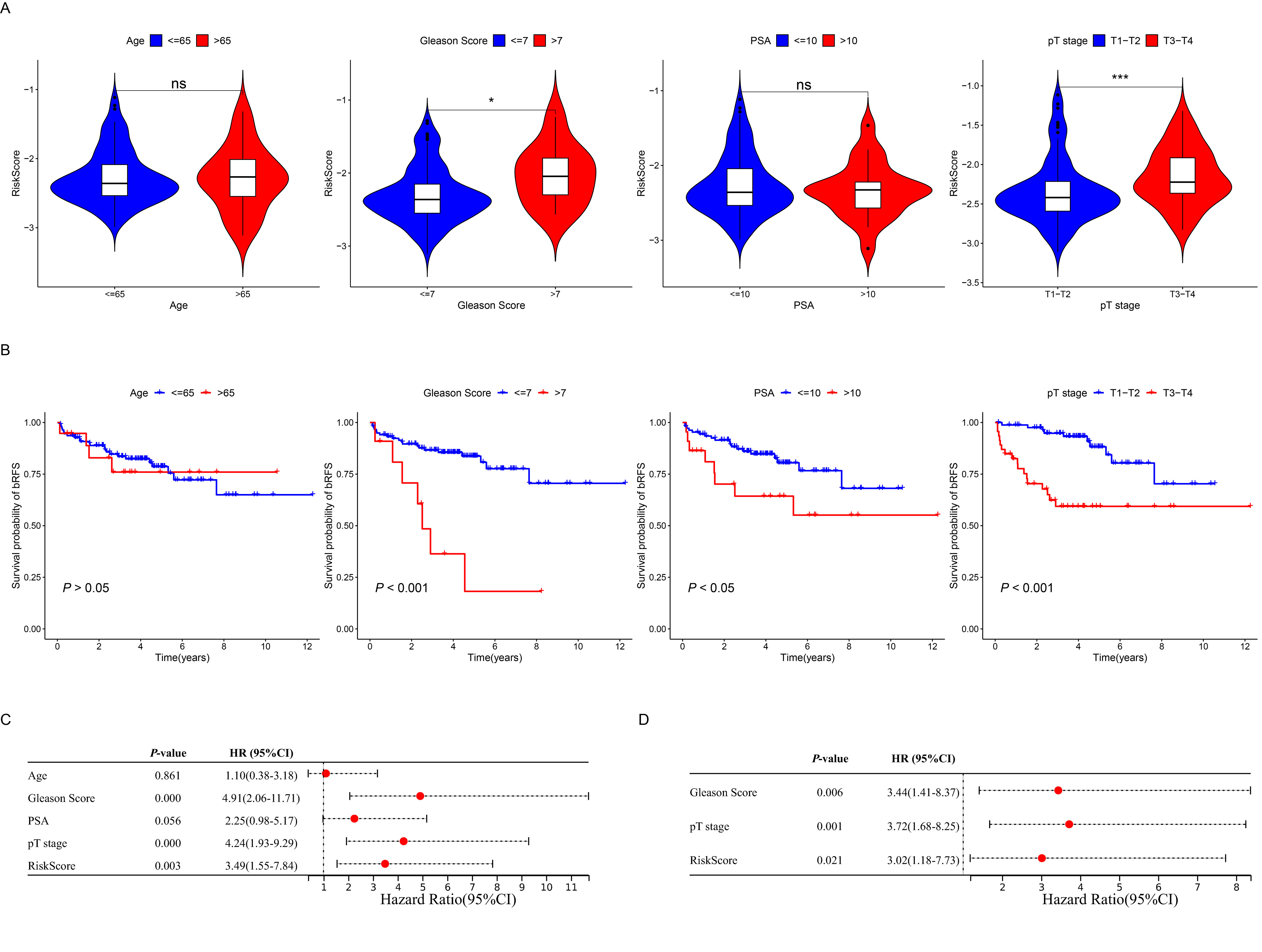


**Figure S2. Evaluation of** **clinical independence and application value of ECMGPS in the MSKCC cohort. (A)** Violin plots comparing the risk scores between different subgroups stratified by clinicopathological features. **(B)** Kaplan–Meier curves for bRFS stratified by clinicopathological features. **(C)** Univariate Cox regression analysis of ECMGPS in relation to bRFS. **(D)** Multivariate Cox regression analysis of ECMGPS in relation to bRFS. **P* <0.05, *** *P* < 0.001.


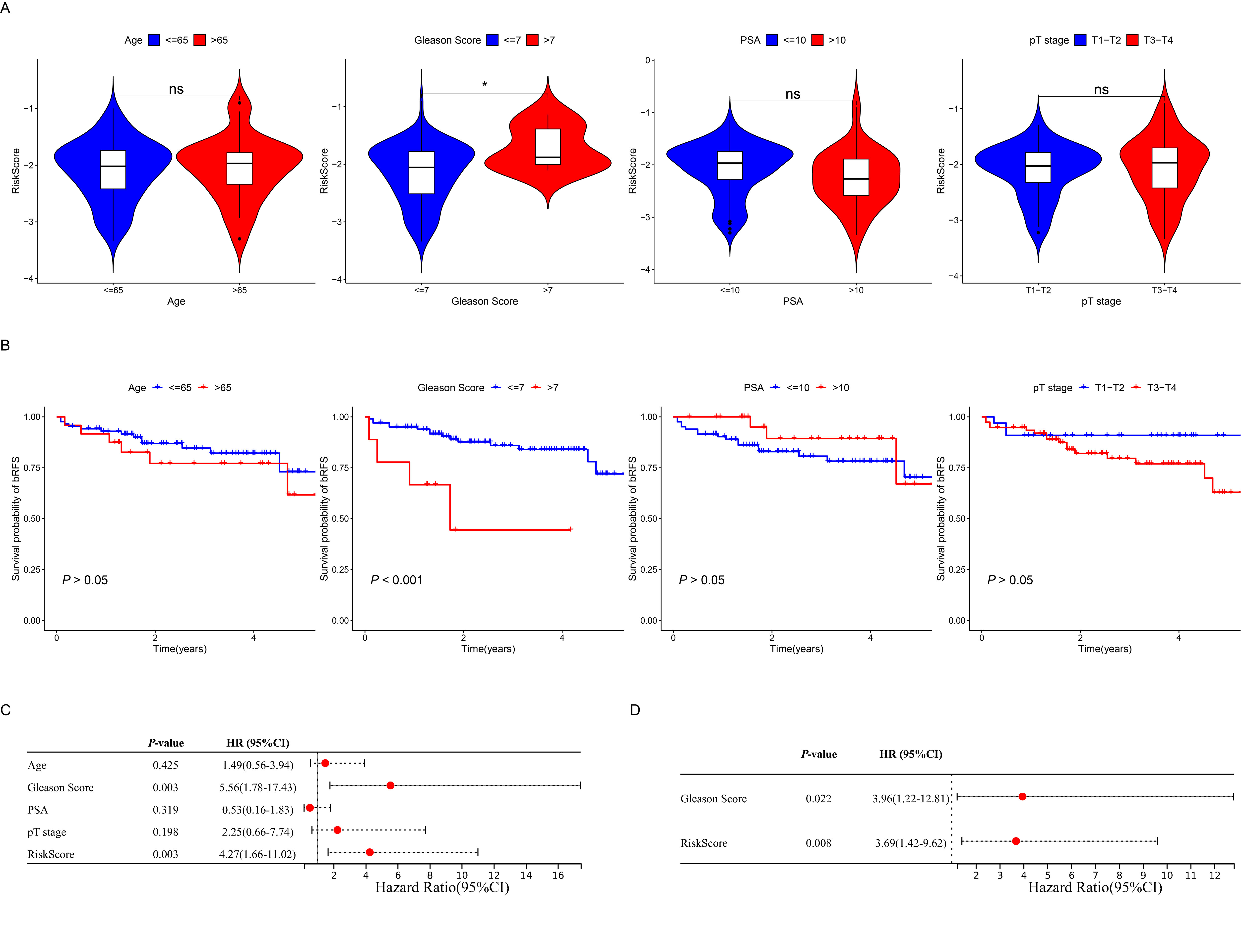


**Figure S3. Evaluation of** **clinical independence and application value of ECMGPS in the GSE70768 cohort. (A)** Violin plots comparing the risk scores between different subgroups stratified by clinicopathological features. **(B)** Kaplan–Meier curves for bRFS stratified by clinicopathological features. **(C)** Univariate Cox regression analysis of ECMGPS in relation to bRFS. **(D)** Multivariate Cox regression analysis of ECMGPS in relation to bRFS. **P* <0.05.


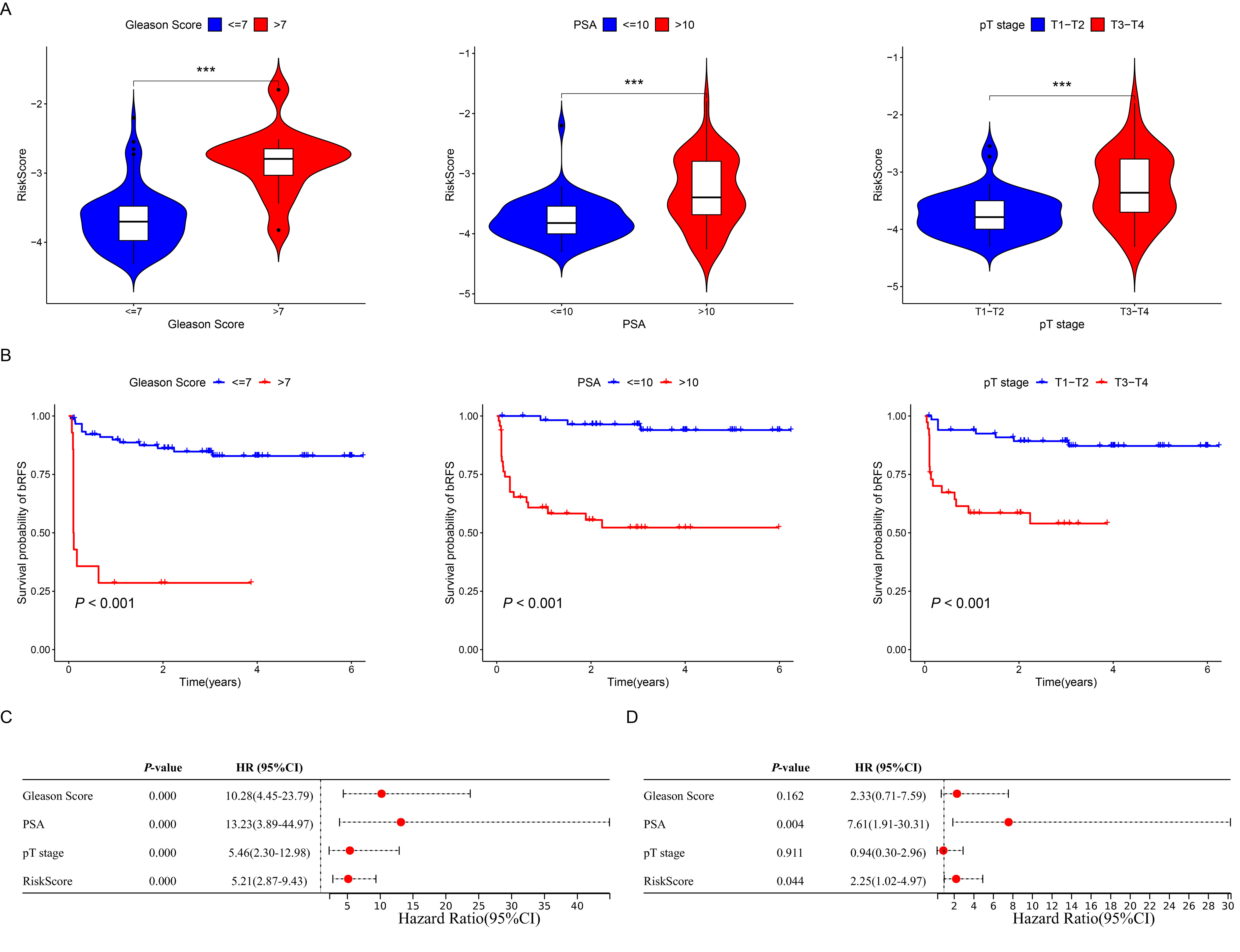


**Figure S4. Evaluation of** **clinical independence and application value of ECMGPS in the DKFZ cohort. (A)** Violin plots comparing the risk scores between different subgroups stratified by clinicopathological features. **(B)** Kaplan–Meier curves for bRFS stratified by clinicopathological features. **(C)** Univariate Cox regression analysis of ECMGPS in relation to bRFS. **(D)** Multivariate Cox regression analysis of ECMGPS in relation to bRFS. *** *P* < 0.001.


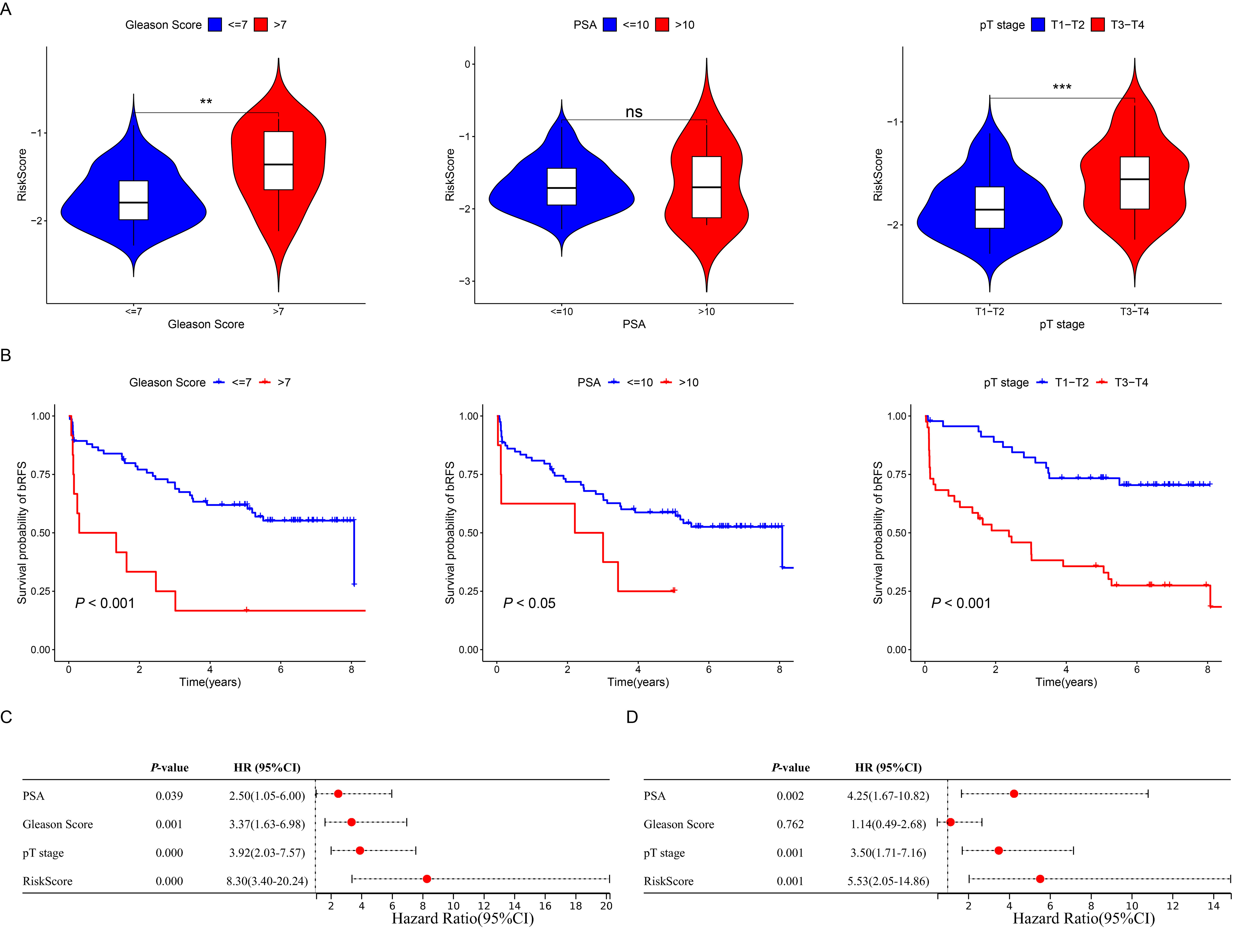


**Figure S5. Evaluation of** **clinical independence and application value of ECMGPS in the GSE70769 cohort. (A)** Violin plots comparing the risk scores between different subgroups stratified by clinicopathological features. **(B)** Kaplan–Meier curves for bRFS stratified by clinicopathological features. **(C)** Univariate Cox regression analysis of ECMGPS in relation to bRFS. **(D)** Multivariate Cox regression analysis of ECMGPS in relation to bRFS. ** *P* < 0.01, *** *P* < 0.001.
