# Supplementary material for: Integrated machine learning identifies epithelial cell marker genes for improving outcomes and immunotherapy in prostate cancer
Source: J Transl Med. 2023 Nov 4;21:782. doi: 10.1186/s12967-023-04633-2 (PMC10625713; doi:10.1186/s12967-023-04633-2)
Supplement: Supplementary file 2 — Additional file 2: Table S1. The main clinicopathological features of PCa patients in the study. Table S2. The gene sets with annotation of 29 immune cells and pathways. Table S3. The 15 clusters with annotation in scRNA-seq. Table S4. The results of univariate Cox regression analysis for prognostic ECMGs. Table S5. A total of 80 published signatures were retrieved from the literatures. [file 12967_2023_4633_MOESM2_ESM.docx]

***Supplementary Tables***

**Table S1. The main clinicopathological features of PCa patients in the study.**

| **Variables** | **TCGA** | **MSKCC** | **GSE70768** | **DKFZ** | **GSE70769** |
| --- | --- | --- | --- | --- | --- |
| **Total** | 346 | 134 | 110 | 105 | 92 |
| **Age (years)** |  |  |  |  |  |
| ≤65 | 247 | 113 | 86 | - | - |
| >65 | 99 | 21 | 24 | - | - |
| **Gleason Score** |  |  |  |  |  |
| ≤7 | 190 | 123 | 101 | 91 | 75 |
| >7 | 156 | 11 | 9 | 14 | 12 |
| NA | - | - | - | - | 5 |
| **PSA (ng/mL)** |  |  |  |  |  |
| ≤10 | 335 | 108 | 83 | 58 | 79 |
| >10 | 11 | 22 | 27 | 47 | 8 |
| NA | - | 4 | - | - | 5 |
| **Status** |  |  |  |  |  |
| BCR | 37 | 31 | 19 | 24 | 45 |
| Non-BCR | 309 | 103 | 91 | 81 | 47 |
| **Pathological T stage** |  |  |  |  |  |
| T1-2 | 121 | 85 | 33 | 68 | 46 |
| T3-4 | 225 | 49 | 77 | 37 | 41 |
| NA | - | - | - | - | 5 |
| **Pathological N stage** |  |  |  |  |  |
| N0 | 287 | - | - | - | - |
| N1 | 59 | - | - | - | - |

PSA, prostate-specific antigen; BCR, biochemical recurrence

**Table S2. The gene sets with annotation of 29 immune cells and pathways.**

| **Terms** | **Genes** |
| --- | --- |
| **aDCs** | CD83, LAMP3, CCL1 |
| **APC_co_inhibition** | C10orf54, CD274, LGALS9, PDCD1LG2, PVRL3 |
| **APC_co_stimulation** | CD40, CD58, CD70, ICOSLG, SLAMF1, TNFSF14, TNFSF15, TNFSF18, TNFSF4, TNFSF8, TNFSF9 |
| **B_cells** | BACH2, BANK1, BLK, BTLA, CD79A, CD79B, FCRL1, FCRL3, HVCN1, RALGPS2 |
| **CCR** | CCL16, TPO, TGFBR2, CXCL2, CCL14, TGFBR3, IL11RA, CCL11, IL4I1, IL33, CXCL12, CXCL10, BMPER, BMP8A, CXCL11, IL21R, IL17B, TNFRSF9, ILF2, CX3CR1, CCR8, TNFSF12, CSF3, TNFSF4, BMP3, CX3CL1, BMP5, CXCR2, TNFRSF10D, BMP2, CXCL14, CCL28, CXCL3, BMP6, CCL21, CXCL9, CCL23, IL6, TNFRSF18, IL17RD, IL17D, IL27, CCL7, IL1R1, CXCR4, CXCR2P1, TGFB1I1, IFNGR1, IL9R, IL1RAPL1, IL11, CSF1, IL20RA, IL25, TNFRSF4, IL18, ILF3, CCL20, TNFRSF12A, IL6ST, CXCL13, IL12B, TNFRSF8, IL6R, BMPR2, IFNE, IL1RAPL2, IL3RA, BMP4, CCL24, TNFSF13B, CCR4, IL2RA, IL32, TNFRSF10C, IL22RA1, BMPR1A, CXCR5, CXCR3, IFNA8, IL17REL, IFNB1, IFNAR1, TNFRSF1B, CCL17, IFNL1, IL16, IL1RL1, ILK, CCL25, ILDR2, CXCR1, IL36RN, IL34, TGFB1, IFNG, IL19, ILKAP, BMP2K, CCR10, ILDR1, EPO, CCR7, IL17C, IL23A, CCR5, IL7, EPOR, CCL13, IL2RG, IL31RA, TNFAIP6, IFNL2, BMP1, IL12RB1, TNFAIP8, IL4R, TNFRSF6B, TNFAIP8L1, TNFRSF10B, IFNL3, CCL5, CXCL6, CXCL1, CCR3, TNFSF11, CSF1R, IL21, IL1RAP, IL12RB2, CCL1, IL17RA, CCR1, IL1RN, TNFRSF11B, TNFRSF14, IL13, IL2RB, BMP8B, CCL2, IL24, IL18RAP, TGFBI, TNFSF10, TNFRSF11A, CXCL5, IL5RA, TNFSF9, IL1RL2, TNFRSF13C, IL36G, IL15RA, TNFRSF21, CXCL8, IL22RA2, TNFAIP8L2, IL18R1, IFNLR1, CXCR6, CCL3L3, TNFRSF1A, IL17RE, IFNGR2, IL17RC, TNFAIP8L3, ILVBL, TGFBRAP1, CCL4L1, CSF2RA, CCRN4L, CCL26, TNFAIP1, CCRL2, IFNA10, TNFRSF17, IFNA13, IL20, IL18BP, CCL3L1, TNFSF12-TNFSF13, IL5, IL23R, IL26, TNF, TGFA, CSF2, IL1F10, CXCL17, TNFSF13, IFNA4, IL37, IL12A, IL7R, IFNA1, IL1A, IL4, IL2, CCL22, CSF3R, IL10, IFNK, TGFB2, IL1R2, IL1B, IL17F, IL27RA, IL15, TNFSF8, IL36B, XCL1, CXCL16, TNFRSF19, IL3, CCL3, IFNA2, BMPR1B, IFNA21, TNFSF18, CCL8, IL17RB, TNFRSF25, IL22, IL10RB, IFNAR2, CCL18, IFNA16, CSF2RB, IL36A, TNFAIP3, IL13RA2, IL13RA1, CCR9, TNFRSF10A, IFNA7, IFNW1, XCL2, TNFSF14, CCR2, BMP15, BMP10, CCL15-CCL14, TGFBR1, IFNA5, BMP7, IFNA14, IL20RB, IL10RA, IFNA17, CCR6, TGFB3, CCL15, CCL4, CCL27, TNFRSF13B, TNFAIP2, IL31, IL17A, TNFSF15, CCL19, IFNA6, IL9 |
| **CD8+_T_cells** | CD8A |
| **Check-point** | IDO1, LAG3, CTLA4, TNFRSF9, ICOS, CD80, PDCD1LG2, TIGIT, CD70, TNFSF9, ICOSLG, KIR3DL1, CD86, PDCD1, LAIR1, TNFRSF8, TNFSF15, TNFRSF14, IDO2, CD276, CD40, TNFRSF4, TNFSF14, HHLA2, CD244, CD274, HAVCR2, CD27, BTLA, LGALS9, TMIGD2, CD28, CD48, TNFRSF25, CD40LG, ADORA2A, VTCN1, CD160, CD44, TNFSF18, TNFRSF18, BTNL2, C10orf54, CD200R1, TNFSF4, CD200, NRP1 |
| **Cytolytic_activity** | PRF1, GZMA |
| **DCs** | CCL17, CCL22, CD209, CCL13 |
| **HLA** | HLA-E, HLA-DPB2, HLA-C, HLA-J, HLA-DQB1, HLA-DQB2, HLA-DQA2, HLA-DQA1, HLA-A, HLA-DMA, HLA-DOB, HLA-DRB1, HLA-H, HLA-B, HLA-DRB5, HLA-DOA, HLA-DPB1, HLA-DRA, HLA-DRB6, HLA-L, HLA-F, HLA-G, HLA-DMB, HLA-DPA1 |
| **iDCs** | CD1A, CD1E |
| **Inflammation-promoting** | CCL5, CD19, CD8B, CXCL10, CXCL13, CXCL9, GNLY, GZMB, IFNG, IL12A, IL12B, IRF1, PRF1, STAT1, TBX21 |
| **Macrophages** | C11orf45, CD68, CLEC5A, CYBB, FUCA1, GPNMB, HS3ST2, LGMN, MMP9, TM4SF19 |
| **Mast_cells** | CMA1, MS4A2, TPSAB1 |
| **MHC_class_I** | B2M, HLA-A, TAP1 |
| **Neutrophils** | EVI2B, HSD17B11, KDM6B, MEGF9, MNDA, NLRP12, PADI4, SELL, TRANK1, VNN3 |
| **NK_cells** | KLRC1, KLRF1 |
| **Parainflammation** | CXCL10, PLAT, CCND1, LGMN, PLAUR, AIM2, MMP7, ICAM1, MX2, CXCL9, ANXA1, TLR2, PLA2G2D, ITGA2, MX1, HMOX1, CD276, TIRAP, IL33, PTGES, TNFRSF12A, SCARB1, CD14, BLNK, IFIT3, RETNLB, IFIT2, ISG15, OAS2, REL, OAS3, CD44, PPARG, BST2, OAS1, NOX1, PLA2G2A, IFIT1, IFITM3, IL1RN |
| **pDCs** | CLEC4C, CXCR3, GZMB, IL3RA, IRF7, IRF8, LILRA4, PHEX, PLD4, PTCRA |
| **T_cell_co-inhibition** | BTLA, C10orf54, CD160, CD244, CD274, CTLA4, HAVCR2, LAG3, LAIR1, TIGIT |
| **T_cell_co-stimulation** | CD2, CD226, CD27, CD28, CD40LG, ICOS, SLAMF1, TNFRSF18, TNFRSF25, TNFRSF4, TNFRSF8, TNFRSF9, TNFSF14 |
| **T_helper_cells** | CD4 |
| **Tfh** | PDCD1, CXCL13, CXCR5 |
| **Th1_cells** | IFNG, TBX21, CTLA4, STAT4, CD38, IL12RB2, LTA, CSF2 |
| **Th2_cells** | PMCH, LAIR2, SMAD2, CXCR6, GATA3, IL26 |
| **TIL** | ITM2C, CD38, THEMIS2, GLYR1, ICOS, F5, TIGIT, KLRD1, IRF4, PRKCQ, FCRL5, SIRPG, LPXN, IL2RG, CCL5, LCK, TRAF3IP3, CD86, MAL, LILRB1, DOK2, CD6, PAG1, LAX1, PLEK, PIK3CD, SLAMF1, XCL1, GPR171, XCL2, TBX21, CD2, CD53, KLHL6, SLAMF6, CD40, SIT1, TNFRSF4, CD79A, CD247, LCP2, CD3D, CD27, SH2D1A, FYB, ARHGAP30, ACAP1, CST7, CD3G, IL2RB, CD3E, FCRL3, CORO1A, ITK, TCL1A, CYBB, CSF2RB, IKZF1, NCF4, DOCK2, CCR2, PTPRC, PLAC8, NCKAP1L, IL7R, 6-Sep, CD28, STAT4, CD8A, LY9, CD48, HCST, PTPRCAP, SASH3, ARHGAP25, LAT, TRAT1, IL10RA, PAX5, CCR7, DOCK11, PARVG, SPNS1, CD52, HCLS1, ARHGAP9, GIMAP6, PRKCB, MS4A1, GPR18, TBC1D10C, GVINP1, P2RY8, EVI2B, VAMP5, KLRK1, SELL, MPEG1, MS4A6A, ARHGAP15, MFNG, GZMK, SELPLG, TARP, GIMAP7, FAM65B, INPP5D, ITGA4, MZB1, GPSM3, STK10, CLEC2D, IL16, NLRC3, GIMAP5, GIMAP4, IFFO1, CFH, PVRIG, CFHR1 |
| **Treg** | IL12RB2, TMPRSS6, CTSC, LAPTM4B, TFRC, RNF145, NETO2, ADAT2, CHST2, CTLA4, NFE2L3, LIMA1, IL1R2, ICOS, HSDL2, HTATIP2, FKBP1A, TIGIT, CCR8, LTA, SLC35F2, IL21R, AHCYL1, SOCS2, ETV7, BCL2L1, RRAGB, ACSL4, CHRNA6, BATF, LAX1, ADPRH, TNFRSF4, ANKRD10, CD274, CASP1, LY75, NPTN, SSTR3, GRSF1, CSF2RB, TMEM184C, NDFIP2, ZBTB38, ERI1, TRAF3, NAB1, HS3ST3B1, LAYN, JAK1, VDR, LEPROT, GCNT1, PTPRJ, IKZF2, CSF1, ENTPD1, TNFRSF18, METTL7A, KSR1, SSH1, CADM1, IL1R1, ACP5, CHST7, THADA, CD177, NFAT5, ZNF282, MAGEH1 |
| **Type_I_IFN_Reponse** | DDX4, IFIT1, IFIT2, IFIT3, IRF7, ISG20, MX1, MX2, RSAD2, TNFSF10 |
| **Type_II_IFN_Reponse** | GPR146, SELP, AHR |

**Table S3. The 15 clusters with annotation in scRNA-seq.**

| **Cluster**  **(ID)** | **Cluster**  **(Labels)** | **Marker Genes** |
| --- | --- | --- |
| 1, 2, 4, 5, 7, 8, 10 | Epithelial_cells | ACPP, KLK3, MSMB, KLK2, NEFH, AZGP1, RDH11, NKX3-1, TMPRSS2, PLA2G2A, TACSTD2, NPY, CLDN4, STEAP2, TSPAN1, SLC45A3, GOLM1, DHCR24, DSP, FOLH1, SLC44A4, MME, FXYD3, GDF15, HOXB13, RAB3B, DHRS7, KLK4, CKB, PTPRF, KIAA1244, CPE, PMEPA1, AGR2, PPAP2A, CDH1, SLC30A4, KRT18, SEC11C, DPP4, KIAA1324, PDLIM5, ARG2, EHF, SLC39A6, ELF3, FOXA1, SPDEF, NEDD4L, NIPAL3, MLPH, CREB3L4, TPD52, CLDN3, STEAP4, DCXR, SPOCK1, SORD, EPCAM, ALDH1A3, DSG2, NDRG1, HOMER2, ENTPD5, H2AFJ, CYB561, NUPR1, ABCC4, C1orf116, TBX3, SPINT2, IDH1, PPAPDC1B, PERP, KRT8, BMPR1B, P4HB, TSPAN8, SLC12A2, PPP3CA, H1F0, PGM3, BCAS1, KIF5C, SLC2A12, CANT1, PART1, ERBB3, RBM47, TMEM141, HIST1H2AC, ADI1, STEAP1, MPC2, CD9, FLNB, GRHL2, LRIG1, ZG16B, HDLBP, TMC5, FAM213A, SFN, TMEM79, MAP7, HN1L, TSTD1, TCEA3, RASEF, APLP2, ESRP1, ANXA3, PRSS8, HLA-B, IFI16, WIPF1, TMSB10, HLA-E, ZEB2, HCLS1, LCP2, CD93, CD53, SLA, MSN, TAGAP, ARHGDIB, BTG1, TNFRSF1B, SAMHD1, FGL2, ITGB2, TYROBP, FYB, TMSB4X, CYBB, SLC2A3, CTSS, HLA-DRB5, GPR183, LAPTM5, HLA-DQA1, NR4A2, RGS2, CXCR4, TNFAIP3, VIM, PTPRC, A2M, CD74, HLA-DRB1, HLA-DPB1, SRGN, HLA-DPA1, RGS1, HLA-DRA, KCTD12, SLC4A4, CTSC, NANS, ZNF761, MBOAT2, NCAPD3, PHGDH, GALNT7, RHPN2, ANKH, WWC1, HGD, SAMSN1, ALDH3A2, ITGA4, TRPM4, CSF1R, PLEK, TRPM8, RAB27B, GPT2, ATP8B1, GCNT2, PREX1, TRPV6, FASN, GLIPR1, MAGED1, TM9SF3, SYT7, ENTPD1, MYBPC1, PLXNC1, GREB1, DOCK10, GPRIN3, NR4A3, SMIM14, CPNE4, IGF1R, TGFBI, HEBP2, HLA-DQB1, DOCK2, GSN, ANPEP, MAGED2, COBLL1, AIF1, AR, KRT19, CORO1A, LRRC26, PM20D2, ATP6V1G1, RAMP1, ATP2C1, HLA-A, ALOX15B, MAOA, ACACA, EPHX2, ARHGAP6, TMEM98, CNTNAP2, MS4A6A, PAK1IP1, ERGIC1, IRF6, DDAH1, GMFG, GFPT1, CPVL, ZNF827, SCD, MYO6, SEMA3C, FAM3B, HIST2H2BE, ARHGAP30, PKP1, APBB1IP, TRGC1, GMPR, CAB39L, XBP1, ACSL3, CD83, FAAH, AMICA1, MEF2C, DUSP2, RCSD1, NME4, HLA-DMA, FAT1, MAL2, EFNA1, COBL, CD84, SHROOM3, FAM210B, IKZF1, SC5D, EPB41L2, TMEM59, CYB5A, GLO1, CXADR, CD69, ARHGEF6, HIST1H2BD, BCL2A1, CWH43, HLA-DOA, C1QA, SLC39A7, MLLT4, PEBP1, TMEM30B, CYTIP, SERPINB9, CD4, ETS1, IL10RA, COLEC12, LGALS1, ANK3, FLNA, BAIAP2L1, TM7SF2, ANO7, FNBP1L, KIAA1217, TMED3, SYNE1, DPYSL2, SCARB2, PFKFB3, CCL4, STAP2, ANTXR2, ZKSCAN1, IL1R1, PLAUR, PDE8B, RAB31, ATP2C2, ALDH6A1, HSPH1, SEC14L2, NWD1, ALDH7A1, SLC15A2, ARSD, CORO2A, ASTN2, INADL, PPM1H, MCCC2, PDXDC1, AKAP13, SERINC5, RASSF5, CLGN, DDR1, ARFGAP3, MAP3K8, PDIA5, LSAMP, CLEC2B, PTPRN2, TMEM87A, NEO1, GNMT, PDE9A, LMAN1, TGFBR2, CRYM, IER5, ERBB2, PTPRE, CSGALNACT1, AMD1, ABAT, RCAN3, CREM, SOX9, SERPINB1, IL1B, BACE2, CYBA, SYNGR2, GUCY1A3, C10orf54, C1orf21, MCL1, ARHGEF37, DMXL1, ENDOD1, KIAA0319L, RAB3D, COPB2, ARHGAP4, CHST11, AHCY, SH3KBP1, IFT57, ZFP36L2, CELF2, PRDM1, PYGB, TXNDC16, EGFR, INPP5D, TLN1, TIMP2, DSC2, PPP1R15A, PTGER4, DBI, MBNL1, MIA3, HLA-DMB, SPARCL1, UQCRQ, TRBC2, COTL1, IVD, CCDC88A, ALCAM, JAK1, ARL4C, STK10, IGFBP7, H3F3B, PRDX6, ABHD2, NAAA, CD46, HSD17B4, ATP2B4, QKI, DNAJB1, STOM, ERLEC1, ALDH9A1, PRUNE2, REL, IFI30, SPARC, PDIA3, AFF3, CD38, HSP90AA1, CCND2, ST3GAL1, SH3BP5, ARPC1B, MYH9, GLUD1, ZNF331, PDIA4, AKNA, RASGEF1B, MLEC, HSPA1A, PNRC1, KLF5, SMAP2, ACSL4, DUSP6, TACC1, DSE, AHR, PIP4K2A, ICAM1, SPON2, FOSL2, PLEKHA2, DUSP5, REXO2, SDCBP, IL8, CDKN1A, PHLDA1, KLF6, ACTB, SYK, GNA13, DNAJB4, GADD45G, GBP2, F3, DNAJA1, GNAI2, KIAA1551, METRNL, TUBA1A, RGCC, STK4, HLA-F, DDX3X, SLC26A2, DDX3Y, RAPGEF1, S100A6, MYADM, ELF1, VEGFA, MAN1A1, NFKBIA, KLF2, FNBP1, IER3, ZFP36L1, SLC20A1, PAG1, BIRC3, FAM49B, TIMP3, OGFRL1, SH3BGRL3, PTGS2, MYO9B, C3, CHD1, DOCK8, MACF1, TPM3, CD55, CSRNP1, FTL, CAP1, ZFAND5, SOCS3, STK17B, ACTR2, IVNS1ABP, NFKBIZ, GRASP, CTSB, TUBA1B, TXNIP, ETS2, BHLHE40, DAB2, FTH1, LYN, NIN, HECA, JMJD1C, TCF4, TBC1D1, TAGLN2, CTSZ, YWHAH, PLIN2, IFITM2, FCGRT, RILPL2, SGK1, OSBPL8, ZC3HAV1, GADD45B, TSPYL2, SKIL, YPEL5, HSPA1B, CYLD, PLXDC2, TSC22D2, GLUL, EPAS1, TFRC, SMCHD1, HSPD1, PDE4B, CHMP1B, LIPA, BAG3, EIF4A3, MAFB, HBEGF, NAMPT, PSAP |
| 0 | Monocyte | HLA-DPA1, HLA-DRA, HLA-DPB1, HLA-DRB1, IL1B, CD74, HLA-DQA1, HLA-DQB1, CSF1R, GPR183, HLA-DRB5, CD83, C3, MS4A6A, FGL2, C1QA, RGS1, CYBB, C1QB, FCGR3A, TYROBP, PLEK, AIF1, CCL3, MS4A7, HLA-DQB2, SAMHD1, IL8, LYZ, HLA-DQA2, HLA-DOA, HLA-DMB, MPEG1, HLA-DMA, CD14, IFI30, CD86, OGFRL1, PLAUR, LAPTM5, CTSS, ITGB2, C1QC, CPVL, OLR1, TGFBI, RGS2, KCTD12, BCL2A1, FCGR2A, FAM26F, PLXNC1, SRGN, SIGLEC10, RNASE6, RASSF4, CD163, AXL, SYK, HCLS1, AMICA1, SERPINB9, SLCO2B1, C5AR1, VASH1, PALD1, LAIR1, IRF8, ADAM28, CD4, PTPRE, AOAH, IGSF6, CTSB, CX3CR1, LST1, TBXAS1, FCER1G, MSR1, TNFRSF1B, SH2B3, TLR2, RAB31, SLC8A1, CSF2RA, CD53, C3AR1, FTL, FYB, CD68, ADAP2, PTPRC, THEMIS2, CLEC7A, DOCK2, PLCB2, MNDA, CD300A, WDFY4, HCK, 1-Mar, HAVCR2, FGD2, NLRP3, CCDC88A, LY86, LCP2, STAB1, SCIMP, VSIG4, IER3, NR4A3, PHACTR1, CECR1, IL10RA, EPB41L2, MYO1F, NCF2, LYN, SGK1, NCKAP1L, DSE, PLAU, EMR2, ITGA4, LILRB4, CIITA, IER5, SLC15A3, PLAC8, PREX1, GLIPR1, DOCK10, ITGAX, STX11, IL18, DUSP2, PLXDC2, PTAFR, CXCR4, TFEC, COTL1, ARRB2, ARHGAP4, CD84, FPR1, PLEKHO1, RCSD1, SLA, TNF, APBB1IP, CYBA, SLC7A7, NR4A2, MEF2C, GPX1, PIK3AP1, C10orf54, SLAMF8, CD93, SAT1, C1orf162, RNASET2, ZEB2, PTGS2, RASSF5, FAM49B, ITGAM, MAFB, CXCL16, FPR3, EMILIN2, GNA13, CTSZ, CORO1A, GPRIN3, TMSB4X, TAGAP, KLHL6, SLC43A2, PRDM1, PFKFB3, MAP3K8, ALOX5AP, INPP5D, LPCAT2, CYTH4, LAT2, SMAP2, AKR1B1, FTH1, ENTPD1, PPP1R15A, ARHGDIB, G0S2, TYMP, PSAP, NFKBIA, DSP, RASSF2, NPC2, TNFAIP3, OSM, FERMT3, METRNL, ALOX5, RIN3, HSPH1, ICAM1, PPAP2A, KLK3, PPIF, CTSC, NKX3-1, KLK2, HSPA1A, PLXDC1, ALDH1A3, GRN, TMSB10, FCGRT, REL, ARL4C, STEAP2, CPE, RAB20, CCL4, TACSTD2, QKI, NDRG1, RGS19, GZF1, CHST11, ARPC1B, SPTBN1, ACPP, SERPINA1, RNF130, NFIB, TMPRSS2, TSPAN1, PAK1, GRASP, NUPR1, OTUD1, TSC22D1, BMP2K, PTPRF, APOE, ZNF331, MYO9B, AZGP1, HOXB13, CELF2, TBX3, PMEPA1, RASGEF1B, CALD1, SLC44A4, KIAA1244, MEF2A, YWHAH, CASP1, AKAP13, FSTL1, DHCR24, RDH11, CLDN4, TMEM176B, IL13RA1, NAMPT, MAN2B1, SERPINB1, SLC45A3, CXCL2, FXYD3, GOLM1, KRT18, DSTN, SLC20A1, PICALM, CDH1, RAB3B, FLNB, SH3KBP1, FILIP1L, SLAMF7, CKB, NFKBIE, TNFAIP2, MLPH, SAMSN1, UPP1, TLR4, CST3, IFNGR1, FAM49A, AR, SPOCK1, ADIRF, CREM, MME, SPDEF, KLK4, LIFR, CYB561, DOCK8, KIAA1324, ATP8B1, FOXA1, DPP4, STEAP4, PPT1, RGS10, MXD1, PERP, SOD2, MYO6, GLUL, MAGED2, NPDC1, PTRF, HSPA6, TPM1, SEC11C, NEFH, MSMB, DCXR, MAGED1, SLC39A6, TPD52, LITAF, CREB3L4, EHF, HOMER2, LIMCH1, PLSCR1, DAB2, PLIN2, LRIG1, MAOA, PDLIM5, ACTB, CDKN1A, PABPC4, GLA, SLC30A4, DHRS7, PDE4B, MGAT1, RILPL2, PCYOX1, EPCAM, NBL1, FOLH1, PMAIP1, PPP3CA, SLC2A3, ATP6V1B2, KIF5C, NINJ1, DSG2, ACTR2, IL1R1, CD9, HDLBP, C1orf116, COBLL1, CHMP1B, NFKBIZ, CANT1, ITPR2, NCKAP1, ENDOD1, ATP2B1, CGNL1, RIPK2, DOCK4, LAMB2, PGM3, EFNA1, IGF1R, CLDN3, NPY, PBX1, PLA2G2A, MFSD1, EGFR, SORD, CAP1, NFIX, PPAPDC1B, ECE1, TCEA3, ZNF704, KRT8, H2AFJ, NEDD4L, PNRC1, HEBP2, ERBB3, SLC12A2, TJP1, PEBP1, FAT1, C1orf21, RAB27B, CTTN, CNN3, NIPAL3, HIF1A, GNG12, HBEGF, FAM213A, BMPR1B, STEAP1, TSTD1, INSIG1, HSPA1B, ARG2, NPNT, TMEM141, ZNF827, AHNAK, BACE2, PART1, TMEM59, ADI1, SNRPN, BCAS1, CD302, APP, SKIL, KLF4, TM9SF3, SOCS2, ZFAND5, ENAH, TSPAN8, LIPA, CD151, RBFOX2, SLC2A12, MYO5C, MTHFD2, VWA1, TMEM98, GATA2, ETV3, HN1L, YAP1, GRHL2, ITGA6, ENTPD5, MYH10, NGFRAP1, EPHX2, TMEM87A, SYNE2, CEBPB, P4HB, CTSH, CYB5A, INADL, RAMP1, HIST1H2AC, ANTXR2, GOLGB1, KIAA1217, ZG16B, TMEM79, PM20D2, C1orf115, ANXA3, EIF4A3, PTPRN2, MERTK, PPDPF, TRA2B, ARHGAP6, DDAH1, AGR2, ZKSCAN1, MBOAT2, PHGDH, SH3BGRL2, FAM210B, CDC42BPA, AMOTL1, RASEF, TRIM2, TNFSF10, GLO1, MDK, ARID5B, MAP7, LSAMP, LMAN1, CLSTN1, TRGC1, CYR61, HSPD1, GFPT1, PEA15, BAG3, TMC5, SMIM14, IDH1, GREB1, B3GNT5, GDF15, NME4, ELF3, KIF21A, SLC4A4, SFN, RAB27A, USP53, ARHGAP29, GOLGA4, SLC39A7, CAB39L, STK39, ERLEC1, ZNF761, C12orf57, ALDH3A2, KRT19, SEMA3C, SCARB2, GCNT2, TRPM4, H1F0, CORO2A, MPC2, GALNT7, TRPM8, PAK1IP1, CASC4, NCAPD3, TFRC, FASN, RCAN3, PDXDC1, ACACA, TUBA1B, ANPEP, SPON2, REXO2, EPAS1, FAM46A, ANKH, NFKB1, ATP1B3, SLC40A1, ZFAND2A, ABCC4, ODC1, MIA3, ACSL3, LPAR6, SCD, ALOX15B, ERRFI1, DNAJA4 |
| 3 | T_cells | IL7R, TRBC2, CCL5, IFNG, CD8A, CD69, ETS1, CD3D, CXCR4, SPOCK2, CD2, PTPRC, TNFAIP3, TRAC, CD96, ICOS, LCK, PTPRCAP, CD3G, CD247, RP11-94L15.2, ITK, IL2RB, BCL11B, CD3E, RASGRP1, CD6, ITGAL, CD52, BTG1, CYTIP, IKZF3, FYN, GZMA, GZMH, IKZF1, TBC1D10C, CLEC2D, FYB, STAT4, HLA-B, PTPN22, ACAP1, IL32, KIAA1551, RUNX3, APLP2, SAMSN1, ARHGDIB, SRGN, GBP5, PCED1B-AS1, TAGAP, STK17B, PTGER4, CORO1A, RGS1, PPP1R16B, RAC2, WIPF1, ZFP36L2, NR4A2, PSAP, TMC8, RORA, AKNA, FAM46C, TRAF3IP3, CD48, CST3, SLA, IL2RG, ARHGAP9, DUSP4, ARAP2, ITGA1, RHOH, PPAP2A, GPRIN3, IRF4, STK10, SPN, ITGA4, HLA-A, CD53, KLK2, SLC2A3, TACSTD2, DSP, PARP8, TMSB4X, KLF6, KLK3, CCL4, CPE, RGCC, STK17A, ARHGAP30, SCARB2, GNG2, CD9, NKX3-1, FLNB, ACPP, RASAL3, STEAP2, CKB, SEPP1, NUPR1, DOCK10, CCR5, ALDH1A3, DOK2, YPEL5, BIRC3, RDH11, CREM, TSPAN1, TSC22D1, DHCR24, PTPRF, TMPRSS2, S100A4, PIP4K2A, KLF4, CTNND1, AZGP1, GOLM1, H1F0, ARL4C, RBM47, LCP1, CEBPD, GRN, NFIB, SPINT2, SLC44A4, CALD1, HDLBP, DUSP2, MBNL1, CYFIP2, TBX3, HOXB13, PDLIM5, NEDD4L, LCP2, HSP90AA1, APP, FSTL1, PMEPA1, NDRG1, APOBEC3G, CLDN4, SLC38A1, FXYD3, KIAA1244, STK4, SLC45A3, CELF2, ABCC4, EVL, BASP1, DSTN, CDH1, ASAH1, MLPH, IL1R1, VEGFA, CTSB, SYTL3, LAMB2, SERPINB6, TSC22D3, ADIRF, SPOCK1, ZFHX3, BIN2, DST, ABHD2, APBB1IP, CNOT6L, SLC39A6, TPM1, KRT18, EVI2A, CCND2, ELF1, ALCAM, P4HB, ALDH2, FNBP1, LIFR, RAB3B, STEAP4, MME, HLA-F, SYNGR2, AR, ATP8B1, CYFIP1, EHF, CTNNA1, CREB3L4, DNAJB1, HECA, CD59, FOXA1, DSG2, SMIM14, NPC2, PTRF, RRBP1, PDE4D, ATP2C1, LAMP2, KLK4, CD151, ACSL1, MSMB, IDH1, SLC30A4, ANKRD44, HSPA8, ZNF331, NEFH, LGMN, ADI1, EPCAM, GPR65, NPDC1, ACSL3, PTTG1IP, DHRS7, GUCY1A3, TM9SF3, FAM213A, FOLH1, AHCY, CALM1, DIAPH1, ISG20, SPDEF, IGF1R, CD63, MAOA, ALDH3A2, HOMER2, MLLT4, LIMCH1, GLIPR1, PFKFB3, ITM2A, NANS, CNDP2, PARM1, SYNE2, SORD, MSN, NCOA4, TNFRSF1B, ICAM3, H2AFJ, TMEM66, MTUS1, CDC42BPB, HMHA1, PCYOX1, CD97, IFITM3, DCXR, ERGIC1, RNF125, NGFRAP1, TMEM141, HSD17B4, ITGAE, KIAA1324, CYB561, C1orf21, FAM210B, PLXDC2, ATP6V0B, NPY, ENDOD1, CGNL1, CLDN3, GMFG, PAG1, HSBP1, MARCKS, WLS, PLXNB2, MAGED2, TMEM2, ARSD, PLA2G2A, EGFR, ACTN1, KRT8, C1orf116, CCND1, SCD, MYO6, JUND, NFIX, ENTPD5, DUSP5, RNF19A, BMPR1B, CTSH, GAA, RHOBTB3, GPX1, FNBP1L, EFNA1, CLEC2B, ALDH7A1, KCTD3, RASSF5, MAGED1, PEBP1, CANT1, PBX1, ALOX15B, COBLL1, ERBB3, FCGRT, SLC39A7, CNN3, PREX1, PGM3, PRDX6, AMOTL1, SRSF7, EMP2, TCEA3, NET1, TJP1, YBX3, MYOF, EZR, TCEAL4, ARG2, PPAPDC1B, MBOAT2, GATA2, JAK1, RNF213, NME4, CALU, ITGB5, LTBR, NCKAP1, SAMD9, MLEC, SMARCA1, ATF3, BACE2, MAFB, RAMP1, FAT1, STEAP1, CTBP2, TNFSF10, ST14, ADAM9, ZNF704, PPP3CA, ARHGAP6, HSPH1, DBI, TRPM4, NPNT, TMEM14C, DSC2, TUBA4A, ARHGAP25, SEMA3C, IL13RA1, GSN, HES1, CDC42BPA, SLC2A12, SH3KBP1, SEC11C, TMED3, HIST1H2AC, CSGALNACT1, MPC2, SAT1, PART1, SMCHD1, SH3BP4, MYH10, CYB5A, ATP6V1G1, CYR61, ANKH, SH3BGRL3, VWA1, GLO1, PDK4, GCNT2, AGR2, BLVRB, ACACA, SWAP70, TSPAN8, TSPYL2, C1orf115, SOX4, TPD52, ANPEP, GDF15, SLC12A2, GRHL2, ELF3, TIMP1, BCAS1, SASH1, MAP7, ZG16B, COLEC12, GALNT7, RBFOX2, IL10RA, RHOU, ENAH, LSAMP, REL, CTSL, SLFN5, YAP1, NFIA, MDFIC, BCAM, NBL1, PIK3R1, CAB39L, PRKCH, OSBPL1A, PAK1IP1, RASEF, MDK, SLC40A1, INSR, PALLD, GREB1, GLUL, A2M, LEPROTL1, TMEM79, DNAJA1, KIAA1217, KIF5C, ARHGAP29, FASN, RCSD1, DOCK8, CBLB, SFN, MCL1, LGALS3BP, TCF4, SOD2, DPP4, CD44, PERP, KRT19, TNF, NCAPD3, AKAP13, ZC3HAV1, LBH, DDX3Y, PIK3IP1, SLC4A4, IER3, ETS2, EMB, PPP2R5C, PNRC1, SRSF2, RASGEF1B, BCL2, 6-Sep, ADM, ATP2B4, SGK1, PRDM1, TIMP2, MACF1, HLA-DRA, DDX3X, NIPAL3, CLK1, BTN3A1, CCDC69, FOSL2, CCDC88C, SPTBN1, SMAP2, PDE7A, ID2, CYLD, NR4A3, CDV3, CDKN1B, MYADM, RARRES3, 9-Sep, IVNS1ABP, SEMA4D, RASA2, PDCD4, SUN2, PBXIP1, PLEKHA2, RABGAP1L, BTN3A2, ANP32E, ST3GAL1, PDE4B, ATF7IP, CDC42SE2, EML4, EGR1, FTL, RBL2, CHD1, DEDD2, KMT2E, MSL2, HERC1, LPIN2, PCNX, MGAT4A, DDX24, SYNRG, WNK1, ERAP2, KMT2A, FOXO1, PHLDA1, CNTRL, DDX6, HMGB2, RPS6KA3, BUB3, ANXA1, USP36, HLA-DRB1, OPTN, VPS37B, CHD2, CITED2, NR3C1, HERPUD2, FAM107B, ATM, HLA-DPA1, CHORDC1, SP100, RAB8B, JMY, CACYBP, SPTY2D1, HSPA1A, CD74, HSPD1, HLA-DPB1, NOP58 |
| 6 | Endothelial_cells | VWF, SELE, FLT1, IFI27, SDPR, PTPRB, DARC, EMP1, PLVAP, APOLD1, NES, SPARCL1, SLCO2A1, EMCN, EPAS1, A2M, ABCG2, PODXL, CD93, ENG, HEG1, KDR, TIMP3, SELP, IGFBP4, IGFBP7, THBD, ELTD1, PIK3R3, PLAT, CLEC14A, CXorf36, ADAMTS1, GPR116, TSPAN7, CD34, MMRN2, LEPR, GNG11, PTPRG, CRIP2, RNASE1, RAMP3, JAM2, HSPG2, NOSTRIN, TEK, LDB2, F8, ABCB1, DLC1, SPARC, FAM107A, ELK3, IL33, COL15A1, CAV1, TM4SF18, CDH5, NUAK1, LMO2, NNMT, KIAA1462, PCDH17, ADCY4, CFI, TIE1, EGFL7, SHROOM4, C1QTNF5, LRRC32, THSD7A, FGD5, COL4A1, LMCD1, ACVRL1, SHANK3, PLEKHG1, RHOJ, ZNF521, RUNDC3B, ARAP3, RAPGEF4, TGFBR3, ENPP2, GPM6A, HYAL2, GIMAP8, GSN, NOTCH4, EHD4, SULF2, RAPGEF3, GIMAP7, SYNPO, SH3BP5, GIMAP5, ITM2A, GIMAP4, ZEB1, TMTC1, ADAMTSL1, SPTBN1, ITGA9, TM4SF1, HLA-E, LIFR, ARHGAP31, FAT4, OLFM1, MET, TGFBR2, SPRY1, RAPGEF5, ENTPD1, TNFRSF10D, PLXND1, TINAGL1, PRSS23, MCAM, NRP1, VIM, IFITM3, FAM198B, PDLIM1, MECOM, LHFP, STOM, MGP, TCF4, TIAM1, CLU, SOCS3, ETS2, ITGA6, TUBB6, VCAM1, KLF2, PTPRM, NEURL1B, ITGA5, CLIC2, HLF, PPAP2B, CD109, GPRC5B, RAI14, WWTR1, APP, IFI44L, GJA1, ITGA8, EPHA4, MATN2, ERG, AFAP1L2, PTRF, TENC1, TXNIP, PALMD, VAMP5, HIP1, CCDC3, ITIH5, OSMR, CAV2, TACC1, PLS3, MEF2C, IL6ST, ITGB4, CEP68, LAMB1, IFITM2, CFH, PMP22, CCDC69, C11orf96, IFIT3, EFNB2, MLKL, IFITM1, GFOD1, FZD4, C10orf10, UACA, FAM171A1, CTNNAL1, ADAMTS9, CNN3, CX3CL1, BMPR2, MYH9, FOXC1, RND1, TMSB10, TGM2, DOCK9, CD59, FGD6, IFIT1, PLK2, CTGF, ETS1, DPYSL2, SMAD1, LCP1, FLI1, LAMA5, SWAP70, TPST2, ARHGAP29, PKP4, MKL2, SYNE2, DUSP6, SLC2A3, TJP1, IFI16, NCOA7, JAG2, GAS6, DLL1, MSN, AP1S2, ASAP1, GBP4, ADAM15, MCF2L, XAF1, SERPING1, SNRK, LRCH1, CLIC4, APOL3, CDKN1A, EZR, TSC22D1, SASH1, TNFAIP1, KCTD12, TLR4, EVA1C, KLF7, CRIM1, MX1, LAPTM4B, RASAL2, HTRA1, ID3, AFAP1, PPFIBP1, SSFA2, NKX3-1, SEC14L1, INSR, GRB10, KLK2, MEIS2, KLK3, PTTG1IP, RBM47, SPINT2, PRCP, ACPP, STEAP2, TMPRSS2, RGS16, PXN, ARL15, MYC, MGLL, ITPRIP, TACSTD2, LAMB2, WARS, DUSP23, IQGAP2, ICAM1, CKB, TMEM66, HOXB13, PLCB4, SHC1, TANC1, DOCK4, VAT1, TSPAN1, HPCAL1, SLC25A25, SLC39A6, VAMP8, DHCR24, SLC9A3R2, PRKCH, TPD52L1, ACSL1, CDH1, NPDC1, RAB3B, PIK3C2A, AZGP1, KIAA0355, KBTBD2, FXYD3, EHF, FOXA1, CD44, C1orf115, MLPH, SLC45A3, KRT18, PMEPA1, SORL1, CLDN4, VEGFA, PITPNC1, ARHGEF3, SPDEF, KLK4, RDX, QSOX1, BASP1, PTPN12, SLFN5, CHSY1, ID1, KIAA1324, ALCAM, KLF4, DCXR, AMD1, IRS2, TBX3, NEFH, HOMER2, HIPK3, OSBPL1A, MME, GUK1, TPD52, BHLHE40, EPCAM, SPTAN1, MSMB, ALDH1A3, MYO1C, ITM2C, XBP1, RDH11, STEAP4, ARG2, SCD, ANTXR2, SORD, YBX3, SLC44A4, ECE1, CREB3L4, ITGB1, AIM1, DSG2, FTH1, TSTD1, FOLH1, DHRS7, KIF5C, MPC2, PLCB1, CEACAM1, PNP, LITAF, H2AFJ, CLDN3, AKAP12, MAP4, EMB, RCAN3, BMPR1B, C1orf116, NPY, NEDD4L, PARM1, P4HB, NEDD9, HDLBP, ERBB3, STK17B, ALOX15B, PLA2G2A, ST14, VWA1, DSC2, PTPRF, FLOT1, IL4R, PART1, PINK1, ADI1, SEC11C, FAM129A, GFPT1, KRT8, FAT1, DBI, GRHL2, BCAS1, STEAP1, LIMA1, ANKH, AGR2, NOTCH2, IDH1, TRGC1, FASN, SERTAD1, GALNT7, SLC2A12, PHGDH, SC5D, LEPROT, ELL2, ZFP36, ANPEP, SLC4A4, ZG16B, TC2N, ABHD2, SEMA3C, RAMP1, FBP1, ALDH3A2, MBP, GDF15, RHOBTB3, SERP1, MAP7, ERGIC1, TSPAN8, NCAPD3, LRIG1, ARHGAP6, ENTPD5, PPAPDC1B, RASEF, TAOK3, GPT2, ELF3, SAT1, ACSL3, RGS1, PTPRC, RNASET2, DPP4, ATP6V1G1, HIST2H2BE, RAB27B, PDE4B, CAB39L, SLC30A4, MAF, ELOVL5, ZNF761, RGS2, SYAP1, SPOCK1, NANS, PERP, HIST1H2AC, ZNF331, GADD45G, FTL, KIAA1244, PGM3, SLC38A1, NAAA, CTSD, ZNF827, HIST1H2BD, H1F0, CXCR4, ZCCHC6, ATP2C1, MCCC2, CPE, PIK3R1, CANT1, PPP3CA, HN1L, ITPKC, TRPM4, LAPTM5, ASAH1, NUPR1, YPEL5, NIPAL3, LGMN, CYB561, GOLM1, PAK1IP1, SLC12A2, SMAP2, DSP, NEAT1, TNFAIP3, RASD1, ARL4C, GCNT2, KLF6, PDLIM5, SOD2 |
| 9 | Smooth_muscle_cells | DCN, FBLN1, COL1A2, IGF1, CCDC80, SFRP1, COL3A1, LTBP4, C1S, SERPINF1, COL6A2, MMP2, COL6A1, COL14A1, SPARC, PDGFRA, FBN1, ALDH1A1, SPARCL1, SLIT2, AEBP1, PRRX1, LAMA2, NR2F1, PDGFRB, GPR124, FBLN2, CTSK, LAMA4, DDR2, PCDH18, COL8A1, FBLN5, MEIS1, SRD5A2, RP11-166D19.1, TMEM119, CFD, EDNRA, COL5A2, MXRA5, IGFBP5, FN1, MGP, MATN2, APOD, ECM2, C1R, RARRES2, PRELP, PPAP2B, IGFBP7, BGN, F10, FLRT2, BMP4, DKK3, RORB, FXYD6, COL15A1, VSTM4, AKAP12, ISL1, COL16A1, MRC2, CRISPLD2, GPC6, FGFR1, KANK2, ESR1, SULF1, DZIP1, LRP1, OAF, SPON1, TIMP2, SERPING1, CXCL12, IGFBP4, MFGE8, VCAN, GSN, OLFML3, PCDH9, ZFHX4, OSR2, COL18A1, SSPN, LGALS1, PID1, CPED1, TIMP3, COL4A1, S1PR3, SYNE1, PTN, FRMD6, CYBRD1, BOC, LHFP, MAP1B, COL12A1, PHLDB1, NFASC, CALD1, FAT4, TNC, MRVI1, PMP22, ZCCHC24, CFH, SYNPO2, TAGLN, EPHA7, CTGF, PAM, RUNX1T1, VIM, BICC1, LAMB1, SETBP1, PRKCA, NES, CYP1B1, LTBP2, GAS6, SPTBN1, TCF4, ANTXR1, ZEB1, TNS1, ACTA2, FSTL1, SGCD, SDC2, EPB41L2, PEAK1, RNF150, MYLK, NR2F2, FZD7, BNC2, ARHGEF40, JAM3, PALLD, ITIH5, RDH10, LHFPL2, RAB34, AHNAK, 11-Sep, DPYSL3, IL6ST, APCDD1, CAMK2N1, SPRY2, TIMP1, ASPH, LTBP3, HTRA1, LAMB2, B2M, FLNA, ADD3, SDC3, MEIS2, TUBA1A, MXRA7, TACSTD2, KLK3, GAS1, NFIA, NDN, JAZF1, NEURL1B, SGCE, PKIG, KLK2, DST, SH3PXD2A, PTCH1, PTPRG, DSP, LMO4, EFEMP1, RND3, PTRF, LCP1, A2M, GLG1, S100A6, DPYSL2, ACPP, PBX1, SH3D19, PLEKHA5, SYNGR2, NKX3-1, FHL2, RBM47, TMEM47, RGL1, CCND2, SLC16A2, TSPAN1, PRNP, ADCY3, AZGP1, ADAMTS1, TMPRSS2, LGALS3BP, RBFOX2, TPD52, EVC, FOXC1, CLDN4, HOXB13, DHCR24, FERMT2, PLXDC2, LAMC1, PKD2, SLC44A4, IQGAP2, SPINT2, ARHGAP10, VAMP8, NBL1, ROBO1, KDELC2, RPS6, PABPC1, KRT18, VCL, SH3BP5, LTBP1, SEC11C, MCC, CLIC4, KIAA1244, ABLIM1, SLC45A3, AIM1, CNN3, FXYD3, IFITM3, LRRFIP1, LPHN2, FLNB, RPL8, MLPH, ST6GAL1, CD9, ZBTB20, RAB3B, EHF, HLA-B, PTPRF, APP, DPP4, MMP14, CDH1, FOXA1, RDH11, PTPN13, LRP6, FMOD, NANS, SAT1, KLK4, FAM178A, SPDEF, NEFH, CNDP2, KIAA1324, CREB3L4, CST3, CYR61, PLEKHB2, ATP8B1, NEDD4L, ARG2, HOMER2, KLF6, MSMB, RCAN3, PPFIBP1, HERPUD1, DSG2, BACE1, HLA-DRA, EPCAM, NIPAL3, HIST1H2AC, SCD, DCXR, SPIN1, SORD, C1orf116, CRTAP, CD74, RPS19, KRT8, FOLH1, CLDN3, BASP1, HIPK2, ODC1, CTSF, NPY, SLC30A4, PTGFRN, PARVA, PPAPDC1B, SERP1, AMD1, H2AFJ, CKB, ALDH1A3, HLA-DRB1, THBS1, PDLIM5, SLC38A1, GNG12, ATP6V1G1, NAMPT, NFIX, DHRS7, PLA2G2A, HN1L, ATP2C1, ACSL1, STEAP4, PART1, TMEM141, CD46, RAB27B, ENC1, TGM2, CGNL1, ALOX15B, CYTH1, CORO2A, CYB561, EZR, SRGN, GCNT2, ATP6V0B, TRPS1, TSTD1, REV3L, EMB, ITGA6, GRHL2, UBE2J1, TRGC1, ZG16B, COBLL1, BCAS1, RAB27A, GPX1, ABCC4, UACA, DBI, MAP7, AGR2, H6PD, MAOA, CSRP1, NCAPD3, HIST2H2BE, TAOK3, NFIC, SGK1, ELF3, GALNT2, PTPRC, FBP1, CD302, RGS1, TMEM43, NAAA, MPC2, GDF15, NDRG1, MBOAT2, LAPTM5, NCOA4, PERP, GALNT7, TMEM79, IDH1, STEAP2, SPG20, GOLM1, P4HB, RAB11FIP1, EBP, TRPM4, ENTPD5, SORL1, LDLR, PMEPA1, C1orf115, SLC2A12, CREB3L2, TSPAN8, ANKRD37, CDC42BPA, MYH10, APOL1, CPE, NEAT1, CXCR4, TNFAIP3, PAK1IP1, HLA-DPB1, TRIB1, NR4A2, TCF12, NUPR1, PDE4B, LAPTM4B, INSIG1, SMAP2, CDKN1A, ZNF217, NFKBIA, MTUS1, ARHGAP21, ZNF331, PPAP2A, HLA-DPA1, EGR1, SLC39A6, ARHGDIB, MME, LIFR |
| 11 | Tissue_stem_cells | MYH11, RGS5, ACTA2, TAGLN, MYL9, C11orf96, MYLK, MCAM, LMOD1, SYNPO2, FHL1, TPM2, RCAN2, COL14A1, SLIT3, AEBP1, CNN1, NOTCH3, MRVI1, ITGA8, FRZB, NTRK2, AOC3, BGN, ITGA7, NEXN, FHL5, PDE3A, LGI4, SUSD5, ACTG2, CSPG4, C2orf40, KCNAB1, CASQ2, GJA4, PRELP, PRKG1, ADCY5, MSRB3, KANK2, CCDC3, CDH6, MAP1B, NFASC, IGFBP7, TINAGL1, SYNM, PDGFRB, PRRX1, FILIP1, DAAM2, MFGE8, TNS1, DMD, TIMP3, SPARCL1, FLNC, PLCE1, CAV1, SPEG, LTBP1, FLNA, FILIP1L, ECM2, TPPP3, EFHD1, EDNRA, NPY1R, COL18A1, SPARC, CAP2, MYOM1, RRAD, PPP1R12B, ITIH5, CALD1, COL4A1, COL1A2, COL3A1, NEURL1B, ADAMTS1, APOLD1, COL6A1, EHD2, S1PR3, WTIP, SORBS1, ANGPT2, PALLD, GPR124, PDE1A, CPED1, ADCY3, PGM5, SMTN, EPS8, SORBS2, MGP, RERG, CAV2, LHFP, TPM1, JAG1, PTRF, PLS3, A2M, RBPMS, VSTM4, COL6A2, CRIM1, VCL, VIM, ATP2B4, RASL11A, MLTK, GRK5, NR2F2, ANO1, PDLIM3, ITGA1, SYNPO, TMEM47, HSPB8, COL12A1, EPAS1, DSTN, DDR2, FN1, ANTXR1, IGFBP5, CRIP2, RNF152, FOXC1, MAP7D3, LAMA4, MYL6, TLN1, ARHGAP10, DKK3, LPP, SLC7A2, IGFBP4, HIP1, FRY, SOD3, MEF2C, CACNA1H, TIMP2, SYNE1, DST, ADIRF, ACTN4, TMTC1, LGALS1, GEM, LBH, MPRIP, PHLDB2, CSRP1, ZEB2, PPAP2B, ARHGEF17, PKIG, PLEKHG3, ANGPT1, CRYAB, UTRN, MGLL, DGKH, CLIC4, PPP1R12A, RRAS, SERPING1, S100A4, FZD7, RHOB, C10orf10, ARHGEF10L, FERMT2, LCP1, TNC, ITGB1, MAPRE2, FAM129A, MICAL2, SULF1, KCNMA1, SYT11, SAT1, ACTN1, S100A6, SDC2, AKAP12, TUBB6, SELM, C12orf75, C9orf3, SYNE2, MTHFD2, RAB31, CLMN, LAMC1, ENAH, TACSTD2, MYO1B, MAP2, KLK3, ENTPD1, KLK2, SORT1, CORO1C, NKX3-1, GADD45B, ID4, PARM1, VASN, LAMB2, DSP, TBC1D1, 7-Sep, SOCS3, TPT1, DPYSL3, PPP1R12C, KLF2, CDKN1A, ACPP, IQGAP2, CRTAP, ANXA6, RBM47, ALDH1A3, TSPAN1, CNDP2, RGS16, TPD52, TMPRSS2, FLNB, RND3, GPX3, HOXB13, EGR1, SVIL, VAMP8, AZGP1, FBLIM1, SPINT2, BASP1, TACC1, SH3BGRL, LMO4, SLC44A4, RDH11, B2M, DHCR24, C1S, NEDD4L, GOLM1, AIM1, STEAP2, ANO6, KIAA1244, FOS, NDRG1, PTK2, IFITM3, ABCC4, NDN, UBA2, TUBA1A, FXYD3, CSRP2, TMEM66, CCDC107, PRKAR2B, CAMK2G, HERPUD1, ILK, SERP1, BTG2, DPP4, THBS1, SLC45A3, RAB3B, CYB561, CD151, PPP3CA, YBX3, LMNA, C1R, FOXA1, MLPH, CREB3L4, PDGFA, NOV, DCXR, CTSH, MACF1, CLDN4, KLK4, LAMA5, TMBIM6, SPDEF, EHF, CDH1, MYO1C, NEFH, MSMB, KIAA1324, ROCK2, MME, SCD, NANS, DHRS7, RBFOX2, TNFSF10, XBP1, ZFP36, DSG2, EPCAM, ATF3, SEC11C, ACSL1, CD74, ARPC1A, PHF17, GUCY1A3, SPOCK1, HLA-A, SORD, ANKH, ATP2C1, ARID5B, MYC, KLF9, IDH1, CPM, JUNB, MPDZ, SLC30A4, SEPW1, KRT18, PTPRF, CYB5R3, NPNT, P4HB, FOLH1, PDCD4, AMD1, SC5D, C1orf116, MLLT4, IL1R1, PLA2G2A, NIPAL3, KRT8, ARG2, CLDN3, HLA-DRB1, NPY, ROCK1, S100A10, HOMER2, HLA-B, PPAPDC1B, RPN2, TSTD1, SYNGR2, SCRN1, PGM3, CYR61, ITGA6, STEAP1, STK17B, TMEM141, ST6GAL1, CGNL1, LAMP2, ERP29, MBOAT2, HLA-DPA1, WDR1, SOAT1, EMB, 8-Sep, ST14, BMPR1B, HIST1H2AC, ADAM10, TNFAIP3, RCAN3, PAWR, CANT1, ENTPD5, DSC2, BCAS1, ALOX15B, INPP4B, GALNT7, GCNT2, GRHL2, PART1, CEBPD, SLC39A7, PPAP2A, ZNF217, MPC2, KIAA0319L, ERBB3, RAB27B, ELF3, NPC2, PDE4B, ZNF761, NFKBIA, AGR2, FAM111A, ZCCHC6, MARCKSL1, HIST1H2BK, PTPRC, ZG16B, SSR4, GDF15, C1orf21, PLXDC2, NCAPD3, MAP7, PAK1IP1, HLA-DRA, ABR, SLC39A6, RRBP1, PDIA6, EZR, RNASET2, PDIA4, NET1, MBP, SLC2A12, TRGC1, EBP, DBI, HLA-DPB1, FRMD4A, TPM3, ITPR2, PLCB4, CCND1, SLC12A2, NRP1, MCCC2, VEGFA, ZBTB16, SMOC2, SLC39A10, RAB11A, FASN, PERP, TRPM4, ATP6V1G1, CXCR4, PSAP, SGK1, RGS1, NCOA4, FBXO32, MT2A, GRN, ID2, ZNF827, GPX1, TMSB10, INSIG1, H2AFJ, IVNS1ABP, SLC25A4, RGS2, TSPAN8, TRIB1, KLF6, SRGN, NR4A1, ARHGDIB, HSPH1, PDK4, ANTXR2, ANXA1, ODC1, KIAA0040, LIFR, FTL, SEPP1, PLIN2, NFIL3 |
| 12 | Macrophage | APOE, GPNMB, APOC1, LYZ, MMP9, CD68, FPR3, CD163, SLAMF8, NCF2, CYBB, FCER1G, LILRB4, C5AR1, MSR1, SLC7A7, IGSF6, TYROBP, SLC15A3, IFI30, TREM2, TFEC, C1QB, ITGAX, CD84, TMEM176A, TMEM176B, OLR1, CYP27A1, CECR1, CTSS, CD14, CAPG, BCAT1, SLC37A2, EREG, LHFPL2, C1QC, ITGB2, MS4A7, PLEK, CD86, CCRL2, PLAUR, EMILIN2, LIPA, C1QA, GM2A, ABCA1, NR1H3, CTSC, LAIR1, LAPTM5, CMKLR1, LGALS1, PILRA, TGFBI, VSIG4, GPR183, AIF1, SERPINA1, CTSD, FCGR2B, PTAFR, LGALS9, NRP2, FERMT3, C3AR1, HLA-DRB5, HMOX1, LY86, TYMP, HLA-DMB, RASSF4, CTSZ, CTSL, DMXL2, HLA-DQA1, CD4, G0S2, CTSB, SIRPA, FCGR2A, CPVL, PSAP, FTL, PLXNC1, KLHL6, PLA2G7, CCL3, SLCO2B1, HLA-DMA, PLBD1, MT1G, CSF1R, IL8, FGR, CXCL2, GRN, IL18, TNFAIP2, CD83, SLAMF7, PIK3AP1, MPEG1, CTSA, TFRC, BCL2A1, NCKAP1L, SLC16A3, MPP1, IL1B, CD53, MITF, CFD, ASAH1, HLA-DRA, FTH1, IRF8, THEMIS2, HLA-DRB1, SYK, CREG1, HLA-DQB1, SLC43A3, MS4A6A, NINJ1, CYBA, MMP14, RAB20, SLC31A2, SNX10, PLIN2, RAB31, PMP22, ZEB2, DAB2, IL1RN, RNF130, CD74, HLA-DPB1, CORO1C, ATP6V1B2, SAMHD1, HLA-DPA1, KCTD12, NPC2, LGALS3, IER3, EPB41L3, PLXND1, TNFRSF1B, TIMP2, GPX1, SLC8A1, CHST11, MT1M, VIM, AKR1B1, LGMN, CD36, BMP2K, GLA, MT1F, ICAM1, TNS3, CD48, RGS1, TSPAN4, PLXDC2, A2M, MAFB, CSTB, FGL2, HLA-DQA2, SDCBP, FMNL2, GSTO1, CTSH, NAGK, FCGR3A, ARPC1B, GLUL, LYN, TMSB10, AOAH, CD63, LRP1, MFSD1, TPP1, SOD2, PLD3, SRGN, CXCL16, ANXA2, LACTB, ATP6V1F, GNS, SGK1, CCDC88A, DUSP2, PGD, RASGEF1B, FAM49B, SAT1, M6PR, WARS, LIMS1, NCEH1, UPP1, CD81, VAT1, MT2A, FKBP15, GNA13, NFKBIA, PPT1, RNASE1, LTA4H, H2AFY, ARL4C, MGAT1, HEXB, RAPGEF1, PPIF, FCGRT, HEXA, TMSB4X, MYO9B, MGLL, CEBPB, MT1X, TNFAIP3, C15orf48, GRB2, PRDX1, TCIRG1, ARPC5, BRI3, PDE4DIP, RNASET2, ZNF331, ATP6V0B, ATP6AP2, CD44, SCPEP1, CPM, ENO1, HIF1A, FUCA1, SH3BGRL3, LITAF, ANXA5, NFKB1, LAMP1, CHMP1B, TXNRD1, TKT, ZFAND5, DSP, DNASE2, PPAP2A, ADM, STAT1, CYB561A3, MARCKS, HBEGF, TACSTD2, FCHO2, CYFIP1, KLK3, NKX3-1, ALDH1A3, FBP1, CPE, CALD1, STEAP2, TMPRSS2, CKB, ACPP, PKM, KLK2, PMEPA1, NFIB, AZGP1, GBP1, TSPAN1, KIAA1244, PTPRF, FSTL1, HOXB13, TBX3, AR, RAB3B, LIFR, SLC44A4, ADIRF, EIF4A3, FXYD3, EHF, SPDEF, KRT18, LRIG1, SPOCK1, SLC45A3, FOXA1, CLDN4, KIAA1324, KLK4, CREB3L4, LIMCH1, SYNE2, MT1E, STEAP4, DSG2, CDH1, TPD52, NDRG1, DSTN, SLC30A4, DHCR24, PERP, PARM1, MME, PPP3CA, KIF5C, NEFH, HOMER2, NPDC1, MSMB, DPP4, PTRF, FOLH1, ABCC4, FLNB, CNN3, TPM1, MYO6, ATP8B1, CGNL1, C1orf116, EPCAM, ERBB3, PBX1, SPTBN1, EGFR, HEBP2, NBL1, NCKAP1, CSGALNACT1, SLC39A6, GOLM1, ECE1, SORD, MLPH, MTUS1, GUCY1A3, NIPAL3, BCAS1, TCEA3, HIST1H2AC, NFIX, BMPR1B, TJP1, CTTN, EFNA1, SOCS2, PM20D2, INADL, KRT8, FAT1, ARG2, MYO5C, ITM2C, COBLL1, GOLGB1, TNFSF10, ZNF827, GATA2, NPNT, NEDD4L, NPY, CYB561, STEAP1, MAOA, GRHL2, SLC2A12, SLC12A2, RAB27B, FNBP1L, SLC38A1, TSPAN8, CLDN3, MAP7, PPAPDC1B, AGR2, MAGED1, PLA2G2A, MBOAT2, MLLT4, MYH10, PART1, BACE2, NET1, ZNF704, PTPRN2, ARHGAP6, TRGC1, RDH11, NGFRAP1, ENTPD5, AMOTL1, RAMP1, IGF1R, DHRS7, IFT57, CCND1, C1orf115, GCNT2, NCAPD3, SNRPN, ENAH, HIST2H2BE, ENDOD1, HN1L, ELF3, NFIA, FASN, DCXR, ARHGEF12, CANT1, HIST1H1C, PDLIM5, PGM3, UTRN, TRPM4, MAGED2, GFPT1, AMD1, GLO1, SC5D, SEC11C, ODC1, MIA3, KLF9, GOLGA4, DYNLL2, H2AFJ, RCAN3, H1F0, ZKSCAN1, PAK1IP1, ANTXR2, LAMB2, APP, IL1R1, ADI1, ANKH, SPINT2, RAB27A, TM9SF3, MPC2, ATP2C1, IL6ST |
| 13 | NK_cell | IL1RL1, VWA5A, MAOB, HPGD, CD22, IL18R1, TNIK, ACSL4, CD69, RAB37, GPR65, GRAP2, DHRS9, PTGS1, LAX1, BTK, BMP2K, ARHGEF6, GATA2, DLC1, TMEM154, RAC2, SDPR, ALDH1A1, SAMSN1, CD84, ZEB2, CADPS, MITF, SLC2A3, SRGN, DUSP6, PTGS2, LMO4, RASSF5, ALOX5, MYB, AHR, NR4A3, RHOH, FOSB, VIM, ARHGAP25, CD44, STX3, CD83, CSF1, PRKCB, NFKBIZ, LMNA, LAT2, ALOX5AP, ANXA1, FER, EMR2, EGR3, NR4A2, FCER1G, CLU, ARHGAP18, MYADM, GLUL, RASGEF1B, NFKBIA, GALNT6, RGS1, PAG1, PPP1R15B, ARHGDIB, LAPTM5, FTH1, NR4A1, GNPTAB, PPP1R15A, CD82, EMILIN2, FXYD5, LCP2, CNRIP1, BIRC3, UBB, AGPAT9, EHD1, CTTNBP2, DDX3X, SDCBP, MIR24-2, RGS2, CCDC88A, ALS2, ELF1, NFKBID, DDX3Y, CTNNBL1, IKZF1, RAPGEF2, C10orf128, KDM6B, TDRD3, DDX26B, DOCK11, CAPG, IL4R, BCL2A1, FOSL2, EIF1, BTN2A2, ELL2, PRNP, SPECC1, CHN2, ITGA9, KLHL6, PTAFR, LEO1, AKAP13, RASSF2, PLIN2, GALC, LYST, AGAP1, ARL5B, SYTL2, SGK1, AHNAK, FOXP1, DNAJA1, ZNF331, SELK, DDX5, GAS7, ITGAM, SOX13, LAIR1, LPCAT2, IER5, TSC22D2, LAPTM4A, ACTG1, VAT1, HEG1, ASAH1, TIAM2, STMN1, BHLHE40, SLC44A1, KLK3, CELF2, SERPINB1, B4GALT5, PARP4, CREM, S100A6, TACSTD2, NDRG1, YWHAZ, PLEKHA2, DSP, HSP90AA1, AGPS, TUBA1A, MSRA, RBM39, NKX3-1, STK17B, KLK2, OSBPL8, ITPR1, CEBPD, SH3KBP1, CKB, CPE, RDH11, ALDH1A3, ANKRD27, CSRNP1, RBM47, NFE2L2, HEXIM1, NUPR1, TNFAIP3, TMPRSS2, SCARB2, ICAM1, MCL1, DHCR24, SIK1, KLF6, HSPH1, STEAP2, HSPA1A, IL1R1, EPB41L1, PRKX, SQSTM1, NFIB, GLG1, TP53BP2, STK4, TBX3, SEPP1, AZGP1, FERMT2, CPNE3, SLC44A4, EXTL3, SH3BGRL3, HOXB13, UTRN, CLDN4, TPST2, FXYD3, CALD1, MLLT4, LAMP2, APLP2, APP, SF3B1, FSTL1, HIF1A, TSPAN1, GM2A, PTPRF, CPEB4, FAM53C, EIF5, GOLM1, TAGLN2, CNDP2, TSEN54, ANXA2, C21orf91, REL, PPAP2A, KIAA1244, SRSF3, JUND, CDH1, ACPP, NEFH, KRT18, ANKRD28, RHOB, CTSH, FAM46A, STXBP5, NEDD4L, SLC39A6, LAMB2, ADIRF, AIM1, TIAM1, FOXA1, BLVRA, PCF11, KLK4, EHF, TPD52, STEAP4, ABCC1, SPOCK1, HERPUD1, CTNND1, LIFR, NANS, CREB3L4, CYB561, RAB3B, SRSF5, PERP, MSMB, NPDC1, SH2B3, ANKH, TSPYL2, DHRS3, EGR1, PDLIM5, TIPARP, AR, ACSL1, EPCAM, ATP8B1, SPDEF, DSG2, IGF1R, MAOA, GUCY1A3, CNST, KIAA1324, PMEPA1, TMBIM6, RHOBTB3, SETD7, ATP2C1, DCXR, MTUS1, PIK3CA, FOLH1, DUSP10, RBBP8, MEIS2, CSGALNACT1, SLC38A1, H2AFJ, PARM1, ATXN1, HDLBP, SYNE2, MME, C1orf116, PLA2G2A, NDEL1, ALDH2, CGNL1, KCTD3, FKBP5, DPP4, FAM210B, MAPK6, RBMX, HOMER2, SLC30A4, TPM1, ALDH9A1, RHOC, DHRS7, SLC12A2, LIMCH1, KIF5C, ACACA, HIST1H2AC, MBOAT2, MYO1C, REEP5, TJP1, CANT1, PTMA, RHOU, CYB5A, EFNA1, SORD, ARHGAP5, RAB11A, ALOX15B, ACTN1, AHCY, PNPLA8, ITGA6, CLDN3, ERGIC1, KRT8, ERBB3, IGF2R, HLA-DRB1, RAB27B, MAP3K8, PPAPDC1B, ITGB5, BMPR1B, LPP, CD74, ALAS1, H1F0, NBL1, PYGB, FAM129A, WLS, STEAP1, LGMN, MPC2, MAFB, ZNF827, CORO2A, PART1, GADD45G, ATP1A1, NPNT, GCNT2, HLA-DPB1, DSC2, PLXNB2, MYOF, ENAH, C1orf115, CNN3, NGFRAP1, IL13RA1, AGR2, BCAS1, SMARCA1, TMEM98, MYH10, EMP2, ZNF532, PSMA4, PTOV1, SLC2A12, HLA-DRA, NIPAL3, TRGC1, SC5D, MAGED1, SKIL, ADAM10, CD151, HINT1, SYAP1, NFIL3, MARCKS, H2AFY, SEC11C, RAMP1, MTCH1, ADAM9, ALDH3A2, SH3BP4, SWAP70, EGFR, P4HB, TRPM4, RCAN3, UBA7, ECE1, ZFP91, HMGCS1, PM20D2, ATF3, ENDOD1, ENTPD5, RAB2A, RANBP2, SEMA3C, IFITM3, NFIX, PRDX3, GRHL2, LIMA1, PDE4B, ACSL3, CPD, MT2A, ALCAM, EMB, NPY, NCAPD3, TM9SF3, DSTN, ELF3, PPA1, INADL, MYO6, CST3, CYLD, ST6GAL1, GALNT7, PPP1R10, FAM107B, SMARCA4, ABCC4, ARFGAP3, PGM3, IDH1, ZNF217 |
| 14 | Neutrophils | S100A9, TREM1, AQP9, FPR1, CSF3R, MNDA, IL1R2, G0S2, OSM, NCF2, SLC11A1, C5AR1, IFITM2, IL8, PLAUR, CLEC4E, BCL2A1, PTGS2, MXD1, FAM65B, SRGN, RGS2, NAMPT, IVNS1ABP, AMICA1, GCA, PLEK, CD55, MYO1F, CTSS, FTH1, MX2, SOD2, LCP1, SERPINA1, PTPRE, NCF4, PTPRC, TMEM154, TYROBP, LRRK2, LYN, FCGR3A, HLA-B, RPS6, GMFG, GNB2L1, IFRD1, RPS5, RPL18, ICAM3, RPS4X, SAT1, LAPTM5, EEF2, MT-ATP6, HSP90B1, RPL8, SMAP2, RPS14, JUN, ALOX5AP, RPL3, SMCHD1, RASSF2, EVI2B, RPLP0, ARRB2, IL1B, ADAM8, RPL11, PREX1, RPS8, RPL13, AHNAK, RPL19, ITGAX, ATG16L2, FYB, NABP1, DUSP1, B2M, SLA, H3F3B, GLUL, MT-ND2, RPL5, MT-ND4, RPS19, FCGR2A, RPS3, RPL23, RPS18, RNF149, HLA-E, GBP5, RPL15, C10orf54, RPS12, RPL4, RPL13A, HLA-C, PHACTR1, MT-CYB, RPS11, LST1, CANX, RPS23, NCL, LCP2, CD81, ATP5B, USP15, RPLP1, P4HB, RPS20, SSH2, RPL30, LITAF, MCL1, CD53, SLC16A3, EIF3A, PILRA, COX4I1, RPS21, RPL35A, RPL37A, TLR4, BASP1, RPL27, MALAT1, NUCKS1, ATP1A1, MT-CO1, HCLS1, XPO6, RPL31, RPL10A, RPLP2, KTN1, MT-CO3, MBOAT7, GLTSCR2, PDIA3, NACA, RPL27A, CIRBP, RPS16, EEF1A1, SDCBP, CLTC, RPS13, SLC25A6, FOS, AMPD2, NDRG1, EID1, ARHGAP9, GNAS, IL6ST, TM9SF3, RPL34, MT-ND5, RDH11, CALR, MTDH, TRIB1, HCK, NAMPTL, SOD1, HDLBP, RPN2, MT-CO2, RPL35, TTC3, PPAP2A, PEBP1, GPR65, MATR3, KLK2, MACF1, ILF3, HSPD1, DSTN, KLK3, FLNB, THEMIS2, ENO1, TSC22D1, EZR, TMED10, SCARB2, CTSB, ANXA5, CTNNA1, XBP1, TRAM1, CAPNS1, ATP5A1, MAGED2, ATF3, C17orf76-AS1, ARHGDIB, PPIB, CAST, PRDX6, MT-ND1, RPS2, EIF4G1, NR4A1, RPS4Y1, LDHA, COPB2, RPS24, PMEPA1, SAMSN1, BIN2, GOLGB1, PFDN5, RPL12, MEGF9, ATP2C1, BAZ1B, CCT3, CPE, TACSTD2, CD9, CNDP2, HIPK2, RRBP1, PKM, PRDX1, SLC25A3, UTRN, HLA-A, IFIT2, MRFAP1, EIF3H, ALDH1A3, HNRNPM, NFE2L1, SERPINB6, RPL7A, STEAP2, APBB1IP, 2-Sep, EIF4B, RPL24, PDIA6, ACADVL, LMAN1, NDUFA4, SPINT2, ANXA2, GOLM1, SEC31A, LINC00657, RPL14, SEC11C, AZGP1, DSP, SYNCRIP, HSP90AB1, YWHAE, DYNLL1, RPS25, SPTBN1, EHD1, DYNC1H1, DHCR24, PSMA7, C6orf48, NAP1L1, NKX3-1, DBI, NOLC1, SLC39A6, CALM1, NUMA1, ABCC4, GOLGA4, CLDN4, ADI1, EIF2AK1, SEPP1, GLO1, ARHGEF12, ATP6AP1, TMPRSS2, PDLIM5, COX7C, CKB, REEP5, PSMD8, DHRS7, CALU, RPL38, TSPAN1, RPS15, GPX1, CST3, PHB2, SND1, IFIT3, MLEC, RPL32, CHD1, RHOC, ANXA1, CALD1, KIAA1551, SLC25A37, TAGAP, PCM1, RPL37, ACSL1, SSR2, BTF3, GSPT1, CD164, KLF9, TOP2B, SEC14L1, PDIA4, AIF1, SRSF7, SLC44A4, PTPRF, TNFRSF1B, SNRPD2, HSPH1, SURF4, MIA3, FAM129A, PABPC1, DCXR, HINT1, H1F0, LDHB, AIM1, GLG1, DDB1, SRSF2, PTMA, NUPR1, FSTL1, YWHAQ, SNRNP200, ZKSCAN1, EPRS, APLP2, ATP2A2, KRT18, SRP14, GADD45B, MAP4, HADHA, CUTA, EIF5B, ZBTB10, MSMB, CLSTN1, XRCC6, SEC62, TMEM87A, AKR1A1, HSD17B4, SSR3, SLC45A3, NFIB, NFE2L2, AMD1, VAMP8, CDKN1A, SEC61A1, HNRNPR, RPL22, TPM1, RSL1D1, TMEM123, AHCY, SSR4, KIAA1244, SYNGR2, DDX24, VASP, TUFM, DPP4, RHOB, ERLEC1, TPD52, CDH1, ITGB1, ANKH, APP, SRP72, RPL10, RPS29, THUMPD1, NEDD4L, PCYOX1, GAS5, SLC25A5, LRIG1, ADIRF, EIF3E, CD97, ZFHX3, CTSH, HOXB13, YPEL5, KDELR2, EHF, NARS, ARHGAP5, H2AFJ, ENDOD1, CTNND1, SEC63, CYFIP1, DCAF7, ACSL3, ECH1, GLUD1, VMP1, CYB561, TMEM14C, CCT2, HNRNPAB, TMSB10, SCP2, DDX42, ACPP, EIF3L, IVD, TMED9, COPB1, PSMB1, ALDH2, NIPAL3, TUBB4B, SPCS1, KLF4, TBX3, NRIP1, NANS, CTNNB1, SLC39A7, PHB, MAGED1, EIF3D, G3BP1, KHDRBS1, PGM3, ECHS1, LARS, TTC37, MLPH, PTAFR, THRAP3, SMARCC1, SECISBP2L, PEA15, OCIAD1, FTL, FXYD3, ARL1, PRPF8, CHD3, TOMM20, CLTA, RPS27A, SPOCK1, LIMA1, FAM213A, PGRMC1, TMEM245, SLC12A2, PPAPDC1B, AR, PUM1, DST, CREB3L4, CYC1, LPP, SLC30A4, ATP8B1, PDXDC1, TNFAIP2, U2SURP, S100A10, LIFR, PLEKHB2, CD2AP, CCT7, MYO1C, SSRP1, PTGES3, 6-Mar, ERP29, RAN, PTRF, DNAJB2, MDH2, TOB2, C11orf58, DAD1, BAG3, SNRPN, ZBTB38, HDAC1, PSMC5, IMPAD1, H1FX, PERP, CIB1, IDH1, MYO6, DMXL1, SEPW1, NONO, CAND1, IPO7, APEX1, SNHG8, PIK3R1, RAB3B, GALNT7, TBL1XR1, HEXB, SETD7, ALCAM, HSPA4, THOC2, MPC2, CCAR1, CYTIP, LGMN, BBX, CRTAP, SMDT1, DYNLL2, ACTB, ATXN7L3B, GSTP1, PDCD6IP, MLLT4, BCL6, ANAPC5, YBX3, HNRNPF, UQCRB, COPS6, SET, NEFH, WDR6, LAMB2, FOXA1, TOMM7, GBP1, USP14, GPX4, SPDEF, LUC7L3, RCAN3, 15-Sep, CPPED1, PSMB5, YWHAG, PNN, CD99, EPCAM, DOCK5, SUPT16H, HN1L, ZMYND11, TXNIP, TTC19, KLK4, RAC1, PSMD7, TSPYL1, DSG2, NUCB2, CLCN3, CD151, TRIP11, TSPAN3, SCD, COX8A, SORD, SEC13, PSMB7, DPP7, SLC3A2, NDUFB10, LONP2, GFPT1, RAB11A, ZNF331, RPL6, PSMB6, PTPN11, ERGIC3, TM9SF2, UQCRC1, KIF5C, NPC2, ATP1B3, TCEAL4, PRNP, DYNC1LI2, FKBP4, C1orf21, ODC1, IDH2, NME4, NOP58, RFC1, DDX1, ST6GAL1, NGFRAP1, ATP5C1, STK4, PSMA4, SNX9, CCND1, SLC38A1, BMPR2, PARP1, DHRS3, RPL23A, SRPR, FBL, SUMF2, PRDX4, ATP6V1G1, ARID5B, ACBD3, TMEM205, SKP1, CHCHD2, PDS5A, CAPN2, CHD9, ARSD, ENTPD5, PTPLAD1, AARS, PYGB, DNAJC10, NPDC1, ANP32B, ITM2C, UFL1, RAD50, PTPLB, ARFGEF2, TMEM141, HOMER2, COX6C, USP22, PHKB, RNH1, ALDH9A1, RTN3, SORL1, TUBB, PRPF40A, UHMK1, NUDT3, LARP1, AES, ID2, CSRP1, FGL2, GTF2I, FNDC3A, HLA-DPA1, NDUFS1, HMGN1, HSPB1, GADD45G, MTUS1, C12orf57, PLXNB2, TMED3, ATP5G2, EIF3I, GNPTAB, ALDH6A1, PPM1G, ATP5F1, CGNL1, RPL26, KARS, KCTD3, ITGB2, SEMA4D, LNPEP, GALNT1, MSRB1, MXD4, CLDN3, SEC11A, DHX15, PARM1, MESDC2, BOD1L1, NCAPD3, EIF3F, CANT1, RPL36, NET1, PHPT1, ABCF1, FUBP1, HNRNPA1, TMEM9, RPL28, NOP56, NDUFV1, VIMP, MAOA, SETD2, PLXNC1, DARS, SC5D, CCNG1, RBBP7, NBL1, KRT8, METTL7A, EIF2AK4, CLINT1, SPTAN1, MPP5, LIMCH1, MYO9A, TARS, SNRPD3, LMAN2, ITM2B, COMT, AC013394.2, CREB3L2, MEAF6, PFKL, TXN2, FOLH1, BCAP31, NEMF, PNKD, CYB5A, EIF3B, PPCS, HYOU1, ATXN10, ITGA6, TCF12, SOX4, C6orf89, ACACA, PGRMC2, LMNA, PSMC3, ERRFI1, NCKAP1, PABPC4, SEC22C, PSMC2, GUSB, BHLHE40, TMEM230, IFT57, M6PR, ARG2, NDUFB9, CBR1, DENND4C, PPM1K, TJP1, ATRAID, JMY, C1orf43, MRPL51, PPDPF, PPA1, EGFR, RPL7L1, RHOU, SYPL1, RNF7, SMAD4, NHP2L1, MT-ATP8, CNN3, POLR2B, NPY, UBE2Z, TRIM44, STRA13, RNF187, LRRC59, MRFAP1L1, KIAA1324, ARFGEF1, CITED2, SLC30A9, ZNF532, TMEM87B, CDC42BPB, EMP2, SMARCA1, ITGAV, HSD17B12, SCCPDH, ZMPSTE24, BACE2, FKBP3, SNHG5, CHD6, MGST3, DDI2, HTATSF1, MGA, ARHGAP6, CTTN, ITGB5, NDEL1, BMPR1B, NFIX, MBTPS1, AP1B1, GDF15, FASN, PPP1R7, AMOTL1, RDX, SMIM7, PSME4, STK39, SETD5, LRBA, EFNA1, FAT1, DNAJC21, GATA2, AKAP1, CDC37, TIA1, LGALS8, SLC25A4, SOCS2, CKAP5, STEAP1 |

**Table S4. The results of univariate Cox regression analysis for prognostic ECMGs.**

| **Genes** | **HR** | **HR.95L** | **HR.95H** | ***P*-value** |
| --- | --- | --- | --- | --- |
| ACPP | 0.767373 | 0.635931 | 0.925983 | 0.005742 |
| MSMB | 0.826235 | 0.722053 | 0.945448 | 0.005508 |
| TSPAN1 | 0.716216 | 0.554191 | 0.925610 | 0.010750 |
| GOLM1 | 0.616047 | 0.452792 | 0.838163 | 0.002044 |
| DHRS7 | 0.658773 | 0.445840 | 0.973402 | 0.036145 |
| KLK4 | 0.715688 | 0.514783 | 0.994999 | 0.046615 |
| CKB | 0.692094 | 0.496803 | 0.964152 | 0.029571 |
| DCXR | 0.681128 | 0.478315 | 0.969937 | 0.033237 |
| ALDH1A3 | 0.626729 | 0.443010 | 0.886636 | 0.008297 |
| CYB561 | 0.451880 | 0.258581 | 0.789678 | 0.005286 |
| P4HB | 0.534531 | 0.336647 | 0.848735 | 0.007926 |
| ADI1 | 0.397325 | 0.186097 | 0.848304 | 0.017075 |
| STEAP1 | 2.042101 | 1.245862 | 3.347221 | 0.004628 |
| CD9 | 0.526705 | 0.307638 | 0.901770 | 0.019448 |
| GRHL2 | 1.887840 | 1.019656 | 3.495237 | 0.043187 |
| PRSS8 | 0.744955 | 0.567192 | 0.978431 | 0.034283 |
| ANXA3 | 0.674685 | 0.483187 | 0.942077 | 0.020873 |
| BTG1 | 1.895001 | 1.018382 | 3.526211 | 0.043648 |
| NANS | 0.416860 | 0.249457 | 0.696602 | 0.000838 |
| ATP8B1 | 0.589784 | 0.373803 | 0.930556 | 0.023250 |
| MYBPC1 | 0.785152 | 0.649178 | 0.949606 | 0.012671 |
| EPHX2 | 0.409507 | 0.249459 | 0.672240 | 0.000415 |
| CNTNAP2 | 0.732037 | 0.552458 | 0.969988 | 0.029844 |
| DDAH1 | 0.353949 | 0.190260 | 0.658468 | 0.001041 |
| MYO6 | 1.428339 | 1.074789 | 1.898187 | 0.014009 |
| FAM3B | 0.796467 | 0.678697 | 0.934673 | 0.005312 |
| TRGC1 | 0.793576 | 0.639131 | 0.985342 | 0.036288 |
| SC5D | 0.509742 | 0.310521 | 0.836776 | 0.007708 |
| TMED3 | 0.435233 | 0.251260 | 0.753911 | 0.003000 |
| ALDH6A1 | 0.594145 | 0.358905 | 0.983570 | 0.042931 |
| SLC15A2 | 0.671865 | 0.508748 | 0.887282 | 0.005066 |
| CLGN | 0.707368 | 0.515606 | 0.970449 | 0.031882 |
| MAP3K8 | 1.835772 | 1.119729 | 3.009710 | 0.016027 |
| PTPRN2 | 0.668886 | 0.524267 | 0.853397 | 0.001215 |
| BACE2 | 0.596527 | 0.420119 | 0.847010 | 0.003874 |
| KIAA0319L | 0.403188 | 0.211351 | 0.769148 | 0.005843 |
| ARHGAP4 | 1.727571 | 1.092597 | 2.731567 | 0.019346 |
| QKI | 2.044038 | 1.054106 | 3.963635 | 0.034352 |
| SPARC | 1.491883 | 1.043920 | 2.132073 | 0.028099 |
| PDIA3 | 0.475925 | 0.266632 | 0.849503 | 0.012015 |
| CD38 | 0.739872 | 0.586420 | 0.933479 | 0.011072 |
| ST3GAL1 | 2.186336 | 1.430440 | 3.341675 | 0.000302 |
| REXO2 | 2.077577 | 1.375958 | 3.136959 | 0.000505 |
| CDKN1A | 0.688153 | 0.490972 | 0.964523 | 0.030033 |
| SYK | 1.980723 | 1.210837 | 3.240128 | 0.006492 |
| SLC20A1 | 2.901393 | 1.492500 | 5.640254 | 0.001686 |
| CD55 | 1.577697 | 1.050512 | 2.369442 | 0.027988 |
| ZFAND5 | 2.300889 | 1.173505 | 4.511347 | 0.015279 |
| CTSZ | 2.908855 | 1.432944 | 5.904932 | 0.003119 |
| FCGRT | 1.742283 | 1.031625 | 2.942493 | 0.037856 |
| OSBPL8 | 1.849873 | 1.186781 | 2.883454 | 0.006605 |

HR, hazard ratio

**Table S5. A total of 80 published signatures were retrieved from the literatures.**

| **Model** | **PMID** | **Type** | **Author** | **ENSEMBL** | **Coef** |
| --- | --- | --- | --- | --- | --- |
| Model-1 | 34336641 | mRNA | Huan Liu | ENSG00000196139 | 0.213 |
| Model-1 | 34336641 | mRNA | Huan Liu | ENSG00000179148 | 0.224 |
| Model-1 | 34336641 | mRNA | Huan Liu | ENSG00000154518 | 0.183 |
| Model-1 | 34336641 | mRNA | Huan Liu | ENSG00000110619 | 0.182 |
| Model-1 | 34336641 | mRNA | Huan Liu | ENSG00000125144 | -0.346 |
| Model-1 | 34336641 | mRNA | Huan Liu | ENSG00000073756 | -0.193 |
| Model-1 | 34336641 | mRNA | Huan Liu | ENSG00000072274 | 0.299 |
| Model-2 | 32923125 | mRNA | Ning Shao | ENSG00000181444 | 0.01224 |
| Model-2 | 32923125 | mRNA | Ning Shao | ENSG00000156463 | -0.00755 |
| Model-2 | 32923125 | mRNA | Ning Shao | ENSG00000139220 | 0.00802 |
| Model-2 | 32923125 | mRNA | Ning Shao | ENSG00000196132 | 0.01675 |
| Model-2 | 32923125 | mRNA | Ning Shao | ENSG00000135451 | 0.02335 |
| Model-2 | 32923125 | mRNA | Ning Shao | ENSG00000155265 | 0.03744 |
| Model-3 | 33121495 | mRNA | Jiangfeng Li | ENSG00000100353 | -0.021 |
| Model-3 | 33121495 | mRNA | Jiangfeng Li | ENSG00000161960 | 0.349 |
| Model-3 | 33121495 | mRNA | Jiangfeng Li | ENSG00000155506 | 0.036 |
| Model-4 | 35812443 | mRNA | Mingyi Ju | ENSG00000120833 | -0.332 |
| Model-4 | 35812443 | mRNA | Mingyi Ju | ENSG00000120738 | -0.111 |
| Model-4 | 35812443 | mRNA | Mingyi Ju | ENSG00000171848 | 0.286 |
| Model-4 | 35812443 | mRNA | Mingyi Ju | ENSG00000166340 | -0.609 |
| Model-4 | 35812443 | mRNA | Mingyi Ju | ENSG00000182919 | -0.748 |
| Model-5 | 35071228 | mRNA | Zhi-Bin Ke | ENSG00000070669 | 0.1615 |
| Model-5 | 35071228 | mRNA | Zhi-Bin Ke | ENSG00000166123 | -0.04635 |
| Model-5 | 35071228 | mRNA | Zhi-Bin Ke | ENSG00000116044 | -0.07099 |
| Model-5 | 35071228 | mRNA | Zhi-Bin Ke | ENSG00000171848 | 0.14414 |
| Model-6 | 35538543 | mRNA | Fang Lyu | ENSG00000187608 | 0.18158 |
| Model-6 | 35538543 | mRNA | Fang Lyu | ENSG00000128016 | -0.19711 |
| Model-7 | 32264916 | mRNA | Daixing Hu | ENSG00000083290 | 0.97225 |
| Model-7 | 32264916 | mRNA | Daixing Hu | ENSG00000091106 | -1.74297 |
| Model-7 | 32264916 | mRNA | Daixing Hu | ENSG00000100030 | -1.11799 |
| Model-7 | 32264916 | mRNA | Daixing Hu | ENSG00000130734 | -1.12182 |
| Model-7 | 32264916 | mRNA | Daixing Hu | ENSG00000102882 | -0.73348 |
| Model-7 | 32264916 | mRNA | Daixing Hu | ENSG00000110046 | 1.40252 |
| Model-7 | 32264916 | mRNA | Daixing Hu | ENSG00000181652 | -0.49364 |
| Model-7 | 32264916 | mRNA | Daixing Hu | ENSG00000150907 | -1.09886 |
| Model-7 | 32264916 | mRNA | Daixing Hu | ENSG00000171862 | -0.68955 |
| Model-7 | 32264916 | mRNA | Daixing Hu | ENSG00000094631 | 1.80095 |
| Model-7 | 32264916 | mRNA | Daixing Hu | ENSG00000185345 | -0.99993 |
| Model-7 | 32264916 | mRNA | Daixing Hu | ENSG00000152137 | 0.35846 |
| Model-7 | 32264916 | mRNA | Daixing Hu | ENSG00000185624 | -0.51552 |
| Model-7 | 32264916 | mRNA | Daixing Hu | ENSG00000076984 | 1.56551 |
| Model-7 | 32264916 | mRNA | Daixing Hu | ENSG00000198793 | -0.96348 |
| Model-7 | 32264916 | mRNA | Daixing Hu | ENSG00000106615 | 1.65516 |
| Model-7 | 32264916 | mRNA | Daixing Hu | ENSG00000165699 | 0.73934 |
| Model-7 | 32264916 | mRNA | Daixing Hu | ENSG00000089685 | 0.27799 |
| Model-7 | 32264916 | mRNA | Daixing Hu | ENSG00000171700 | 1.43484 |
| Model-7 | 32264916 | mRNA | Daixing Hu | ENSG00000169228 | -0.63037 |
| Model-7 | 32264916 | mRNA | Daixing Hu | ENSG00000101213 | -0.2858 |
| Model-7 | 32264916 | mRNA | Daixing Hu | ENSG00000158458 | -1.05312 |
| Model-8 | 34778244 | mRNA | Zhengtong Lv | ENSG00000042286 | 0.19867 |
| Model-8 | 34778244 | mRNA | Zhengtong Lv | ENSG00000187134 | 0.05148 |
| Model-8 | 34778244 | mRNA | Zhengtong Lv | ENSG00000151632 | 0.04941 |
| Model-8 | 34778244 | mRNA | Zhengtong Lv | ENSG00000160200 | 0.02658 |
| Model-8 | 34778244 | mRNA | Zhengtong Lv | ENSG00000144554 | 0.00202 |
| Model-8 | 34778244 | mRNA | Zhengtong Lv | ENSG00000167996 | 0.00027 |
| Model-8 | 34778244 | mRNA | Zhengtong Lv | ENSG00000160211 | 0.00189 |
| Model-8 | 34778244 | mRNA | Zhengtong Lv | ENSG00000244005 | 0.13708 |
| Model-8 | 34778244 | mRNA | Zhengtong Lv | ENSG00000105281 | -0.00602 |
| Model-9 | 35154101 | mRNA | Dechao Feng | ENSG00000169676 | 0.8917 |
| Model-9 | 35154101 | mRNA | Dechao Feng | ENSG00000258947 | 1.11416 |
| Model-9 | 35154101 | mRNA | Dechao Feng | ENSG00000183715 | 1.54641 |
| Model-9 | 35154101 | mRNA | Dechao Feng | ENSG00000100526 | 2.47249 |
| Model-9 | 35154101 | mRNA | Dechao Feng | ENSG00000070019 | 1.64994 |
| Model-9 | 35154101 | mRNA | Dechao Feng | ENSG00000163631 | 4.87246 |
| Model-9 | 35154101 | mRNA | Dechao Feng | ENSG00000154027 | 0.8905 |
| Model-9 | 35154101 | mRNA | Dechao Feng | ENSG00000153291 | 0.74639 |
| Model-9 | 35154101 | mRNA | Dechao Feng | ENSG00000123560 | 0.26291 |
| Model-9 | 35154101 | mRNA | Dechao Feng | ENSG00000073756 | 0.65871 |
| Model-10 | 35135561 | mRNA | Ding Hu | ENSG00000115163 | 0.741 |
| Model-10 | 35135561 | mRNA | Ding Hu | ENSG00000148346 | -0.134 |
| Model-10 | 35135561 | mRNA | Ding Hu | ENSG00000114270 | 0.802 |
| Model-10 | 35135561 | mRNA | Ding Hu | ENSG00000163631 | 0.222 |
| Model-10 | 35135561 | mRNA | Ding Hu | ENSG00000162543 | -0.61 |
| Model-10 | 35135561 | mRNA | Ding Hu | ENSG00000164299 | 0.302 |
| Model-10 | 35135561 | mRNA | Ding Hu | ENSG00000111319 | -0.227 |
| Model-10 | 35135561 | mRNA | Ding Hu | ENSG00000160180 | -0.111 |
| Model-11 | 37011878 | mRNA | Bisheng Cheng | ENSG00000123191 | -0.1626 |
| Model-11 | 37011878 | mRNA | Bisheng Cheng | ENSG00000137992 | -0.1323 |
| Model-11 | 37011878 | mRNA | Bisheng Cheng | ENSG00000144182 | 0.0425 |
| Model-11 | 37011878 | mRNA | Bisheng Cheng | ENSG00000131828 | 0.9631 |
| Model-12 | 36439479 | mRNA | Aoyu Fan | ENSG00000144218 | -0.1028 |
| Model-12 | 36439479 | mRNA | Aoyu Fan | ENSG00000182272 | 0.2922 |
| Model-12 | 36439479 | mRNA | Aoyu Fan | ENSG00000004468 | -0.511 |
| Model-12 | 36439479 | mRNA | Aoyu Fan | ENSG00000120903 | 0.0072 |
| Model-12 | 36439479 | mRNA | Aoyu Fan | ENSG00000170369 | 0.03832 |
| Model-12 | 36439479 | mRNA | Aoyu Fan | ENSG00000069122 | 0.2041 |
| Model-12 | 36439479 | mRNA | Aoyu Fan | ENSG00000129437 | 0.1326 |
| Model-12 | 36439479 | mRNA | Aoyu Fan | ENSG00000114248 | 0.1493 |
| Model-12 | 36439479 | mRNA | Aoyu Fan | ENSG00000198417 | -0.1262 |
| Model-12 | 36439479 | mRNA | Aoyu Fan | ENSG00000125144 | -0.0116 |
| Model-12 | 36439479 | mRNA | Aoyu Fan | ENSG00000185303 | -0.1639 |
| Model-12 | 36439479 | mRNA | Aoyu Fan | ENSG00000099960 | -0.1697 |
| Model-12 | 36439479 | mRNA | Aoyu Fan | ENSG00000095627 | -0.4706 |
| Model-13 | 35603206 | mRNA | Zhipeng Xu | ENSG00000139182 | 0.10051 |
| Model-13 | 35603206 | mRNA | Zhipeng Xu | ENSG00000136295 | 0.31415 |
| Model-13 | 35603206 | mRNA | Zhipeng Xu | ENSG00000138074 | 0.12448 |
| Model-13 | 35603206 | mRNA | Zhipeng Xu | ENSG00000124181 | 0.1217 |
| Model-13 | 35603206 | mRNA | Zhipeng Xu | ENSG00000161013 | 0.12769 |
| Model-13 | 35603206 | mRNA | Zhipeng Xu | ENSG00000214078 | 0.3431 |
| Model-13 | 35603206 | mRNA | Zhipeng Xu | ENSG00000091164 | -0.36546 |
| Model-13 | 35603206 | mRNA | Zhipeng Xu | ENSG00000014641 | -0.15001 |
| Model-13 | 35603206 | mRNA | Zhipeng Xu | ENSG00000117461 | 0.26431 |
| Model-13 | 35603206 | mRNA | Zhipeng Xu | ENSG00000145781 | -0.10354 |
| Model-13 | 35603206 | mRNA | Zhipeng Xu | ENSG00000156858 | 0.72799 |
| Model-13 | 35603206 | mRNA | Zhipeng Xu | ENSG00000166927 | 0.29512 |
| Model-13 | 35603206 | mRNA | Zhipeng Xu | ENSG00000144645 | 0.01485 |
| Model-13 | 35603206 | mRNA | Zhipeng Xu | ENSG00000113328 | -0.16758 |
| Model-13 | 35603206 | mRNA | Zhipeng Xu | ENSG00000131188 | 0.00185 |
| Model-13 | 35603206 | mRNA | Zhipeng Xu | ENSG00000154719 | -0.10157 |
| Model-13 | 35603206 | mRNA | Zhipeng Xu | ENSG00000071246 | 0.24395 |
| Model-13 | 35603206 | mRNA | Zhipeng Xu | ENSG00000137947 | -0.90056 |
| Model-13 | 35603206 | mRNA | Zhipeng Xu | ENSG00000163762 | 0.01626 |
| Model-13 | 35603206 | mRNA | Zhipeng Xu | ENSG00000102878 | 0.06814 |
| Model-13 | 35603206 | mRNA | Zhipeng Xu | ENSG00000156299 | 0.02135 |
| Model-13 | 35603206 | mRNA | Zhipeng Xu | ENSG00000198753 | 0.14298 |
| Model-13 | 35603206 | mRNA | Zhipeng Xu | ENSG00000165572 | -0.12124 |
| Model-13 | 35603206 | mRNA | Zhipeng Xu | ENSG00000095970 | 0.12977 |
| Model-13 | 35603206 | mRNA | Zhipeng Xu | ENSG00000166340 | -0.76238 |
| Model-13 | 35603206 | mRNA | Zhipeng Xu | ENSG00000129538 | 0.25465 |
| Model-13 | 35603206 | mRNA | Zhipeng Xu | ENSG00000164105 | 0.21889 |
| Model-13 | 35603206 | mRNA | Zhipeng Xu | ENSG00000232995 | 0.01354 |
| Model-13 | 35603206 | mRNA | Zhipeng Xu | ENSG00000068400 | 0.05148 |
| Model-13 | 35603206 | mRNA | Zhipeng Xu | ENSG00000100292 | 0.01429 |
| Model-13 | 35603206 | mRNA | Zhipeng Xu | ENSG00000102302 | 0.03252 |
| Model-13 | 35603206 | mRNA | Zhipeng Xu | ENSG00000116663 | 0.14687 |
| Model-13 | 35603206 | mRNA | Zhipeng Xu | ENSG00000104267 | 0.00917 |
| Model-14 | 35836112 | mRNA | Cheng-Yuan Gu | ENSG00000160293 | 3.3924 |
| Model-14 | 35836112 | mRNA | Cheng-Yuan Gu | ENSG00000099250 | 0.5622 |
| Model-14 | 35836112 | mRNA | Cheng-Yuan Gu | ENSG00000186652 | 1.1902 |
| Model-14 | 35836112 | mRNA | Cheng-Yuan Gu | ENSG00000184916 | 0.3202 |
| Model-14 | 35836112 | mRNA | Cheng-Yuan Gu | ENSG00000175445 | -0.0801 |
| Model-14 | 35836112 | mRNA | Cheng-Yuan Gu | ENSG00000133110 | 0.3631 |
| Model-14 | 35836112 | mRNA | Cheng-Yuan Gu | ENSG00000163430 | 0.7902 |
| Model-14 | 35836112 | mRNA | Cheng-Yuan Gu | ENSG00000163737 | 0.6997 |
| Model-14 | 35836112 | mRNA | Cheng-Yuan Gu | ENSG00000101384 | 0.2249 |
| Model-14 | 35836112 | mRNA | Cheng-Yuan Gu | ENSG00000168542 | 0.3681 |
| Model-14 | 35836112 | mRNA | Cheng-Yuan Gu | ENSG00000173391 | -0.0198 |
| Model-14 | 35836112 | mRNA | Cheng-Yuan Gu | ENSG00000112715 | 0.1602 |
| Model-14 | 35836112 | mRNA | Cheng-Yuan Gu | ENSG00000109072 | 0.0476 |
| Model-14 | 35836112 | mRNA | Cheng-Yuan Gu | ENSG00000196154 | 0.9752 |
| Model-14 | 35836112 | mRNA | Cheng-Yuan Gu | ENSG00000077782 | -1.1699 |
| Model-14 | 35836112 | mRNA | Cheng-Yuan Gu | ENSG00000163956 | 1.1291 |
| Model-14 | 35836112 | mRNA | Cheng-Yuan Gu | ENSG00000124875 | -0.1911 |
| Model-14 | 35836112 | mRNA | Cheng-Yuan Gu | ENSG00000188488 | -0.4216 |
| Model-14 | 35836112 | mRNA | Cheng-Yuan Gu | ENSG00000142192 | -0.3995 |
| Model-15 | 36275733 | mRNA | Lei Chen | ENSG00000145824 | 0.18502 |
| Model-15 | 36275733 | mRNA | Lei Chen | ENSG00000115009 | -0.40887 |
| Model-15 | 36275733 | mRNA | Lei Chen | ENSG00000106178 | 0.28065 |
| Model-15 | 36275733 | mRNA | Lei Chen | ENSG00000006606 | 0.33033 |
| Model-16 | 36276080 | mRNA | Wangli Mei | ENSG00000141480 | 0.061 |
| Model-16 | 36276080 | mRNA | Wangli Mei | ENSG00000106105 | -0.01 |
| Model-16 | 36276080 | mRNA | Wangli Mei | ENSG00000136811 | 0.048 |
| Model-16 | 36276080 | mRNA | Wangli Mei | ENSG00000102144 | 0.012 |
| Model-16 | 36276080 | mRNA | Wangli Mei | ENSG00000067225 | 0.008 |
| Model-16 | 36276080 | mRNA | Wangli Mei | ENSG00000106397 | 0.057 |
| Model-16 | 36276080 | mRNA | Wangli Mei | ENSG00000100941 | 0.034 |
| Model-16 | 36276080 | mRNA | Wangli Mei | ENSG00000025708 | 0.034 |
| Model-16 | 36276080 | mRNA | Wangli Mei | ENSG00000196363 | 0.075 |
| Model-17 | 37255653 | mRNA | Yijun He | ENSG00000145198 | 0.21342 |
| Model-17 | 37255653 | mRNA | Yijun He | ENSG00000198576 | -0.30113 |
| Model-17 | 37255653 | mRNA | Yijun He | ENSG00000176887 | 0.17896 |
| Model-17 | 37255653 | mRNA | Yijun He | ENSG00000257335 | 0.22222 |
| Model-17 | 37255653 | mRNA | Yijun He | ENSG00000139445 | 0.18263 |
| Model-17 | 37255653 | mRNA | Yijun He | ENSG00000185686 | 0.08836 |
| Model-17 | 37255653 | mRNA | Yijun He | ENSG00000167346 | -0.12725 |
| Model-18 | 36471446 | mRNA | Cheng Yang | ENSG00000123080 | 0.12746 |
| Model-18 | 36471446 | mRNA | Cheng Yang | ENSG00000100526 | 0.17848 |
| Model-18 | 36471446 | mRNA | Cheng Yang | ENSG00000161800 | 0.5817 |
| Model-18 | 36471446 | mRNA | Cheng Yang | ENSG00000171848 | 0.11571 |
| Model-19 | 35813821 | mRNA | Miaomiao Wang | ENSG00000168685 | -0.46858 |
| Model-19 | 35813821 | mRNA | Miaomiao Wang | ENSG00000169436 | -0.31426 |
| Model-19 | 35813821 | mRNA | Miaomiao Wang | ENSG00000147394 | -0.61398 |
| Model-19 | 35813821 | mRNA | Miaomiao Wang | ENSG00000073756 | 0.21908 |
| Model-19 | 35813821 | mRNA | Miaomiao Wang | ENSG00000138356 | -0.31853 |
| Model-19 | 35813821 | mRNA | Miaomiao Wang | ENSG00000277893 | -0.6888 |
| Model-19 | 35813821 | mRNA | Miaomiao Wang | ENSG00000163347 | 0.28493 |
| Model-19 | 35813821 | mRNA | Miaomiao Wang | ENSG00000277209 | 0.21887 |
| Model-20 | 36386841 | mRNA | Xi Xiao | ENSG00000164695 | 0.2985 |
| Model-20 | 36386841 | mRNA | Xi Xiao | ENSG00000172115 | 0.5625 |
| Model-20 | 36386841 | mRNA | Xi Xiao | ENSG00000167468 | 0.6243 |
| Model-20 | 36386841 | mRNA | Xi Xiao | ENSG00000073605 | 0.3102 |
| Model-20 | 36386841 | mRNA | Xi Xiao | ENSG00000167207 | 1.0209 |
| Model-20 | 36386841 | mRNA | Xi Xiao | ENSG00000124181 | 0.9242 |
| Model-21 | 36246902 | mRNA | Yong Luo | ENSG00000114859 | 0.79 |
| Model-21 | 36246902 | mRNA | Yong Luo | ENSG00000011021 | -0.136 |
| Model-22 | 36561322 | lncRNA | Shaoqin Jiang | ENSG00000249898 | -1.53858 |
| Model-22 | 36561322 | lncRNA | Shaoqin Jiang | ENSG00000226200 | -0.90512 |
| Model-22 | 36561322 | lncRNA | Shaoqin Jiang | ENSG00000255717 | 1.50997 |
| Model-22 | 36561322 | lncRNA | Shaoqin Jiang | ENSG00000221953 | 1.41525 |
| Model-22 | 36561322 | lncRNA | Shaoqin Jiang | ENSG00000180525 | -1.05076 |
| Model-22 | 36561322 | lncRNA | Shaoqin Jiang | ENSG00000213904 | -1.29481 |
| Model-22 | 36561322 | lncRNA | Shaoqin Jiang | ENSG00000180539 | 0.78346 |
| Model-23 | 36092848 | mRNA | Bo-Yu Yang | ENSG00000085978 | 0.3201 |
| Model-23 | 36092848 | mRNA | Bo-Yu Yang | ENSG00000151632 | 0.75803 |
| Model-23 | 36092848 | mRNA | Bo-Yu Yang | ENSG00000100253 | 0.7616 |
| Model-23 | 36092848 | mRNA | Bo-Yu Yang | ENSG00000108839 | 2.77525 |
| Model-23 | 36092848 | mRNA | Bo-Yu Yang | ENSG00000069696 | 0.97253 |
| Model-23 | 36092848 | mRNA | Bo-Yu Yang | ENSG00000138413 | 0.07453 |
| Model-23 | 36092848 | mRNA | Bo-Yu Yang | ENSG00000173039 | 1.74889 |
| Model-23 | 36092848 | mRNA | Bo-Yu Yang | ENSG00000176108 | -1.10159 |
| Model-24 | 36124593 | mRNA | Yong Luo | ENSG00000159388 | -0.347 |
| Model-24 | 36124593 | mRNA | Yong Luo | ENSG00000006210 | -0.183 |
| Model-24 | 36124593 | mRNA | Yong Luo | ENSG00000123700 | 0.646 |
| Model-24 | 36124593 | mRNA | Yong Luo | ENSG00000173039 | 1.171 |
| Model-25 | 36324578 | mRNA | Yingxin Cai | ENSG00000197894 | -0.04662 |
| Model-25 | 36324578 | mRNA | Yingxin Cai | ENSG00000106546 | 0.03228 |
| Model-25 | 36324578 | mRNA | Yingxin Cai | ENSG00000140284 | -0.13818 |
| Model-25 | 36324578 | mRNA | Yingxin Cai | ENSG00000277893 | -0.20324 |
| Model-25 | 36324578 | mRNA | Yingxin Cai | ENSG00000104325 | 0.05072 |
| Model-25 | 36324578 | mRNA | Yingxin Cai | ENSG00000114200 | 0.35147 |
| Model-26 | 36589680 | mRNA | Cheng-Gong Luo | ENSG00000197548 | 0.76259 |
| Model-26 | 36589680 | mRNA | Cheng-Gong Luo | ENSG00000131165 | -0.05064 |
| Model-26 | 36589680 | mRNA | Cheng-Gong Luo | ENSG00000094631 | 0.35512 |
| Model-26 | 36589680 | mRNA | Cheng-Gong Luo | ENSG00000125347 | -0.11582 |
| Model-26 | 36589680 | mRNA | Cheng-Gong Luo | ENSG00000126456 | 0.10545 |
| Model-26 | 36589680 | mRNA | Cheng-Gong Luo | ENSG00000159714 | -0.15433 |
| Model-27 | 36717792 | lncRNA | Xiaofeng Cheng | ENSG00000105426 | -1.202 |
| Model-27 | 36717792 | lncRNA | Xiaofeng Cheng | ENSG00000130167 | -1.872 |
| Model-27 | 36717792 | lncRNA | Xiaofeng Cheng | ENSG00000162604 | 1.528 |
| Model-27 | 36717792 | lncRNA | Xiaofeng Cheng | ENSG00000203635 | -1.027 |
| Model-27 | 36717792 | lncRNA | Xiaofeng Cheng | ENSG00000213904 | -2.658 |
| Model-27 | 36717792 | lncRNA | Xiaofeng Cheng | ENSG00000275549 | -1.517 |
| Model-28 | 32782607 | mRNA | Xiangkun Wu | ENSG00000124157 | -0.16 |
| Model-28 | 32782607 | mRNA | Xiangkun Wu | ENSG00000153822 | -0.25 |
| Model-28 | 32782607 | mRNA | Xiangkun Wu | ENSG00000008196 | -0.321 |
| Model-28 | 32782607 | mRNA | Xiangkun Wu | ENSG00000171772 | -0.14 |
| Model-28 | 32782607 | mRNA | Xiangkun Wu | ENSG00000215262 | -0.23 |
| Model-28 | 32782607 | mRNA | Xiangkun Wu | ENSG00000081051 | 0.19 |
| Model-28 | 32782607 | mRNA | Xiangkun Wu | ENSG00000123201 | 0.17 |
| Model-28 | 32782607 | mRNA | Xiangkun Wu | ENSG00000152578 | 0.2 |
| Model-28 | 32782607 | mRNA | Xiangkun Wu | ENSG00000122584 | 0.22 |
| Model-28 | 32782607 | mRNA | Xiangkun Wu | ENSG00000176887 | 0.3 |
| Model-29 | 34484299 | mRNA | Zhihao Zou | ENSG00000073756 | -0.4619 |
| Model-29 | 34484299 | mRNA | Zhihao Zou | ENSG00000058272 | -0.141 |
| Model-29 | 34484299 | mRNA | Zhihao Zou | ENSG00000135318 | -0.2915 |
| Model-29 | 34484299 | mRNA | Zhihao Zou | ENSG00000138356 | -0.6051 |
| Model-29 | 34484299 | mRNA | Zhihao Zou | ENSG00000006625 | 0.3527 |
| Model-30 | 32882325 | mRNA | Lei Gao | ENSG00000135097 | 0.294 |
| Model-30 | 32882325 | mRNA | Lei Gao | ENSG00000139793 | -0.497 |
| Model-30 | 32882325 | mRNA | Lei Gao | ENSG00000275183 | -0.278 |
| Model-30 | 32882325 | mRNA | Lei Gao | ENSG00000076043 | 0.544 |
| Model-30 | 32882325 | mRNA | Lei Gao | ENSG00000129538 | 0.76 |
| Model-30 | 32882325 | mRNA | Lei Gao | ENSG00000101104 | 0.366 |
| Model-31 | 34136527 | mRNA | Daojun Lv | ENSG00000044115 | -2.771 |
| Model-31 | 34136527 | mRNA | Daojun Lv | ENSG00000141867 | 1.577 |
| Model-31 | 34136527 | mRNA | Daojun Lv | ENSG00000116288 | -2.239 |
| Model-31 | 34136527 | mRNA | Daojun Lv | ENSG00000170365 | 2.152 |
| Model-31 | 34136527 | mRNA | Daojun Lv | ENSG00000065978 | 2.428 |
| Model-32 | 33850736 | mRNA | Jiaochen Luan | ENSG00000006747 | -0.92247 |
| Model-32 | 33850736 | mRNA | Jiaochen Luan | ENSG00000154096 | 2.54278 |
| Model-32 | 33850736 | mRNA | Jiaochen Luan | ENSG00000184058 | 1.13792 |
| Model-32 | 33850736 | mRNA | Jiaochen Luan | ENSG00000204301 | 1.45694 |
| Model-32 | 33850736 | mRNA | Jiaochen Luan | ENSG00000172005 | -1.45727 |
| Model-32 | 33850736 | mRNA | Jiaochen Luan | ENSG00000104765 | -3.29561 |
| Model-33 | 33778002 | mRNA | Gongwei Long | ENSG00000122678 | 0.9139 |
| Model-33 | 33778002 | mRNA | Gongwei Long | ENSG00000136159 | -0.7278 |
| Model-33 | 33778002 | mRNA | Gongwei Long | ENSG00000181026 | -0.6761 |
| Model-33 | 33778002 | mRNA | Gongwei Long | ENSG00000163312 | -1.2567 |
| Model-34 | 34285549 | mRNA | Jiarong Cai | ENSG00000214548 | 0.2901 |
| Model-34 | 34285549 | mRNA | Jiarong Cai | ENSG00000137673 | 0.4701 |
| Model-34 | 34285549 | mRNA | Jiarong Cai | ENSG00000176463 | 0.4693 |
| Model-34 | 34285549 | mRNA | Jiarong Cai | ENSG00000162975 | 0.38 |
| Model-34 | 34285549 | mRNA | Jiarong Cai | ENSG00000133111 | 0.3384 |
| Model-34 | 34285549 | mRNA | Jiarong Cai | ENSG00000140538 | 0.2656 |
| Model-34 | 34285549 | lncRNA | Jiarong Cai | ENSG00000230366 | -0.0112 |
| Model-34 | 34285549 | lncRNA | Jiarong Cai | ENSG00000206337 | -0.1128 |
| Model-34 | 34285549 | mRNA | Jiarong Cai | ENSG00000134369 | -0.1493 |
| Model-34 | 34285549 | mRNA | Jiarong Cai | ENSG00000106031 | -0.2229 |
| Model-34 | 34285549 | mRNA | Jiarong Cai | ENSG00000170961 | -0.5077 |
| Model-34 | 34285549 | mRNA | Jiarong Cai | ENSG00000173894 | -0.6602 |
| Model-34 | 34285549 | mRNA | Jiarong Cai | ENSG00000276368 | -1.0755 |
| Model-34 | 34285549 | mRNA | Jiarong Cai | ENSG00000114520 | -2.3318 |
| Model-35 | 36891298 | mRNA | Chenglin Han | ENSG00000173137 | 0.43696 |
| Model-35 | 36891298 | mRNA | Chenglin Han | ENSG00000151929 | -0.16472 |
| Model-35 | 36891298 | mRNA | Chenglin Han | ENSG00000115163 | 0.04647 |
| Model-35 | 36891298 | mRNA | Chenglin Han | ENSG00000111206 | 0.44738 |
| Model-35 | 36891298 | mRNA | Chenglin Han | ENSG00000131981 | -0.07335 |
| Model-35 | 36891298 | mRNA | Chenglin Han | ENSG00000188130 | 0.09476 |
| Model-35 | 36891298 | mRNA | Chenglin Han | ENSG00000086991 | 0.48953 |
| Model-35 | 36891298 | mRNA | Chenglin Han | ENSG00000090447 | 0.121 |
| Model-36 | 37193176 | mRNA | Yibing Wang | ENSG00000171791 | -0.02523 |
| Model-36 | 37193176 | mRNA | Yibing Wang | ENSG00000153094 | 0.351 |
| Model-36 | 37193176 | mRNA | Yibing Wang | ENSG00000176171 | 0.10951 |
| Model-36 | 37193176 | mRNA | Yibing Wang | ENSG00000064012 | 0.16747 |
| Model-36 | 37193176 | mRNA | Yibing Wang | ENSG00000083799 | -0.5032 |
| Model-36 | 37193176 | mRNA | Yibing Wang | ENSG00000048052 | 0.21125 |
| Model-36 | 37193176 | mRNA | Yibing Wang | ENSG00000182054 | -0.64897 |
| Model-36 | 37193176 | mRNA | Yibing Wang | ENSG00000151151 | -0.41843 |
| Model-36 | 37193176 | mRNA | Yibing Wang | ENSG00000136997 | 0.05921 |
| Model-36 | 37193176 | mRNA | Yibing Wang | ENSG00000166851 | 0.361 |
| Model-36 | 37193176 | mRNA | Yibing Wang | ENSG00000232810 | -0.11898 |
| Model-36 | 37193176 | mRNA | Yibing Wang | ENSG00000067182 | -0.05462 |
| Model-36 | 37193176 | mRNA | Yibing Wang | ENSG00000165699 | 0.10101 |
| Model-37 | 33101546 | mRNA | Bide Liu | ENSG00000116985 | 0.245 |
| Model-37 | 33101546 | mRNA | Bide Liu | ENSG00000145919 | 0.63 |
| Model-37 | 33101546 | mRNA | Bide Liu | ENSG00000178585 | 0.446 |
| Model-37 | 33101546 | mRNA | Bide Liu | ENSG00000163251 | -0.594 |
| Model-37 | 33101546 | mRNA | Bide Liu | ENSG00000166923 | 0.207 |
| Model-37 | 33101546 | mRNA | Bide Liu | ENSG00000150457 | -0.265 |
| Model-37 | 33101546 | mRNA | Bide Liu | ENSG00000105835 | 0.349 |
| Model-37 | 33101546 | mRNA | Bide Liu | ENSG00000142875 | -0.263 |
| Model-37 | 33101546 | mRNA | Bide Liu | ENSG00000124232 | 0.342 |
| Model-37 | 33101546 | mRNA | Bide Liu | ENSG00000071537 | -0.076 |
| Model-37 | 33101546 | mRNA | Bide Liu | ENSG00000163482 | 0.825 |
| Model-37 | 33101546 | mRNA | Bide Liu | ENSG00000125878 | 0.05 |
| Model-37 | 33101546 | mRNA | Bide Liu | ENSG00000162552 | 0.287 |
| Model-38 | 36312753 | mRNA | Ming Yang | ENSG00000106462 | 0.46 |
| Model-38 | 36312753 | mRNA | Ming Yang | ENSG00000128886 | -0.18 |
| Model-38 | 36312753 | mRNA | Ming Yang | ENSG00000130208 | -0.04 |
| Model-38 | 36312753 | mRNA | Ming Yang | ENSG00000239672 | -0.22 |
| Model-38 | 36312753 | mRNA | Ming Yang | ENSG00000139438 | -0.6 |
| Model-38 | 36312753 | mRNA | Ming Yang | ENSG00000149150 | -0.71 |
| Model-38 | 36312753 | mRNA | Ming Yang | ENSG00000187210 | -0.04 |
| Model-38 | 36312753 | mRNA | Ming Yang | ENSG00000251493 | 0.17 |
| Model-38 | 36312753 | mRNA | Ming Yang | ENSG00000139219 | 0.028 |
| Model-38 | 36312753 | mRNA | Ming Yang | ENSG00000176153 | -0.11 |
| Model-38 | 36312753 | mRNA | Ming Yang | ENSG00000164379 | 0.08 |
| Model-38 | 36312753 | mRNA | Ming Yang | ENSG00000172201 | -0.32 |
| Model-38 | 36312753 | mRNA | Ming Yang | ENSG00000137331 | -0.17 |
| Model-38 | 36312753 | mRNA | Ming Yang | ENSG00000127990 | -0.13 |
| Model-38 | 36312753 | mRNA | Ming Yang | ENSG00000171714 | -0.25 |
| Model-38 | 36312753 | mRNA | Ming Yang | ENSG00000269190 | -0.03 |
| Model-38 | 36312753 | mRNA | Ming Yang | ENSG00000182013 | 0.49 |
| Model-38 | 36312753 | mRNA | Ming Yang | ENSG00000124205 | -0.03 |
| Model-39 | 36498737 | mRNA | Yiliyasi Yimamu | ENSG00000106483 | 0.06447 |
| Model-39 | 36498737 | mRNA | Yiliyasi Yimamu | ENSG00000163497 | -0.06196 |
| Model-39 | 36498737 | mRNA | Yiliyasi Yimamu | ENSG00000108821 | 0.37184 |
| Model-39 | 36498737 | mRNA | Yiliyasi Yimamu | ENSG00000137573 | 0.06892 |
| Model-40 | 35111198 | mRNA | Guoda Song | ENSG00000177479 | 1.3876 |
| Model-40 | 35111198 | mRNA | Guoda Song | ENSG00000116663 | 0.7596 |
| Model-40 | 35111198 | mRNA | Guoda Song | ENSG00000114450 | 0.5102 |
| Model-40 | 35111198 | mRNA | Guoda Song | ENSG00000138411 | 1.5888 |
| Model-40 | 35111198 | mRNA | Guoda Song | ENSG00000099949 | 1.5015 |
| Model-40 | 35111198 | mRNA | Guoda Song | ENSG00000138942 | -2.0379 |
| Model-41 | 37122696 | mRNA | Weian Zhu | ENSG00000101160 | 0.411 |
| Model-41 | 37122696 | mRNA | Weian Zhu | ENSG00000104870 | 0.025 |
| Model-41 | 37122696 | mRNA | Weian Zhu | ENSG00000135052 | -0.161 |
| Model-41 | 37122696 | mRNA | Weian Zhu | ENSG00000267795 | -0.107 |
| Model-41 | 37122696 | mRNA | Weian Zhu | ENSG00000014257 | -0.012 |
| Model-41 | 37122696 | mRNA | Weian Zhu | ENSG00000183844 | -0.029 |
| Model-41 | 37122696 | mRNA | Weian Zhu | ENSG00000160180 | -0.056 |
| Model-41 | 37122696 | mRNA | Weian Zhu | ENSG00000225937 | -0.025 |
| Model-41 | 37122696 | mRNA | Weian Zhu | ENSG00000263639 | -0.08 |
| Model-42 | 36033512 | mRNA | Zhuofan Mou | ENSG00000169564 | -0.508 |
| Model-42 | 36033512 | mRNA | Zhuofan Mou | ENSG00000100836 | 1.026 |
| Model-42 | 36033512 | mRNA | Zhuofan Mou | ENSG00000142949 | 0.363 |
| Model-42 | 36033512 | mRNA | Zhuofan Mou | ENSG00000226950 | -0.567 |
| Model-42 | 36033512 | mRNA | Zhuofan Mou | ENSG00000136997 | 0.372 |
| Model-43 | 32669958 | mRNA | Xinyu Geng | ENSG00000196611 | -0.04703 |
| Model-43 | 32669958 | mRNA | Xinyu Geng | ENSG00000137673 | -0.0223 |
| Model-43 | 32669958 | mRNA | Xinyu Geng | ENSG00000099953 | 0.33655 |
| Model-43 | 32669958 | mRNA | Xinyu Geng | ENSG00000125966 | 0.21821 |
| Model-43 | 32669958 | mRNA | Xinyu Geng | ENSG00000167346 | -0.11865 |
| Model-44 | 37007952 | mRNA | Lin-Ying Xie | ENSG00000103496 | 0.891 |
| Model-44 | 37007952 | mRNA | Lin-Ying Xie | ENSG00000108106 | 0.618 |
| Model-44 | 37007952 | mRNA | Lin-Ying Xie | ENSG00000127774 | 0.427 |
| Model-44 | 37007952 | mRNA | Lin-Ying Xie | ENSG00000102119 | 0.694 |
| Model-44 | 37007952 | mRNA | Lin-Ying Xie | ENSG00000104805 | -0.628 |
| Model-44 | 37007952 | mRNA | Lin-Ying Xie | ENSG00000100116 | -0.632 |
| Model-45 | 33816226 | mRNA | Xiaohan Ren | ENSG00000167332 | -0.07 |
| Model-45 | 33816226 | mRNA | Xiaohan Ren | ENSG00000107317 | -0.12 |
| Model-45 | 33816226 | mRNA | Xiaohan Ren | ENSG00000134240 | -0.1 |
| Model-45 | 33816226 | mRNA | Xiaohan Ren | ENSG00000125780 | -0.16 |
| Model-45 | 33816226 | mRNA | Xiaohan Ren | ENSG00000131781 | -0.31 |
| Model-45 | 33816226 | mRNA | Xiaohan Ren | ENSG00000204262 | 0.2 |
| Model-45 | 33816226 | mRNA | Xiaohan Ren | ENSG00000117399 | 0.23 |
| Model-46 | 34267780 | mRNA | Shuaishuai Fan | ENSG00000181085 | 0.18634 |
| Model-46 | 34267780 | mRNA | Shuaishuai Fan | ENSG00000181444 | 0.52337 |
| Model-46 | 34267780 | mRNA | Shuaishuai Fan | ENSG00000258839 | 0.6245 |
| Model-47 | 35246070 | mRNA | Yiqiao Zhao | ENSG00000118298 | -0.02821 |
| Model-47 | 35246070 | mRNA | Yiqiao Zhao | ENSG00000121207 | -0.04758 |
| Model-47 | 35246070 | mRNA | Yiqiao Zhao | ENSG00000167889 | 0.04194 |
| Model-48 | 33995635 | mRNA | Qijie Zhang | ENSG00000008300 | 0.0312 |
| Model-48 | 33995635 | mRNA | Qijie Zhang | ENSG00000205212 | 0.0571 |
| Model-48 | 33995635 | mRNA | Qijie Zhang | ENSG00000066230 | 0.0356 |
| Model-48 | 33995635 | mRNA | Qijie Zhang | ENSG00000129437 | 0.0026 |
| Model-48 | 33995635 | mRNA | Qijie Zhang | ENSG00000205861 | -0.0031 |
| Model-48 | 33995635 | mRNA | Qijie Zhang | ENSG00000116745 | -0.0034 |
| Model-48 | 33995635 | mRNA | Qijie Zhang | ENSG00000099960 | -0.0533 |
| Model-48 | 33995635 | mRNA | Qijie Zhang | ENSG00000182459 | 0.6825 |
| Model-48 | 33995635 | mRNA | Qijie Zhang | ENSG00000254726 | 0.0178 |
| Model-48 | 33995635 | mRNA | Qijie Zhang | ENSG00000182472 | 0.1853 |
| Model-48 | 33995635 | mRNA | Qijie Zhang | ENSG00000167281 | -0.0688 |
| Model-49 | 36106334 | mRNA | Ying Zhang | ENSG00000073756 | -0.033 |
| Model-49 | 36106334 | mRNA | Ying Zhang | ENSG00000163082 | 0.188 |
| Model-49 | 36106334 | mRNA | Ying Zhang | ENSG00000163631 | 0.149 |
| Model-49 | 36106334 | mRNA | Ying Zhang | ENSG00000188257 | -0.045 |
| Model-49 | 36106334 | mRNA | Ying Zhang | ENSG00000277893 | -0.229 |
| Model-49 | 36106334 | mRNA | Ying Zhang | ENSG00000181856 | -0.035 |
| Model-50 | 34419719 | mRNA | Qianwei Xing | ENSG00000121486 | -0.32299 |
| Model-50 | 34419719 | mRNA | Qianwei Xing | ENSG00000120688 | -0.30281 |
| Model-50 | 34419719 | mRNA | Qianwei Xing | ENSG00000076770 | -0.47751 |
| Model-50 | 34419719 | mRNA | Qianwei Xing | ENSG00000120693 | -0.08715 |
| Model-50 | 34419719 | mRNA | Qianwei Xing | ENSG00000179299 | -0.13817 |
| Model-50 | 34419719 | mRNA | Qianwei Xing | ENSG00000275183 | -0.04882 |
| Model-50 | 34419719 | mRNA | Qianwei Xing | ENSG00000134627 | 1.07308 |
| Model-50 | 34419719 | mRNA | Qianwei Xing | ENSG00000242265 | 0.03785 |
| Model-50 | 34419719 | mRNA | Qianwei Xing | ENSG00000172346 | 0.30922 |
| Model-50 | 34419719 | mRNA | Qianwei Xing | ENSG00000130589 | 0.09438 |
| Model-50 | 34419719 | mRNA | Qianwei Xing | ENSG00000048740 | -0.22987 |
| Model-50 | 34419719 | mRNA | Qianwei Xing | ENSG00000006047 | -1.56846 |
| Model-50 | 34419719 | mRNA | Qianwei Xing | ENSG00000103067 | -0.06432 |
| Model-51 | 32566639 | mRNA | Yutao Wang | ENSG00000215784 | 1.158 |
| Model-51 | 32566639 | mRNA | Yutao Wang | ENSG00000004777 | 1.737 |
| Model-51 | 32566639 | mRNA | Yutao Wang | ENSG00000075073 | -0.737 |
| Model-51 | 32566639 | mRNA | Yutao Wang | ENSG00000100558 | -0.651 |
| Model-51 | 32566639 | mRNA | Yutao Wang | ENSG00000103089 | -0.793 |
| Model-52 | 33281882 | mRNA | Enchong Zhang | ENSG00000196132 | 0.181 |
| Model-52 | 33281882 | mRNA | Enchong Zhang | ENSG00000188958 | 0.188 |
| Model-52 | 33281882 | mRNA | Enchong Zhang | ENSG00000164076 | 0.235 |
| Model-52 | 33281882 | mRNA | Enchong Zhang | ENSG00000130032 | -0.122 |
| Model-52 | 33281882 | mRNA | Enchong Zhang | ENSG00000105852 | -0.055 |
| Model-52 | 33281882 | mRNA | Enchong Zhang | ENSG00000147255 | -0.017 |
| Model-53 | 36103249 | mRNA | Jin Liu | ENSG00000197361 | -0.37466 |
| Model-53 | 36103249 | mRNA | Jin Liu | ENSG00000182979 | 0.907 |
| Model-53 | 36103249 | mRNA | Jin Liu | ENSG00000141510 | -0.2111 |
| Model-53 | 36103249 | mRNA | Jin Liu | ENSG00000143365 | -0.49317 |
| Model-53 | 36103249 | mRNA | Jin Liu | ENSG00000069696 | 0.47302 |
| Model-53 | 36103249 | mRNA | Jin Liu | ENSG00000109819 | -0.23153 |
| Model-53 | 36103249 | mRNA | Jin Liu | ENSG00000140836 | -0.52212 |
| Model-54 | 33329725 | mRNA | Qijie Zhang | ENSG00000181790 | 1.3207 |
| Model-54 | 33329725 | mRNA | Qijie Zhang | ENSG00000163132 | 0.9112 |
| Model-54 | 33329725 | mRNA | Qijie Zhang | ENSG00000165802 | 4.063 |
| Model-54 | 33329725 | mRNA | Qijie Zhang | ENSG00000103496 | 8.9674 |
| Model-54 | 33329725 | mRNA | Qijie Zhang | ENSG00000147889 | 0.9265 |
| Model-54 | 33329725 | mRNA | Qijie Zhang | ENSG00000178999 | 2.6753 |
| Model-54 | 33329725 | mRNA | Qijie Zhang | ENSG00000069974 | 1.9525 |
| Model-54 | 33329725 | mRNA | Qijie Zhang | ENSG00000089248 | -5.4224 |
| Model-54 | 33329725 | mRNA | Qijie Zhang | ENSG00000198417 | -1.1641 |
| Model-54 | 33329725 | mRNA | Qijie Zhang | ENSG00000101412 | -3.2353 |
| Model-54 | 33329725 | mRNA | Qijie Zhang | ENSG00000174307 | -2.3318 |
| Model-54 | 33329725 | mRNA | Qijie Zhang | ENSG00000142405 | -1.6184 |
| Model-54 | 33329725 | mRNA | Qijie Zhang | ENSG00000133112 | -4.6031 |
| Model-54 | 33329725 | mRNA | Qijie Zhang | ENSG00000159388 | -2.2351 |
| Model-54 | 33329725 | mRNA | Qijie Zhang | ENSG00000185122 | -4.1958 |
| Model-55 | 32425694 | mRNA | Yongzhi Wang | ENSG00000093009 | 0.1562 |
| Model-55 | 32425694 | mRNA | Yongzhi Wang | ENSG00000135476 | 0.0285 |
| Model-55 | 32425694 | mRNA | Yongzhi Wang | ENSG00000085999 | 0.00011 |
| Model-56 | 35799610 | mRNA | Bohan Fan | ENSG00000038427 | 0.29072 |
| Model-56 | 35799610 | mRNA | Bohan Fan | ENSG00000184916 | 0.49566 |
| Model-56 | 35799610 | mRNA | Bohan Fan | ENSG00000099250 | 0.20856 |
| Model-57 | 35592542 | mRNA | Jingchao Wei | ENSG00000002726 | 0.85 |
| Model-57 | 35592542 | mRNA | Jingchao Wei | ENSG00000132437 | 1.2 |
| Model-57 | 35592542 | mRNA | Jingchao Wei | ENSG00000231106 | 1.26 |
| Model-57 | 35592542 | mRNA | Jingchao Wei | ENSG00000229314 | 1.18 |
| Model-57 | 35592542 | mRNA | Jingchao Wei | ENSG00000171759 | 0.8 |
| Model-58 | 34595101 | mRNA | Kaixuan Guo | ENSG00000072571 | 0.07146 |
| Model-58 | 34595101 | mRNA | Kaixuan Guo | ENSG00000112984 | 0.06978 |
| Model-58 | 34595101 | mRNA | Kaixuan Guo | ENSG00000138271 | -0.04675 |
| Model-58 | 34595101 | mRNA | Kaixuan Guo | ENSG00000165434 | 0.07757 |
| Model-58 | 34595101 | mRNA | Kaixuan Guo | ENSG00000163516 | 0.09192 |
| Model-59 | 30024105 | mRNA | Yanzhi Jiang | ENSG00000174640 | 1.5813 |
| Model-59 | 30024105 | mRNA | Yanzhi Jiang | ENSG00000128849 | 0.9902 |
| Model-59 | 30024105 | mRNA | Yanzhi Jiang | ENSG00000156502 | 0.8437 |
| Model-59 | 30024105 | mRNA | Yanzhi Jiang | ENSG00000157014 | 1.3132 |
| Model-59 | 30024105 | mRNA | Yanzhi Jiang | ENSG00000161013 | 1.5178 |
| Model-59 | 30024105 | mRNA | Yanzhi Jiang | ENSG00000160293 | 1.1027 |
| Model-59 | 30024105 | mRNA | Yanzhi Jiang | ENSG00000171612 | 1.096 |
| Model-59 | 30024105 | mRNA | Yanzhi Jiang | ENSG00000078070 | 0.8336 |
| Model-59 | 30024105 | mRNA | Yanzhi Jiang | ENSG00000070669 | 1.3456 |
| Model-59 | 30024105 | mRNA | Yanzhi Jiang | ENSG00000167971 | 1.0286 |
| Model-59 | 30024105 | mRNA | Yanzhi Jiang | ENSG00000088305 | 1.2919 |
| Model-59 | 30024105 | mRNA | Yanzhi Jiang | ENSG00000087586 | 1.0966 |
| Model-59 | 30024105 | mRNA | Yanzhi Jiang | ENSG00000104147 | 1.365 |
| Model-59 | 30024105 | mRNA | Yanzhi Jiang | ENSG00000164932 | 0.7981 |
| Model-59 | 30024105 | mRNA | Yanzhi Jiang | ENSG00000155265 | 2.0406 |
| Model-60 | 36675580 | mRNA | Zhongyou Xia | ENSG00000130203 | 0.29475 |
| Model-60 | 36675580 | mRNA | Zhongyou Xia | ENSG00000124721 | 0.07153 |
| Model-60 | 36675580 | mRNA | Zhongyou Xia | ENSG00000197774 | 0.48194 |
| Model-60 | 36675580 | mRNA | Zhongyou Xia | ENSG00000155980 | 0.15806 |
| Model-61 | 34490029 | mRNA | Min Fu | ENSG00000258947 | 0.20811 |
| Model-61 | 34490029 | mRNA | Min Fu | ENSG00000096384 | 0.35558 |
| Model-61 | 34490029 | mRNA | Min Fu | ENSG00000087266 | 0.28907 |
| Model-61 | 34490029 | mRNA | Min Fu | ENSG00000175166 | 0.35296 |
| Model-61 | 34490029 | mRNA | Min Fu | ENSG00000136813 | 0.19746 |
| Model-61 | 34490029 | mRNA | Min Fu | ENSG00000198753 | 0.39878 |
| Model-61 | 34490029 | mRNA | Min Fu | ENSG00000164406 | 0.18064 |
| Model-61 | 34490029 | mRNA | Min Fu | ENSG00000086991 | 0.15682 |
| Model-61 | 34490029 | mRNA | Min Fu | ENSG00000186350 | 0.45767 |
| Model-61 | 34490029 | mRNA | Min Fu | ENSG00000089685 | 0.02817 |
| Model-61 | 34490029 | mRNA | Min Fu | ENSG00000168056 | 0.28738 |
| Model-61 | 34490029 | mRNA | Min Fu | ENSG00000106991 | 0.33681 |
| Model-61 | 34490029 | mRNA | Min Fu | ENSG00000166710 | -0.05744 |
| Model-61 | 34490029 | mRNA | Min Fu | ENSG00000134352 | -0.33915 |
| Model-61 | 34490029 | mRNA | Min Fu | ENSG00000162444 | -0.10282 |
| Model-61 | 34490029 | mRNA | Min Fu | ENSG00000164761 | -0.55779 |
| Model-61 | 34490029 | mRNA | Min Fu | ENSG00000173530 | -0.2254 |
| Model-61 | 34490029 | mRNA | Min Fu | ENSG00000124205 | -0.08863 |
| Model-62 | 35669428 | mRNA | Chun Li | ENSG00000030110 | 0.598 |
| Model-62 | 35669428 | mRNA | Chun Li | ENSG00000087088 | 0.223 |
| Model-62 | 35669428 | mRNA | Chun Li | ENSG00000147457 | 0.8 |
| Model-62 | 35669428 | mRNA | Chun Li | ENSG00000073605 | -0.863 |
| Model-62 | 35669428 | mRNA | Chun Li | ENSG00000091592 | -0.155 |
| Model-63 | 33995646 | mRNA | Jiao-Chen Luan | ENSG00000072736 | -1.0121 |
| Model-63 | 33995646 | mRNA | Jiao-Chen Luan | ENSG00000130513 | -0.096 |
| Model-63 | 33995646 | mRNA | Jiao-Chen Luan | ENSG00000115594 | -0.0374 |
| Model-63 | 33995646 | mRNA | Jiao-Chen Luan | ENSG00000258947 | 0.0334 |
| Model-63 | 33995646 | mRNA | Jiao-Chen Luan | ENSG00000099250 | 0.1937 |
| Model-63 | 33995646 | mRNA | Jiao-Chen Luan | ENSG00000139874 | 0.6039 |
| Model-64 | 31632503 | mRNA | Zhe-Xu Cao | ENSG00000108848 | 0.006 |
| Model-64 | 31632503 | mRNA | Zhe-Xu Cao | ENSG00000064607 | 0.023 |
| Model-64 | 31632503 | mRNA | Zhe-Xu Cao | ENSG00000115524 | -0.009 |
| Model-64 | 31632503 | mRNA | Zhe-Xu Cao | ENSG00000101439 | -0.002 |
| Model-64 | 31632503 | mRNA | Zhe-Xu Cao | ENSG00000137073 | 0.059 |
| Model-64 | 31632503 | mRNA | Zhe-Xu Cao | ENSG00000137135 | 0.15 |
| Model-65 | 36810782 | mRNA | Qiliang Zhai | ENSG00000161960 | 0.20456 |
| Model-65 | 36810782 | mRNA | Qiliang Zhai | ENSG00000114503 | 0.03135 |
| Model-66 | 37188204 | mRNA | Bangwei Che | ENSG00000004468 | 0.77499 |
| Model-66 | 37188204 | mRNA | Bangwei Che | ENSG00000198467 | 0.88244 |
| Model-66 | 37188204 | mRNA | Bangwei Che | ENSG00000187193 | 0.88027 |
| Model-66 | 37188204 | mRNA | Bangwei Che | ENSG00000096006 | 0.92986 |
| Model-66 | 37188204 | mRNA | Bangwei Che | ENSG00000101335 | 1.37342 |
| Model-66 | 37188204 | mRNA | Bangwei Che | ENSG00000065534 | 0.69256 |
| Model-67 | 35860569 | lncRNA | Liansha Tang | ENSG00000168994 | -0.89 |
| Model-67 | 35860569 | lncRNA | Liansha Tang | ENSG00000139220 | 0.08 |
| Model-67 | 35860569 | lncRNA | Liansha Tang | ENSG00000250056 | -0.65 |
| Model-67 | 35860569 | lncRNA | Liansha Tang | ENSG00000280323 | 0.14 |
| Model-67 | 35860569 | lncRNA | Liansha Tang | ENSG00000185442 | 0.32 |
| Model-67 | 35860569 | lncRNA | Liansha Tang | ENSG00000143190 | 0.7 |
| Model-67 | 35860569 | lncRNA | Liansha Tang | ENSG00000164687 | -0.69 |
| Model-67 | 35860569 | lncRNA | Liansha Tang | ENSG00000258274 | -0.52 |
| Model-67 | 35860569 | lncRNA | Liansha Tang | ENSG00000250266 | 0.1 |
| Model-67 | 35860569 | lncRNA | Liansha Tang | ENSG00000233308 | 0.07 |
| Model-68 | 35186021 | mRNA | Chao Luo | ENSG00000187094 | -0.066 |
| Model-68 | 35186021 | mRNA | Chao Luo | ENSG00000004468 | -0.127 |
| Model-68 | 35186021 | mRNA | Chao Luo | ENSG00000135929 | -0.0615 |
| Model-68 | 35186021 | mRNA | Chao Luo | ENSG00000255150 | -0.833 |
| Model-68 | 35186021 | mRNA | Chao Luo | ENSG00000148702 | 0.088 |
| Model-68 | 35186021 | mRNA | Chao Luo | ENSG00000128594 | 0.473 |
| Model-68 | 35186021 | mRNA | Chao Luo | ENSG00000244355 | -0.122 |
| Model-69 | 33457230 | mRNA | Xiangkun Wu | ENSG00000066279 | 0.68 |
| Model-69 | 33457230 | mRNA | Xiangkun Wu | ENSG00000099953 | 0.79 |
| Model-69 | 33457230 | mRNA | Xiangkun Wu | ENSG00000117724 | 0.51 |
| Model-69 | 33457230 | mRNA | Xiangkun Wu | ENSG00000090889 | 0.14 |
| Model-69 | 33457230 | mRNA | Xiangkun Wu | ENSG00000108821 | 1.17 |
| Model-69 | 33457230 | mRNA | Xiangkun Wu | ENSG00000106819 | 0.39 |
| Model-69 | 33457230 | mRNA | Xiangkun Wu | ENSG00000140525 | 1.11 |
| Model-69 | 33457230 | mRNA | Xiangkun Wu | ENSG00000188959 | -1.59 |
| Model-69 | 33457230 | mRNA | Xiangkun Wu | ENSG00000120915 | -1.29 |
| Model-70 | 33758613 | mRNA | Hao Zhao | ENSG00000159189 | 0.0641 |
| Model-70 | 33758613 | mRNA | Hao Zhao | ENSG00000108821 | 0.00467 |
| Model-70 | 33758613 | mRNA | Hao Zhao | ENSG00000171476 | 0.01777 |
| Model-70 | 33758613 | mRNA | Hao Zhao | ENSG00000140678 | 0.17512 |
| Model-70 | 33758613 | mRNA | Hao Zhao | ENSG00000010327 | 0.35768 |
| Model-70 | 33758613 | mRNA | Hao Zhao | ENSG00000105329 | 0.13676 |
| Model-70 | 33758613 | mRNA | Hao Zhao | ENSG00000175336 | -0.04815 |
| Model-70 | 33758613 | mRNA | Hao Zhao | ENSG00000120903 | -0.0618 |
| Model-70 | 33758613 | mRNA | Hao Zhao | ENSG00000159212 | -0.22487 |
| Model-70 | 33758613 | mRNA | Hao Zhao | ENSG00000120738 | -0.03191 |
| Model-70 | 33758613 | mRNA | Hao Zhao | ENSG00000163497 | -0.01361 |
| Model-70 | 33758613 | mRNA | Hao Zhao | ENSG00000170345 | -0.00269 |
| Model-70 | 33758613 | mRNA | Hao Zhao | ENSG00000169562 | -0.0647 |
| Model-70 | 33758613 | mRNA | Hao Zhao | ENSG00000186469 | -0.23936 |
| Model-70 | 33758613 | mRNA | Hao Zhao | ENSG00000117594 | -0.10059 |
| Model-70 | 33758613 | mRNA | Hao Zhao | ENSG00000116774 | -0.03021 |
| Model-70 | 33758613 | mRNA | Hao Zhao | ENSG00000100979 | -0.06134 |
| Model-70 | 33758613 | mRNA | Hao Zhao | ENSG00000125780 | -0.0394 |
| Model-71 | 33954109 | mRNA | Zijun Xu | ENSG00000111713 | -0.8367 |
| Model-71 | 33954109 | mRNA | Zijun Xu | ENSG00000117632 | 0.3448 |
| Model-71 | 33954109 | mRNA | Zijun Xu | ENSG00000143847 | 0.3595 |
| Model-71 | 33954109 | mRNA | Zijun Xu | ENSG00000100196 | -0.194 |
| Model-71 | 33954109 | mRNA | Zijun Xu | ENSG00000115657 | 0.4779 |
| Model-72 | 34966582 | mRNA | Wenchang Yue | ENSG00000141551 | 0.8 |
| Model-72 | 34966582 | mRNA | Wenchang Yue | ENSG00000166167 | -1.824 |
| Model-72 | 34966582 | mRNA | Wenchang Yue | ENSG00000134852 | -1.7645 |
| Model-72 | 34966582 | mRNA | Wenchang Yue | ENSG00000213923 | 0.4555 |
| Model-72 | 34966582 | mRNA | Wenchang Yue | ENSG00000005812 | -1.239 |
| Model-72 | 34966582 | mRNA | Wenchang Yue | ENSG00000162409 | -1.56 |
| Model-72 | 34966582 | mRNA | Wenchang Yue | ENSG00000105516 | 1.325 |
| Model-72 | 34966582 | mRNA | Wenchang Yue | ENSG00000174738 | 0.433 |
| Model-72 | 34966582 | mRNA | Wenchang Yue | ENSG00000198963 | 1.049 |
| Model-73 | 36982591 | mRNA | Qi You | ENSG00000089280 | 0.29615 |
| Model-73 | 36982591 | mRNA | Qi You | ENSG00000173894 | 0.24877 |
| Model-73 | 36982591 | mRNA | Qi You | ENSG00000088325 | 0.24194 |
| Model-73 | 36982591 | mRNA | Qi You | ENSG00000102125 | 0.36367 |
| Model-73 | 36982591 | mRNA | Qi You | ENSG00000006611 | 0.44257 |
| Model-73 | 36982591 | mRNA | Qi You | ENSG00000103126 | 0.50295 |
| Model-74 | 34453418 | mRNA | Qiang Su | ENSG00000086991 | 0.04604 |
| Model-74 | 34453418 | mRNA | Qiang Su | ENSG00000131187 | 0.04381 |
| Model-74 | 34453418 | mRNA | Qiang Su | ENSG00000088325 | 0.0662 |
| Model-74 | 34453418 | mRNA | Qiang Su | ENSG00000175287 | -0.02754 |
| Model-74 | 34453418 | mRNA | Qiang Su | ENSG00000087586 | 0.06883 |
| Model-74 | 34453418 | mRNA | Qiang Su | ENSG00000058799 | -0.01182 |
| Model-75 | 35620461 | mRNA | Shuqiang Li | ENSG00000167702 | 0.12467 |
| Model-75 | 35620461 | mRNA | Shuqiang Li | ENSG00000064787 | -0.0915 |
| Model-76 | 33343639 | mRNA | Jiaju Xu | ENSG00000122566 | 0.00985 |
| Model-76 | 33343639 | mRNA | Jiaju Xu | ENSG00000162231 | 0.02761 |
| Model-76 | 33343639 | mRNA | Jiaju Xu | ENSG00000147274 | 0.0048 |
| Model-76 | 33343639 | mRNA | Jiaju Xu | ENSG00000149658 | 0.01969 |
| Model-76 | 33343639 | mRNA | Jiaju Xu | ENSG00000173113 | 0.00207 |
| Model-77 | 34631510 | mRNA | Rui Zhou | ENSG00000077684 | 0.0048 |
| Model-77 | 34631510 | mRNA | Rui Zhou | ENSG00000100373 | -0.0144 |
| Model-77 | 34631510 | mRNA | Rui Zhou | ENSG00000112773 | 0.0326 |
| Model-77 | 34631510 | mRNA | Rui Zhou | ENSG00000116039 | -0.0206 |
| Model-77 | 34631510 | mRNA | Rui Zhou | ENSG00000140279 | -0.0091 |
| Model-77 | 34631510 | mRNA | Rui Zhou | ENSG00000164850 | 0.0668 |
| Model-77 | 34631510 | mRNA | Rui Zhou | ENSG00000180739 | -0.0054 |
| Model-77 | 34631510 | mRNA | Rui Zhou | ENSG00000181350 | 0.002 |
| Model-77 | 34631510 | mRNA | Rui Zhou | ENSG00000197757 | 0.0246 |
| Model-77 | 34631510 | mRNA | Rui Zhou | ENSG00000206052 | 0.0337 |
| Model-78 | 30637711 | mRNA | F Li | ENSG00000267795 | -0.744 |
| Model-78 | 30637711 | mRNA | F Li | ENSG00000169891 | -0.809 |
| Model-78 | 30637711 | mRNA | F Li | ENSG00000162341 | 0.568 |
| Model-78 | 30637711 | mRNA | F Li | ENSG00000101004 | 0.681 |
| Model-78 | 30637711 | mRNA | F Li | ENSG00000131747 | 0.686 |
| Model-78 | 30637711 | mRNA | F Li | ENSG00000158458 | -0.962 |
| Model-79 | 36643625 | mRNA | Hongbo Wang | ENSG00000197894 | -0.25 |
| Model-79 | 36643625 | mRNA | Hongbo Wang | ENSG00000186350 | 0.25 |
| Model-79 | 36643625 | mRNA | Hongbo Wang | ENSG00000110958 | 0.22 |
| Model-79 | 36643625 | mRNA | Hongbo Wang | ENSG00000180902 | 0.24 |
| Model-79 | 36643625 | mRNA | Hongbo Wang | ENSG00000140876 | -0.24 |
| Model-79 | 36643625 | mRNA | Hongbo Wang | ENSG00000120915 | -0.07 |
| Model-79 | 36643625 | mRNA | Hongbo Wang | ENSG00000116882 | 0.16 |
| Model-79 | 36643625 | mRNA | Hongbo Wang | ENSG00000169169 | 0.56 |
| Model-79 | 36643625 | mRNA | Hongbo Wang | ENSG00000213316 | 0.09 |
| Model-79 | 36643625 | mRNA | Hongbo Wang | ENSG00000100372 | 0.17 |
| Model-80 | 36824662 | lncRNA | Yutao Wang | ENSG00000260272 | 0.71 |
| Model-80 | 36824662 | lncRNA | Yutao Wang | ENSG00000171735 | 0.2 |
| Model-80 | 36824662 | lncRNA | Yutao Wang | ENSG00000223764 | 0.12 |
| Model-80 | 36824662 | lncRNA | Yutao Wang | ENSG00000249984 | -1.55 |
| Model-80 | 36824662 | lncRNA | Yutao Wang | ENSG00000251442 | 0.79 |
| Model-80 | 36824662 | lncRNA | Yutao Wang | ENSG00000167195 | 0.2 |
| Model-80 | 36824662 | lncRNA | Yutao Wang | ENSG00000164172 | -0.62 |
| Model-80 | 36824662 | lncRNA | Yutao Wang | ENSG00000274386 | -0.62 |
| Model-80 | 36824662 | lncRNA | Yutao Wang | ENSG00000254536 | 0.16 |
